# Signatures of COVID-19 Severity and Immune Response in the Respiratory Tract Microbiome

**DOI:** 10.1128/mBio.01777-21

**Published:** 2021-08-17

**Authors:** Carter Merenstein, Guanxiang Liang, Samantha A. Whiteside, Ana G. Cobián-Güemes, Madeline S. Merlino, Louis J. Taylor, Abigail Glascock, Kyle Bittinger, Ceylan Tanes, Jevon Graham-Wooten, Layla A. Khatib, Ayannah S. Fitzgerald, Shantan Reddy, Amy E. Baxter, Josephine R. Giles, Derek A. Oldridge, Nuala J. Meyer, E. John Wherry, John E. McGinniss, Frederic D. Bushman, Ronald G. Collman

**Affiliations:** a Department of Microbiology, University of Pennsylvania Perelman School of Medicine, Philadelphia, Pennsylvania, USA; b Pulmonary, Allergy and Critical Care Division, Department of Medicine, University of Pennsylvania Perelman School of Medicine, Philadelphia, Pennsylvania, USA; c Division of Gastroenterology, Hepatology, and Nutrition, Children's Hospital of Philadelphia, Philadelphia, Pennsylvania, USA; d Department of Systems Pharmacology and Translational Therapeutics, University of Pennsylvania Perelman School of Medicine, Philadelphia, Pennsylvania, USA; e Institute for Immunology, University of Pennsylvania Perelman School of Medicine, Philadelphia, Pennsylvania, USA; f Department of Pathology and Laboratory Medicine, University of Pennsylvania Perelman School of Medicine, Philadelphia, Pennsylvania, USA; University of Michigan-Ann Arbor

**Keywords:** SARS-CoV-2, coronavirus, 16S rRNA gene sequencing, redondovirus, anellovirus, respiratory microbiome

## Abstract

Viral infection of the respiratory tract can be associated with propagating effects on the airway microbiome, and microbiome dysbiosis may influence viral disease. Here, we investigated the respiratory tract microbiome in coronavirus disease 2019 (COVID-19) and its relationship to disease severity, systemic immunologic features, and outcomes. We examined 507 oropharyngeal, nasopharyngeal, and endotracheal samples from 83 hospitalized COVID-19 patients as well as non-COVID patients and healthy controls. Bacterial communities were interrogated using 16S rRNA gene sequencing, and the commensal DNA viruses *Anelloviridae* and *Redondoviridae* were quantified by qPCR. We found that COVID-19 patients had upper respiratory microbiome dysbiosis and greater change over time than critically ill patients without COVID-19. Oropharyngeal microbiome diversity at the first time point correlated inversely with disease severity during hospitalization. Microbiome composition was also associated with systemic immune parameters in blood, as measured by lymphocyte/neutrophil ratios and immune profiling of peripheral blood mononuclear cells. Intubated patients showed patient-specific lung microbiome communities that were frequently highly dynamic, with prominence of Staphylococcus. *Anelloviridae* and *Redondoviridae* showed more frequent colonization and higher titers in severe disease. Machine learning analysis demonstrated that integrated features of the microbiome at early sampling points had high power to discriminate ultimate level of COVID-19 severity. Thus, the respiratory tract microbiome and commensal viruses are disturbed in COVID-19 and correlate with systemic immune parameters, and early microbiome features discriminate disease severity. Future studies should address clinical consequences of airway dysbiosis in COVID-19, its possible use as biomarkers, and the role of bacterial and viral taxa identified here in COVID-19 pathogenesis.

## INTRODUCTION

Coronavirus disease 2019 (COVID-19), caused by severe acute respiratory syndrome coronavirus 2 (SARS-CoV-2), is a global pandemic with severe morbidity and mortality and unprecedented economic and social disruption. A striking feature of COVID-19 is the wide variance in clinical severity among infected people. Many factors correlate with COVID-19 disease severity, including age, gender, body mass index, prior comorbidities, immune responses, and genetics (1–3), yet the determinants of infection outcome and pathogenic mechanisms are incompletely understood. Here, we investigated the potential relationship between COVID-19 severity and the microbiome of the respiratory tract.

The respiratory tract is the site of initial SARS-CoV-2 infection and the most common site of serious clinical manifestations. Infection of any mucosal surface occurs in the context of its endogenous microbiome, and bidirectional interactions between host and microbiota commonly contribute to infection and pathogenesis. For example, influenza predisposes to secondary bacterial infection in the respiratory tract, which is responsible for much of its morbidity and mortality (4–7). Conversely, prior disruption of the normal microbiome can influence susceptibility to or pathogenesis of respiratory viruses, such as influenza virus and respiratory syncytial virus (8–10). Few studies have addressed the respiratory tract microbiome in COVID-19 or links to outcome, though early data report evidence of dysbiosis (11, 12).

Here, we investigated signatures of COVID-19 disease in the respiratory tract microbiome, analyzing 507 oropharyngeal, nasopharyngeal, and endotracheal specimens from 83 hospitalized COVID-19 patients. We also collected 75 specimens from 13 critically ill patients hospitalized for other disorders. Bacterial community composition was assessed using 16S rRNA gene sequencing. Levels of commensal viruses of the human airway, specifically *Anelloviridae* and *Redondoviridae*, were quantified using qPCR. These small circular DNA viruses have been reported to vary in abundance in association with disease states and/or immune competence (13–15), so we reasoned that they might report aspects of COVID-19 disease status. Finally, we queried the relationship between the airway microbiome and immune profiles in blood. Our analysis revealed dysbiosis of the upper and lower respiratory microbiome, differences between COVID-19 and non-COVID patients, associations with systemic inflammation, and microbial signatures distinguishing COVID-19 severity.

## RESULTS

### Subjects, specimens, and SARS-CoV-2 analysis.

The 83 COVID-19 patients (Table 1; also, see Table S1 and Fig. S1 in the supplemental material) had a median age of 64 years (range, 36 to 91) and included 39 women and 44 men. Fifty-six were Black (67%), 20 were white (24%), 3 were Asian (4%), and 4 were unknown/other race (5%). All but 5 had at least one underlying major organ system comorbidity. Forty (48%) required intubation and invasive mechanical ventilation, and 20 (24%) died. Each patients' clinical course was classified by maximal severity reached during hospitalization based on the WHO 11-point scale (16), in which hospitalized patients are level 4 or above, intubated subjects are level 7 or above, and fatal outcomes are 10. Non-COVID critically ill patients (*n* = 13) exhibited a variety of underlying diseases; 62% required intubation, and 6 (46%) died. Upper respiratory tract (oropharyngeal [OP] and nasopharyngeal [NP]) and lung (endotracheal aspirate [ETA]) sampling was carried out serially, yielding a total of 582 specimens for microbiome analysis (506 COVID-19 and 73 non-COVID). Healthy volunteers provided NP and OP (*n* = 30) and lung (bronchoalveolar lavage [BAL]; *n* = 12) specimens (17–19).

**TABLE 1:** Patient characteristics

Characteristic^*a*^	Value for group	
	COVID-19 (*n* = 83)	Non-COVID (*n* = 13)
Gender [no. (%)]		
Female	39 (47)	4 (31)
Male	44 (53)	9 (69)
Race/ethnicity [no. (%)]		
Black	56 (67)	7 (54)
White	20 (24)	6 (46)
Asian	3 (4)	0
Other/unknown	4 (5)	0
Hispanic/Latinx	0	1 (8)
Age		
Median (min, max)	64 (36–91)	60 (39–94)
BMI		
Median (min, max)	29.8 (17–62)	23.0 (19–31)
No. (%) with preexisting comorbidity		
Diabetes	39 (47)	6 (46)
Hypertension	67 (81)	7 (54)
Coronary artery disease	16 (19)	3 (23)
Stroke	17 (20)	2 (15)
Chronic lung disease	34 (41)	5 (38)
Renal disease (≥stage 4)	15 (18)	3 (23)
Cancer (within 6 mo)	10 (12)	4 (31)
HIV infection	3 (4)	0
Organ transplant	5 (6)	1 (8)
Immunosuppressive therapy	12 (14)	3 (23)
BMI ≥35	27 (33)	0
Any major comorbidity	78 (94)	13 (100)
No. (%) receiving treatment		
Corticosteroids	52 (63)	
Remdesivir	18 (22)	
Hydroxychloroquine	39 (47)	
Convalescent-phase plasma	4 (5)	
Antibacterials	72 (87)	13 (100)
Antifungals	20 (24)	5 (38)
Mechanical ventilation	40 (48)	8 (62)
ECMO	5 (6)	1 (8)
No. (%) with maximum WHO score (example)		
4 (no supplemental O_2_)	20 (24)	
5 (low-flow O_2_)	8 (10)	
6 (high-flow O_2_)	7 (8)	
7 (intubated)	2 (2)	
8 (intubated, low P/F; vasopressors)	14 (17)	
9 (ECMO; pressors; dialysis)	12 (14)	
10 (death)	20 (24)	6 (46)

a BMI, body mass index; ECMO, extracorporeal membrane oxygenation; P/F, ratio of PaO_2_ to FiO_2_.

10.1128/mBio.01777-21.2 FIG S1 Subject timelines and antibiotic administration. Subjects are grouped by COVID-19 versus non-COVID status. Light gray boxes indicate period of hospitalization, and dark gray boxes indicate period of sampling. The *x* axis indicates time from hospitalization. Maximum COVID-19 disease severity based on WHO score is indicated with subject identifiers, and patients who died (WHO score of 10) are indicated with asterisks. Antibiotic administration is shown as colored horizontal bars. For simplicity, some antibiotics are grouped with the most common agent within a particular class, as indicated by a plus sign: Cefazolin+ includes cefalexin and cefadroxil; Ceftriaxone+ includes ceftazidime and cefpodoxime; Meropenem+ includes ertapenem and meropenem-vaborbactam. Antifungals include caspofungin, fluconazole, isavuconazonium, posaconazole, voriconazole, and atovaquone. “Other” indicates less commonly used antibiotics, including amoxicillin, aztreonam, ceftaroline, clindamycin, colistin, fosfomycin, minocycline, amoxicillin-clavulanate, ceftolozane-tazobactam, ampicillin, and ampicillin-sulbactam. Download FIG S1, PDF file, 0.3 MB. Copyright © 2021 Merenstein et al. 2021 Merenstein et al. https://creativecommons.org/licenses/by/4.0/ This content is distributed under the terms of the Creative Commons Attribution 4.0 International license.

10.1128/mBio.01777-21.5 TABLE S1 Enrolled subjects. Download Table S1, PDF file, 0.1 MB. Copyright © 2021 Merenstein et al. 2021 Merenstein et al. https://creativecommons.org/licenses/by/4.0/ This content is distributed under the terms of the Creative Commons Attribution 4.0 International license.

SARS-CoV-2 RNA levels were variable among subjects and sample types (Fig. S2). As expected, SARS-CoV-2 RNA levels declined to undetectable levels over time in most patients, although several had persistently detectable RNA in ETA beyond 3 weeks after symptom onset. There was no association between SARS-CoV-2 RNA levels and WHO score or clinical outcomes (Wilcoxon rank sum test). Complete SARS-CoV-2 genome sequences were determined for 26 subjects as recently reported (20). All viral genomes were members of the B.1 lineage, which encodes the D614G variant in spike, and most also had the P314L variant in the RNA-dependent RNA polymerase (RdRp) located on ORF1b (21, 22).

10.1128/mBio.01777-21.3 FIG S2 SARS-CoV-2 viral RNA levels in respiratory tract samples. Levels of SARS-CoV-2 were determined by qPCR. Sample type is coded by color, and samples of the same type from the same subject are connected by lines. Download FIG S2, PDF file, 0.2 MB. Copyright © 2021 Merenstein et al. 2021 Merenstein et al. https://creativecommons.org/licenses/by/4.0/ This content is distributed under the terms of the Creative Commons Attribution 4.0 International license.

### Respiratory tract bacterial dysbiosis in COVID-19.

**FIG 1:**
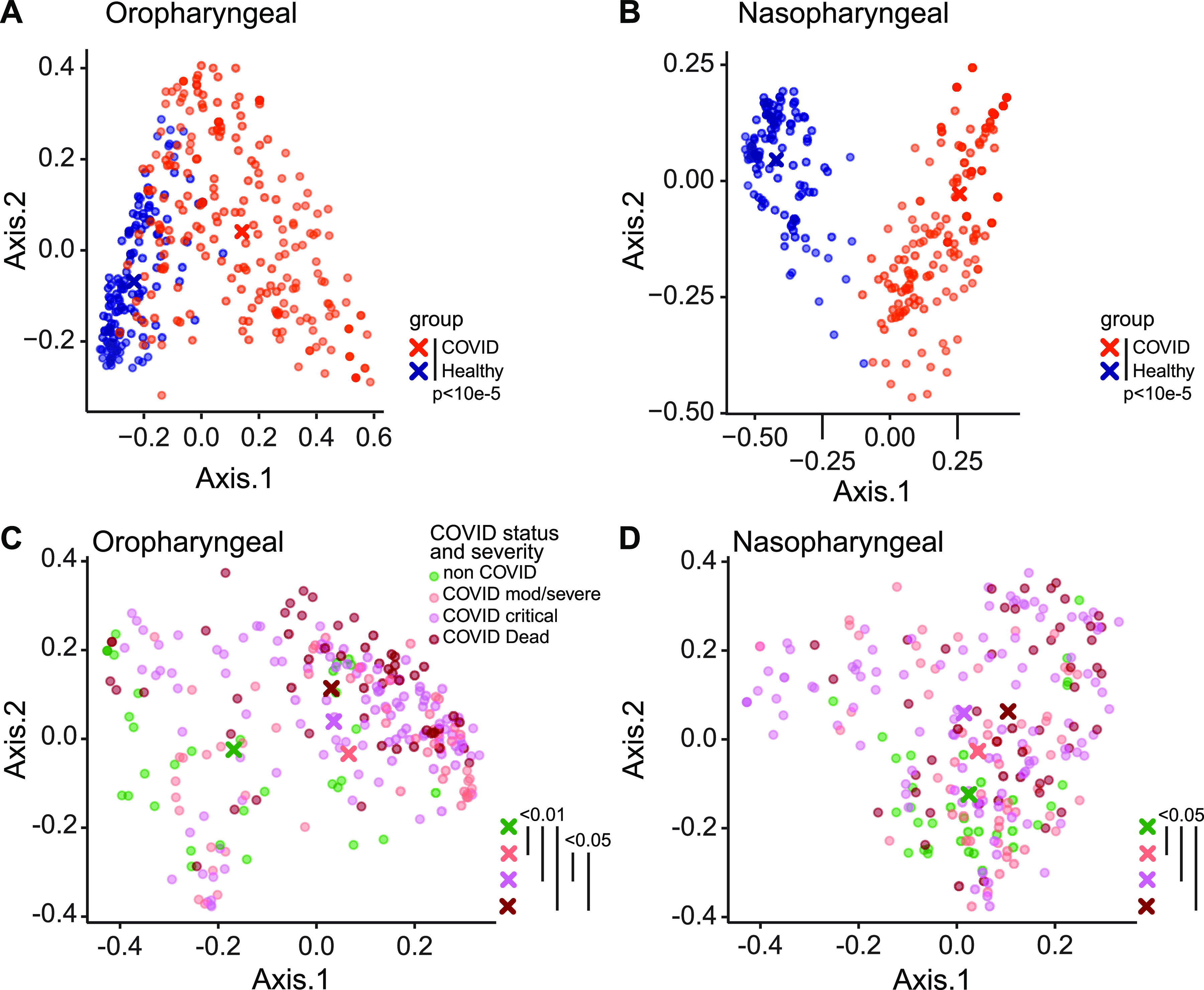
Upper respiratory tract dysbiosis in COVID-19 patients. Bacterial communities in the oropharynx (A and C) and nasopharynx (B and D) were analyzed by unweighted UniFrac. For ease of visualization, data for COVID-19 patients and healthy individuals are shown in panels A and B, while panels C and D show data for hospitalized COVID-19 and non-COVID patients, with COVID-19 subjects grouped by disease severity as moderate/severe (WHO 4 to 6), critical (WHO 7 to 9), and fatal (WHO 10). Each dot represents an individual community, and the centroid for each subject group is indicated with a “×” sign. Values for all samples are shown; *P* values were generated using random subsampling for each subject. Both COVID and non-COVID were significantly different from healthy (*P* < 10^−5^; PERMANOVA), and significant differences between groups (indicated by color-coded “×”) are shown on the right of each plot.

Bacterial communities were interrogated using primers targeting the V1V2 region of the 16S rRNA gene, which has been employed extensively for airway samples (23, 24) (Fig. 1; Fig. S3A and B; Table S2). Oropharyngeal and nasopharyngeal communities of COVID-19 patients differed markedly from those of healthy subjects using the unweighted UniFrac metric, which compares samples based on bacterial presence-absence information (Fig. 1A and B) (*P* < 0.00001 for both OP and NP samples). We then compared COVID-19 to non-COVID patients and compared COVID-19 patients to each other based on maximal severity during hospitalization. COVID-19 patients were grouped as WHO 4 to 6 (moderate/severe, nonintubated), WHO 7 to 9 (critical/intubated), and WHO 10 (fatal) (Fig. 1B and C). In results for both oropharynx and nasopharynx samples, there was significant separation between groups. In pairwise comparisons, all COVID-19 groups were significantly different from the non-COVID group (false discovery rate [FDR], <0.01 for OP and <0.05 for NP samples). Oropharyngeal swabs also showed separation between COVID-19 patients with moderate/severe (WHO 4 to 6) and critical/fatal (WHO 7 to 10) outcomes (FDR < 0.06).

10.1128/mBio.01777-21.4 FIG S3 Bacterial communities in oropharyngeal and nasopharyngeal samples and relationship to clinical outcomes. (A and B) Heat maps showing oropharyngeal (A) and nasopharyngeal (B) communities. (C) Bacterial taxa present in first sample that are significantly associated with clinical status over the course of hospitalization. The *x* axis shows the WHO score; the *y* axis shows the percentage of the community composed by the indicated genus in the first sample. Sample type and FDR-corrected *P* values are shown at the top. Download FIG S3, PDF file, 0.4 MB. Copyright © 2021 Merenstein et al. 2021 Merenstein et al. https://creativecommons.org/licenses/by/4.0/ This content is distributed under the terms of the Creative Commons Attribution 4.0 International license.

10.1128/mBio.01777-21.6 TABLE S2 Samples analyzed by 16S rRNA marker gene sequencing. Download Table S2, PDF file, 0.1 MB. Copyright © 2021 Merenstein et al. 2021 Merenstein et al. https://creativecommons.org/licenses/by/4.0/ This content is distributed under the terms of the Creative Commons Attribution 4.0 International license.

**FIG 2:**
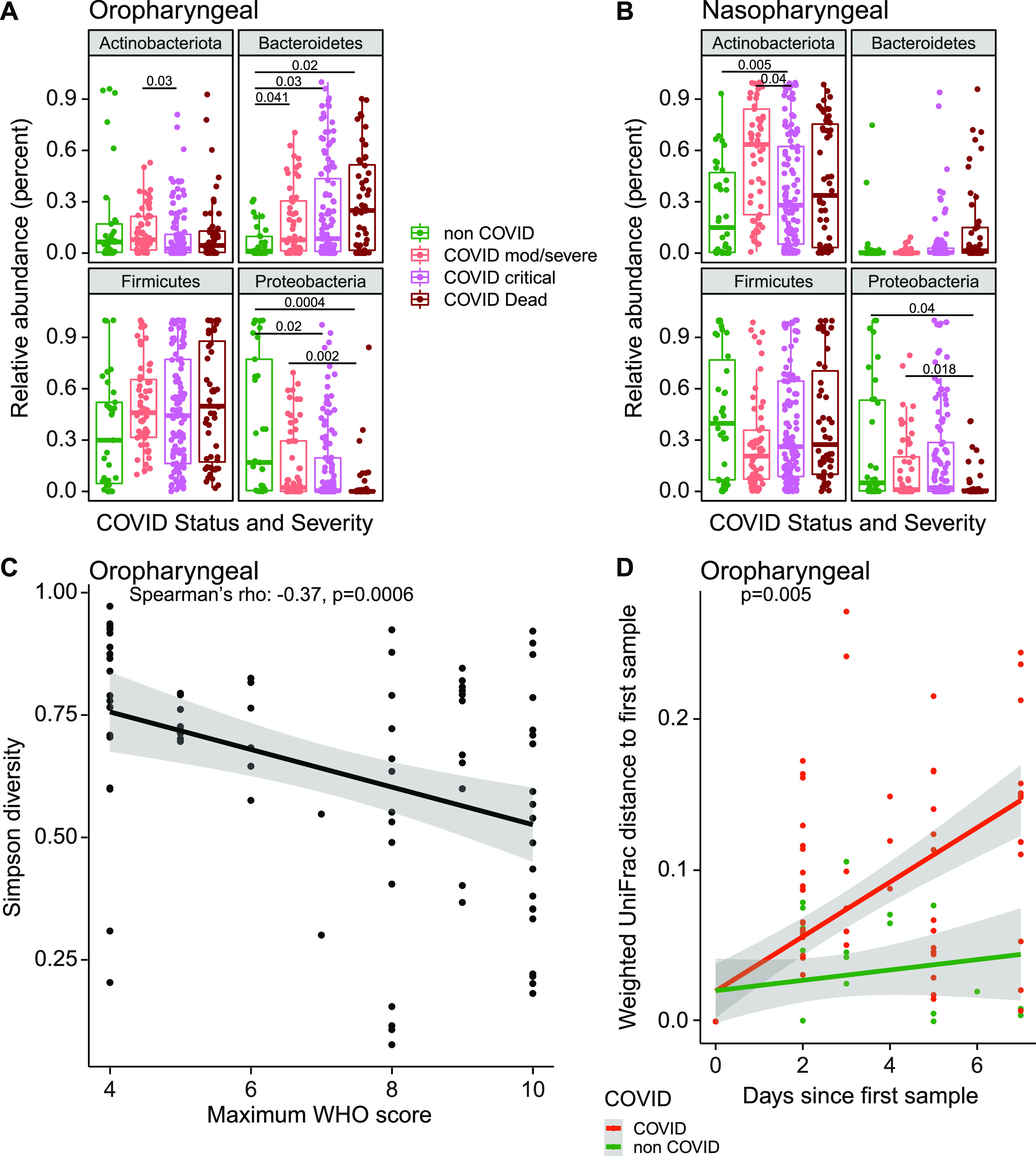
Signatures of disease severity in airway bacterial populations. (A and B) Relative abundances of bacterial phyla, by patient disease status categories. Values for all samples are shown; *P* values were generated using random subsampling for each subject and indicate Wilcoxon pairwise comparisons. (C) Maximum WHO score reached by each patient (*x* axis) versus Simpson diversity index in the first oropharyngeal sample obtained for each subject (*y* axis). The gray shading shows the 95% confidence interval. (D) Divergence in oral bacterial communities over time, comparing COVID-19 (red) and non-COVID (green) samples. The *x* axis shows the time since the first sample; the *y* axis shows the weighted UniFrac distance to the first sample. The gray shading shows the 95% confidence interval.

We compared bacterial phyla that differed between groups, using repeated random subsampling to reduce bias associated with sicker patients being hospitalized longer and having more samples (Fig. 2A and B). Across groups, COVID-19 patients had lower relative abundance of *Proteobacteria* in OP samples than non-COVID patients (FDR = 0.008; Kruskal-Wallis). NP communities showed similar trends but did not meet the FDR threshold for statistical significance. Pairwise between-group comparisons are shown in Fig. 2.

We then assessed the first sampling time point and found that, among COVID-19 patients, decreased numbers of oropharyngeal *Proteobacteria* and *Actinobacteria* correlated with greater WHO score over the course of hospitalization (Spearman’s rho, −0.36 and −0.28, respectively; FDR = 0.008 and 0.05, respectively). At the genus level, patients with more severe disease had significantly lower relative abundances of Haemophilus, *Actinomyces*, and *Neisseria* (FDR < 0.05) (Fig. S3C), all of which are abundant in the normal oropharyngeal microbiome.

Alpha diversity in oropharyngeal samples at the first time point also correlated with COVID-19 severity, with lower diversity being associated with higher WHO score (Spearman’s rho, −0.37; *P* = 0.0006) (Fig. 2C). We then assessed rate of change over time, comparing COVID-19 and non-COVID subjects. Community types were summarized using weighted UniFrac values, which score bacterial abundances, and divergence over time from the subject’s initial sample was calculated (Fig. 2D). The rate of change in oropharyngeal bacterial community structure was significantly greater in COVID-19 than non-COVID subjects (*P* = 0.005, Kruskal-Wallis), indicating that COVID-19 patients experience greater destabilization of bacterial communities during their illness. Significant differences were not seen with unweighted UniFrac, emphasizing that differences were primarily associated with changes in community proportions rather than membership.

### The lung microbiome in intubated COVID-19 patients.

**FIG 3:**
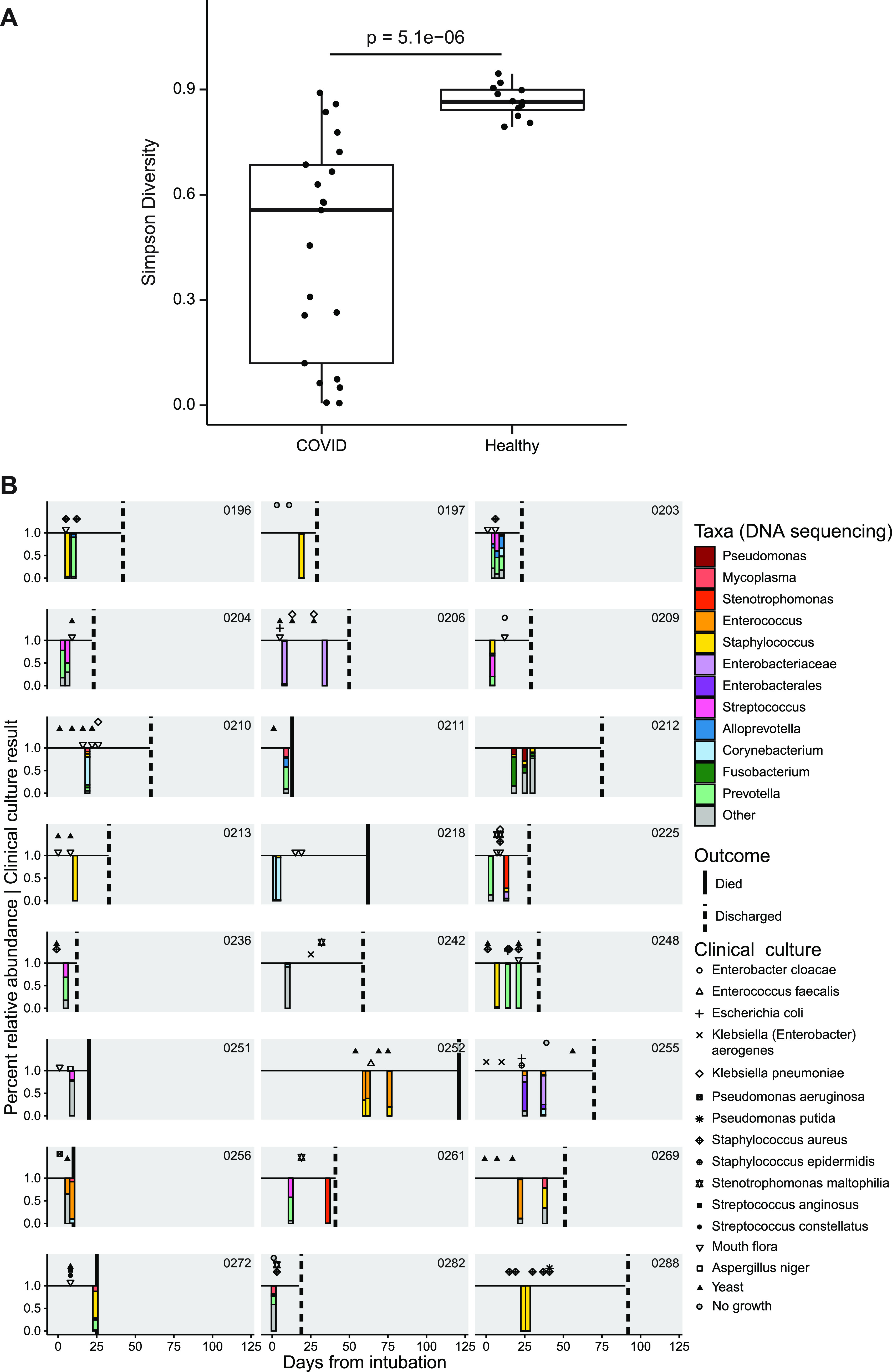
The lower respiratory tract microbiome in intubated COVID-19 patients. (A) Simpson diversity of ETA samples from COVID-19 patients and healthy subjects’ BAL fluid. For COVID-19 patients with multiple samples, only the first ETA sample was used. (B) Timeline of subjects and samples, with results of endotracheal aspirate 16S sequence analysis shown below the line as stacked bar plots (color key to the right) and clinical culture results shown above the line (key to symbols to the right). Taxa are indicated at the lowest taxonomic level assigned by the QIIME/SILVA pipeline, but further identification by BLAST alignment revealed unassigned *Enterobacteriaceae* to be Klebsiella aerogenes and *Enterobacterales* to be Escherichia coli.

The lung microbiome was interrogated in endotracheal aspirate (ETA) samples from 24 intubated subjects (Fig. 3). ETA communities from COVID-19 patients had markedly lower diversity than lung communities from healthy people (Simpson index, 0.56 versus 0.86; *P* = 5.1 × 10^−6^, Wilcoxon rank sum test) (Fig. 3A). There was no relationship between ETA diversity and fatal versus nonfatal outcome (data not shown).

Lineages were heterogeneous and revealed both common respiratory pathogens (Staphylococcus, Klebsiella, and *Stenotrophomonas*) and taxa typical of upper respiratory tract communities (*Corynebacterium* and *Prevotella*). Six of 24 subjects had one or more ETA samples dominated by Staphylococcus (subjects 196, 197, 213, 248, 272, and 288), and another three revealed Staphylococcus as a prominent minority constituent (subjects 209, 252, and 269). Of the 5 subjects with Staphylococcus domination who had respiratory culture within 1 week of sampling, only 3 had Staphylococcus aureus identified by culture, suggesting that either 16S sequencing is more sensitive than culture or dominant Staphylococcus is not S. aureus. Three subjects had ETA samples dominated by *Enterococcus* (subjects 252, 256, and 269). Other respiratory pathogen taxa that dominated smaller numbers of samples (two patients each) included *Stenotrophomonas*, *Enterobacteriaceae* (identified as Klebsiella aerogenes by BLAST), and *Enterobacterales* (identified as Escherichia coli by BLAST).

Among subjects who had serial ETA samples, several showed stable composition over time (subjects 203, 204, 206, 218, 252, and 288), while some demonstrated modest or gradual compositional evolution (subjects 212, 255, and 256). In contrast, several showed marked changes between longitudinal samples (subjects 196, 225, 248, 261, and 269). Thus, the lower respiratory tract microbiome in critically ill intubated COVID-19 patients has low diversity, can be dominated by either pathogens or normal upper respiratory taxa, may have a predilection for Staphylococcus, and can be highly dynamic.

### Commensal DNA viruses are associated with disease severity.

**FIG 4:**
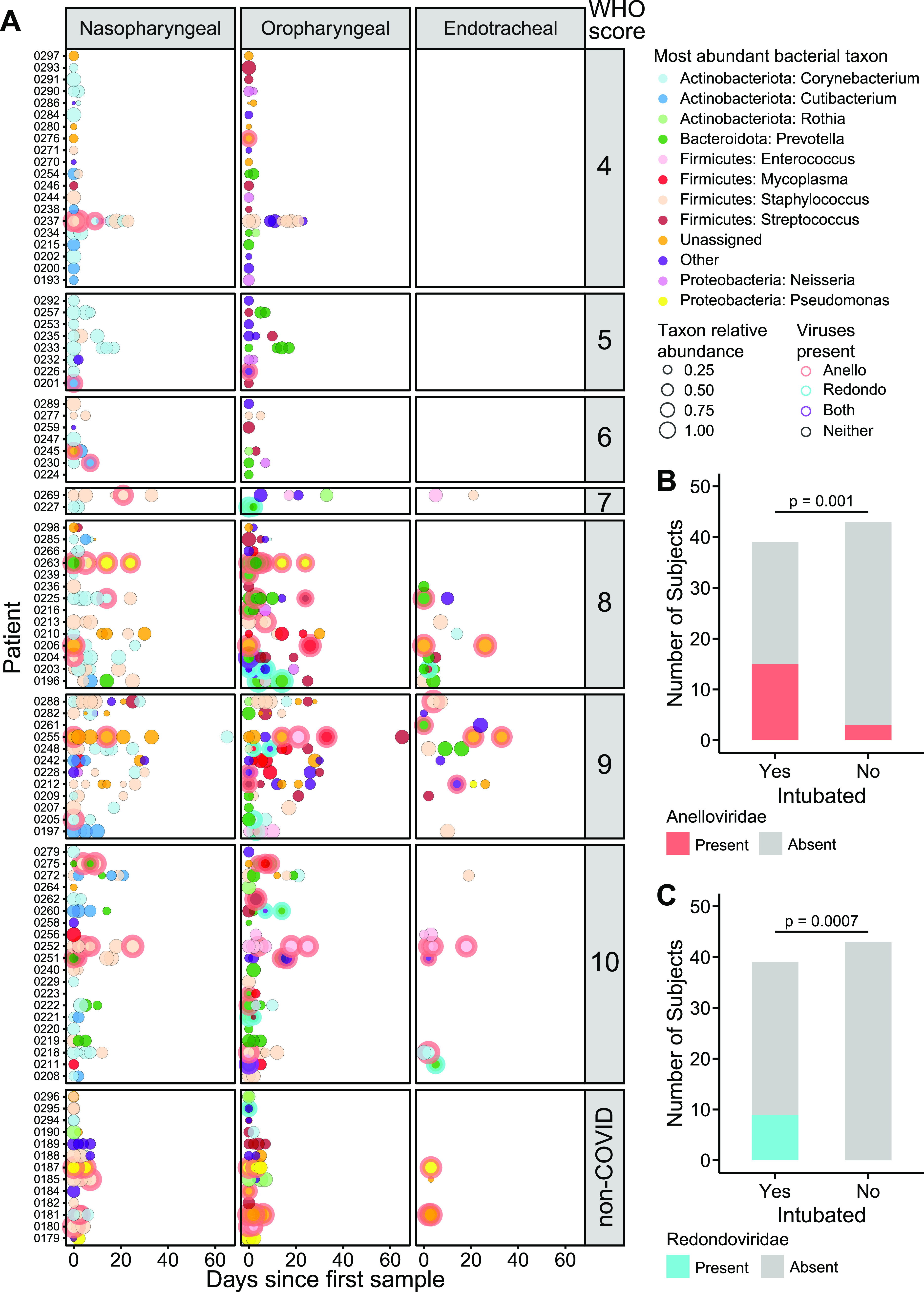
Bacterial dominance and commensal viruses in airway microbial communities. (A) Summary of the most abundant taxa in each subject at different time points in nasopharyngeal, oropharyngeal, and endotracheal communities. The *x* axis shows days since the first sample; each row shows a different patient. Subjects are grouped based on COVID-19 WHO score, with non-COVID patients at the bottom. The types of bacteria are indicated by the color code to the right, and the size of the circle indicates the relative abundance of that dominant bacterial taxon. Detection of *Anelloviridae* and/or *Redondoviridae* is indicated by the ring around some disks and color coded as indicated to the right. (B and C) Detection of *Anelloviridae* and *Redondoviridae* in intubated versus nonintubated patients. To control for longer sampling period in sicker patients, detection in only the first two time point samples were considered.

We next assessed the presence of two airway commensal DNA viruses, *Anelloviridae* and the recently described *Redondoviridae*, using qPCR (Table S3). These viruses are common in the ororespiratory tract and have been associated with respiratory conditions previously (15, 25, 26). Figure 4A shows longitudinal data for *Anelloviridae* and *Redondoviridae* combined with bacterial taxa. At early time points, both *Anelloviridae* and *Redondoviridae* in oropharyngeal samples were positively associated with intubation during hospitalization (Fig. 4B and C; Table S4) (FDR = 0.02 for both; Fisher’s exact test with FDR correction). The commensal DNA viruses were also associated with higher WHO score (Table S4A). Analysis of clinical metadata revealed an association between detection of *Anelloviridae* and *Redondoviridae* in OP samples with administration of hydroxychloroquine, which was in use during much of the sampling period (Table S4A); however, multivariate analysis revealed that the association with treatment was accounted for by the intubation effect. A comparison to clinical laboratory data is shown in Table S4B.

10.1128/mBio.01777-21.7 TABLE S3 Results of qPCR assays to quantify *Anelloviridae* and *Redondoviridae* levels. Download Table S3, PDF file, 0.06 MB. Copyright © 2021 Merenstein et al. 2021 Merenstein et al. https://creativecommons.org/licenses/by/4.0/ This content is distributed under the terms of the Creative Commons Attribution 4.0 International license.

10.1128/mBio.01777-21.8 TABLE S4 Statistical comparison of microbiome data to clinical and immunological data. *P* values are FDR corrected, and significant associations are highlighted. (A) Comparison of microbiome data to patient demographics, treatment, and outcomes. (B) Comparison of microbiome data to clinical laboratory data. (C) Comparison of microbiome data to lymphocyte-to-neutrophil ratios. To account for multiple daily laboratory tests and day-to-day variability, the first value per calendar day was used, and the average of 3 days (day −1, day 0 and day +1 relative to the microbiome sample) was used. (D) Comparison of microbiome data to immune profiling available for 34 subjects. Download Table S4, PDF file, 0.05 MB. Copyright © 2021 Merenstein et al. 2021 Merenstein et al. https://creativecommons.org/licenses/by/4.0/ This content is distributed under the terms of the Creative Commons Attribution 4.0 International license.

### The respiratory microbiome tracks with systemic immune responses.

**FIG 5:**
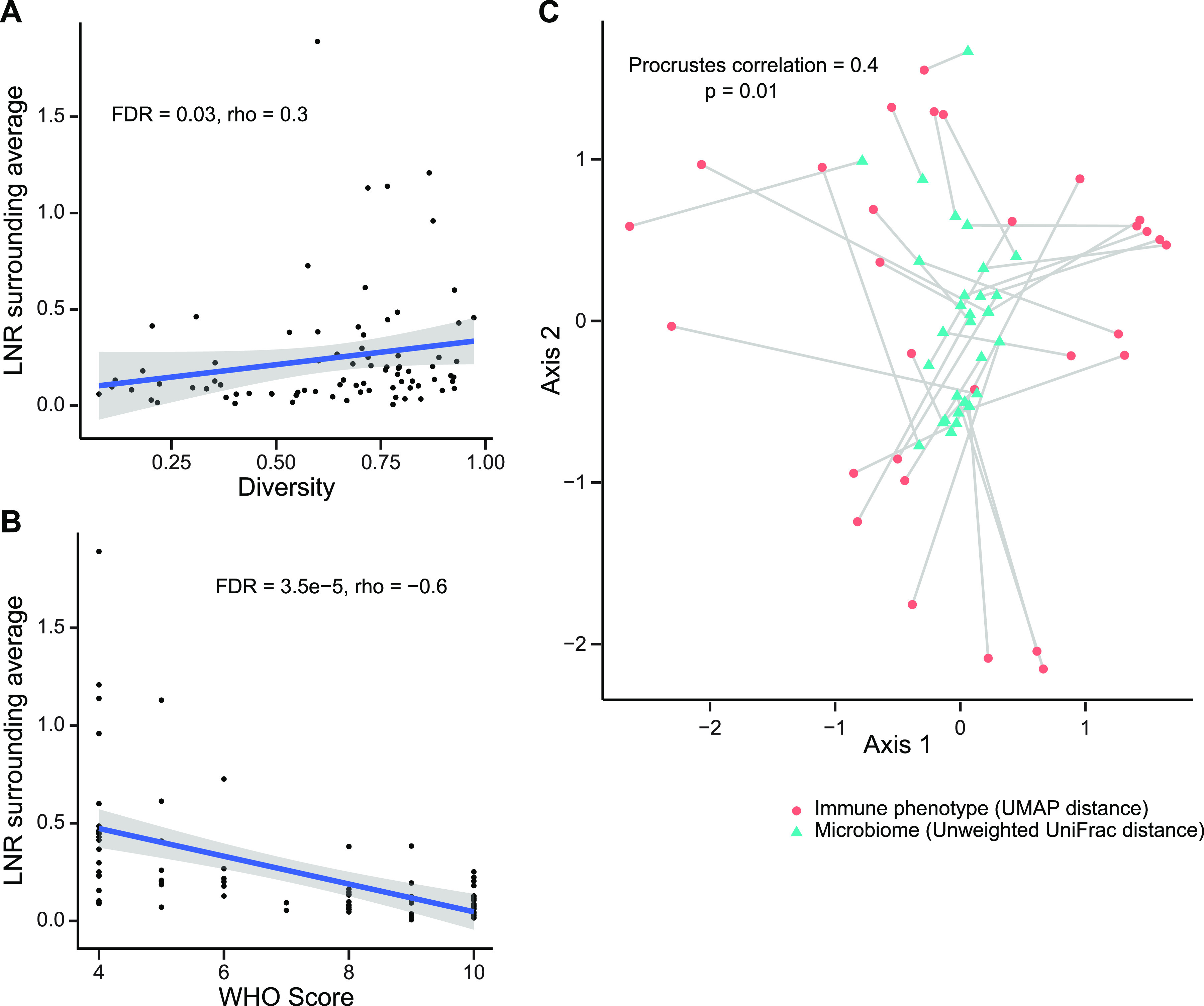
Relationship between oropharyngeal microbiome communities and systemic immune features. (A) Oropharyngeal microbiome diversity at the first time point sampled is plotted against the blood lymphocyte/neutrophil ratio (LNR) at the time of sampling. (B) Blood lymphocyte/neutrophil ratio at time of oropharyngeal sampling (from panel A) is plotted against maximum WHO score during hospitalization. (C) Procrustes analysis in which the UMAP immune profile plot and unweighted UniFrac microbiome plot are overlaid. The immune and microbiome profiles from individual subjects are connected by a line.

We asked whether airway microbiome communities were related to systemic immune or inflammatory features. The ratio of lymphocytes and neutrophils in blood has been associated with COVID-19 severity and outcomes (27, 28). We found that lower lymphocyte-to-neutrophil ratio (LNR) was associated with both lower diversity (FDR = 0.03; *r* = 0.23, Spearman correlation) (Fig. 5A) and composition of the oropharyngeal microbiome (UniFrac second principal coordinate: FDR = 0.01, *r* = 0.32 [weighted]; FDR = 0.008, *r* = 0.35 [unweighted]; Spearman correlation) (Table S4C). As expected, LNR correlated inversely with disease severity (FDR = 3.5 × 10^−5^, *r* = −0.6, Spearman correlation) (Fig. 5B). The slope of LNR change over time also correlated inversely with WHO score (Spearman rho = −0.43; *P* = 5.37 × 10^−5^) but was not associated with OP microbiome diversity or change in diversity over time.

We then investigated peripheral blood mononuclear cell (PBMC) phenotyping, which was available for 34 of the subjects (Table S1) coenrolled in a deep immune profiling study of COVID-19 patients (29). That study assessed 193 individual cellular immune features and integrated them in a high-dimensional immune phenotype analysis (uniform manifold approximation and projection [UMAP]) that reduced the immune features to a two-dimensional landscape and created compacted metafeatures reflected in the two components.

We initially tested a limited set of individual B cell (B cells and plasmablasts) and T cell (CD4 and CD8 total and Ki67^+^; HLA-DR/CD38^+^; CD4Tfh; CD8Temra) populations and found no association with the microbiome that reached significance after FDR correction (Table S4D). In order to encompass the multiple immune parameters, we then compared global immune patterns represented by the UMAP distance matrix (generated from the 193 immune components) to the overall respiratory microbiome profiles represented by the unweighted UniFrac distance matrix. This revealed a significant correlation between the OP microbiome distance matrix and the systemic immune profile distance matrix (*P* < 0.001; Mantel's test). We also applied a Procrustes analysis, which provides visualization of the overlay of the two multidimensional matrices (Fig. 5C) and which also revealed a significant correlation (Procrustes correlation, 0.4; *P* = 0.01). Thus, both analytic approaches show that the oropharyngeal microbiome composition is globally correlated with systemic immune cell composition.

### Machine learning identifies signatures associated with COVID-19 severity.

**FIG 6:**
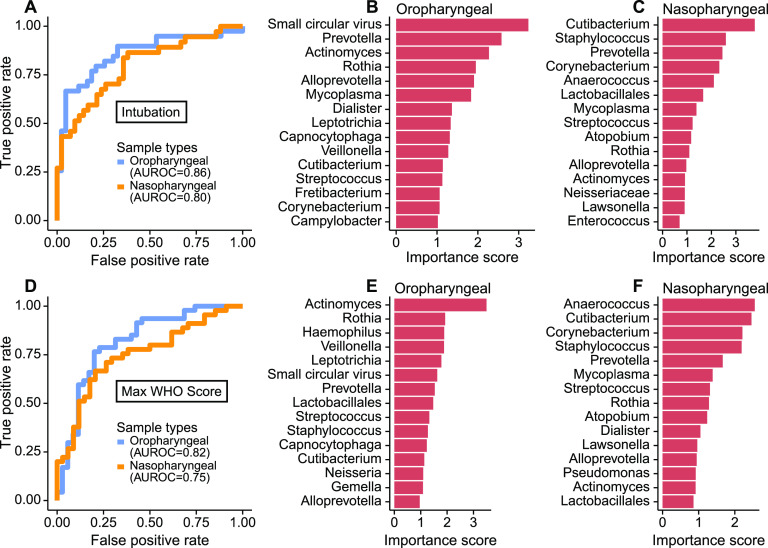
Random forest classification to detect signatures of severity in the SARS-CoV-2-infected subjects. (A) Receiver operating characteristic (ROC) curve of random forest classification on patient intubation status using OP and NP samples. The top 15 most important predictors in classifying patient’s intubation status using OP (B) and NP (C) samples are shown. (D) ROC curve of random forest classification on disease severity using both OP and NP samples. The top 15 most important predictors in classifying disease severity using OP (E) and NP (F) samples are shown.

Last, we sought to identify microbiome features most associated with disease severity, employing the random forest machine learning algorithm. This analysis incorporated abundances of bacterial taxa, bacterial community features, and commensal DNA viruses in OP or NP samples to discriminate patients who needed intubation and WHO score (Fig. 6). We used the first two samples for each patient to allow more homogenous comparison between patients sampled for different durations.

Both NP and OP microbiome data discriminated between patients requiring intubation or not (area under the receiver operating characteristics [AUROC] = 0.80 and 0.86, respectively) (Fig. 6A). The feature that contributed most to clinical status discrimination was the presence of small circular DNA viruses for OP samples, with higher levels of the viruses associated with intubation (Fig. 6B). Also positively associated with intubation were *Prevotella* and *Mycoplasma*. Lower random forest accuracy rates were observed in NP than OP samples, but more than 50% of the most important taxa were present in both sample types (Fig. 6C). OP and NP data also discriminated disease severity (WHO score of 4 to 6 versus 7 to 10; AUROC = 0.82 and 0.75, respectively) (Fig. 6D). Bacterial lineages rather than the DNA viruses showed the strongest discriminatory power for WHO score (Fig. 6E and F). Thus, these data define signatures of microbial activity associated with intubation and COVID-19 severity.

## DISCUSSION

We found marked disruption of the oropharyngeal microbiome in hospitalized COVID-19 patients, which differed from that of non-COVID patients and was associated with disease severity. COVID-19 patients also showed greater destabilization of the microbiome over time than did non-COVID patients. Endotracheal samples in intubated COVID-19 patients had low diversity and revealed frequent outgrowth of potential respiratory pathogens, particularly Staphylococcus. Oropharyngeal microbiome communities were associated with blood leukocyte populations and global PBMC immune profiles. Small DNA commensal viruses of the respiratory tract also differed among COVID-19 patients based on whether intubation was required. Together, the combination of bacterial and viral features at early time points had high classifier accuracy in distinguishing intubated versus nonintubated patients and clinical status reached over the course of hospitalization.

Several possibilities could account for the association between the oropharyngeal microbiome and COVID-19 severity. Dysbiosis of the vaginal microbiome and consequent inflammation are strongly associated with sexual acquisition of HIV infection (30). In the respiratory tract, the local microbiome appears to regulate mucosal immune tone (31), and local respiratory tract inflammation affects susceptibility to respiratory syncytial virus (RSV) infection and disease severity (9, 10). ACE2, the receptor for SARS-CoV-2, is an interferon-stimulated gene (32) and thus could be modulated by the respiratory microbiome. While we did not have measures of local lung inflammation or immunity, we found that cellular immune profiles in blood correlated with oropharyngeal microbial communities in patients for whom both were available. Furthermore, the lung microbiome derives largely from that in the oropharynx (18) and could also be affected by OP microbiome profiles. It would be useful for future studies to investigate whether distinct respiratory microbiome profiles play a role in regulating SARS-CoV-2 infection or host response, including the propensity for infection to propagate from the upper to lower respiratory tract. It would also be important to determine whether respiratory tract dysbiosis is characteristic of the types of comorbidities that are associated with increased risk of severe COVID-19 (which were present in nearly all of our subjects) and, if so, whether it may mediate some of that increased risk.

Other mechanisms could potentially link the microbiome and COVID-19 disease. Commensal bacteria with heparinase activity are reported to alter SARS-CoV-2 binding to target cells (33), but we found no differences in predicted heparinase activity based on imputed bacterial metagenomes between COVID-19 and non-COVID patients or among those with different disease severities (data not shown). Conversely, SARS-CoV-2 infection might itself alter the local microbiome through inflammation or other mechanisms. It will be important to distinguish these pathways and determine whether interventions to modify the microbiome could prevent infection or diminish disease severity.

Our longitudinal analysis revealed greater destabilization of the oropharyngeal bacterial microbiome in COVID-19 than non-COVID patients. This finding is striking given that non-COVID patients were overall sicker (all in the intensive care unit [ICU]; 40% mortality) than COVID-19 patients (both ICU and non-ICU patients; 24% mortality) and had similar length of stay (mean, 24.8 ± 13.3 versus 23.3 ± 23.3 days). While use of antibacterial drugs could differentially affect the microbiome, both COVID-19 and non-COVID patients received extensive antibiotic treatment (Fig. S1). Other interventions that might impact the microbiome, such as intubation, were also not more frequent in COVID-19 patients. This observation is consistent with the possibility that SARS-CoV-2 infection of the respiratory mucosa might itself also drive changes in microbiome communities.

The small commensal DNA viruses *Anelloviridae* and *Redondoviridae* were the top-ranking microbiome features in OP samples for distinguishing patients who required intubation. Prevalence in human adult populations ranges from 67 to 100% for *Anelloviridae*, which are found in blood and many tissues, including the respiratory tract. *Anelloviridae* levels are typically elevated in immunocompromised states (13, 14). *Redondoviridae* are a recently described family of viruses that appear restricted to the human oral and respiratory tract and have a prevalence of up to 15% (15, 34). *Redondoviridae* levels are elevated in the airway of intubated patients and in oral samples of periodontitis patients (15, 34), suggesting that they may be barometers for disease activity in some conditions. While small circular DNA viruses were the most powerful discriminators of intubation (Fig. 6B), they were not as strong as bacterial composition in predicting overall clinical severity (Fig. 6E). Since some subjects reached higher WHO scoring without intubation (e.g., due to vasopressor use and/or renal failure), this result suggests that these viruses may be specifically associated with intubation. Such a relationship might be related to mucosal disruption or local inflammation that could be associated with intubation.

Our observation that integrated features of the upper respiratory tract microbiome and commensal viruses correlate with COVID-19 severity and outcome is complementary to a study of intubated COVID-19 patients which reported that mortality correlated with integrated features of lower respiratory tract microbiome, SARS-CoV-2 RNA levels, antibody levels, and transcriptome patterns (35). Thus, while the individual parameters investigated differed in these two studies, together they suggest that the entire airway, including both the upper and lower respiratory tract, is associated with COVID-19 outcomes. Considerable focus has been placed on identifying patients at high risk of poor outcomes who might benefit from novel or more intensive treatments, and it would be useful to test whether incorporating oropharyngeal microbiome data would enhance clinical management algorithms.

Bacterial superinfection in COVID-19 is an area of great interest and importance (36–38). Our lung microbiome analysis revealed complex and patient-specific patterns that were often dynamic. High relative abundance was seen for several anticipated pathogens, notably Staphylococcus (9/24 subjects). This molecular profiling of the lung microbiome is concordant with culture-based studies highlighting Staphylococcus aureus as an emerging copathogen in COVID-19 (39–41). Bacterial superinfection with Staphylococcus is a long-recognized consequence of influenza and an important cause of morbidity and mortality (6, 7), so further studies will be useful to determine if similar mechanisms operate during COVID-19. We also noted several patients with high relative abundances of *Enterococcus*, which was recently described as a common cause of bloodstream infection in critically ill COVID-19 patients (42).

Microbiome communities were associated with systemic immune profiles. The ratio of lymphocytes to neutrophils is a marker of COVID-19 severity and outcomes (27, 28) and is correlated with oropharyngeal microbiome diversity and composition. The overall structure of the oropharyngeal microbiome was also correlated with global cellular immune profiles in subjects for whom deep cellular immune profiling was available. These immune profiles are also associated with COVID-19 severity (29). It is unclear whether the respiratory tract microbiome directs systemic inflammatory responses, whether inflammatory response shapes the microbiome, or whether both are responsive to other factors associated with disease severity.

Our study has several limitations. Many patients presented with severe illness and when initially sampled were already intubated or had reached high WHO scores. Thus, the ability to distinguish clinical course based on microbiome markers may not necessarily reflect prior predictive power. Our patients were heterogenous, with extensive use of antibiotics, which could influence the bacterial microbiome, and many had already received antimicrobial therapy at time of initial sampling. Thus, future studies should address stratification for antibiotic use as well as baseline disease severity. Subjects were enrolled early in the COVID-19 pandemic, when clinical management and outcomes may not have been optimal. We did not analyze local lung mucosal immune/inflammatory features, and systemic immune profiling was available for only a subset of patients, both of which could potentially influence the microbiome. We focused on two specific commensal DNA viruses rather than the comprehensive airway virome. There is no gold standard for diagnosis of bacterial pneumonia superinfection in this population, limiting the ability to definitively link ETA findings, and lower respiratory tract microbiome information was available only for the patients who were intubated. Finally, while we enrolled a large number of COVID-19 patients, the number of non-COVID subjects was modest.

In summary, we report profound dysbiosis of the respiratory tract bacterial and viral microbiome in hospitalized COVID-19 patients, which differs from that of non-COVID patients, exhibits accelerated destabilization over time, and associates with disease severity and systemic immune profiles. In intubated patients the lung microbiome is dysbiotic with frequent enrichment of Staphylococcus. The small commensal viruses *Anelloviridae* and *Redondoviridae* were the strongest discriminators of patient intubation. This work provides a basis for further studies to delineate mechanisms linking the respiratory tract microbiome and outcomes and provides potential biomarkers to assess and/or predict clinical course that should be validated in independent cohorts.

## MATERIALS AND METHODS

### Subjects.

Following informed consent (IRB protocol 823392), samples were collected beginning a median of 4 days after hospitalization (generally within 1 week of hospitalization or identification of COVID-positive status if postadmission). Oropharyngeal (OP) and nasopharyngeal (NP) swabs, and endotracheal aspirates (ETA) from intubated subjects, were obtained as previously described (18). Additional OP and NP swabs were obtained and eluted in viral transport medium (VTM) for SARS-CoV-2 analysis as previously described (20). COVID-19 patients were classified clinically by maximum score reached during hospitalization using the 11-point WHO COVID-19 progression scale (16). Non-COVID subjects were patients hospitalized in the intensive care unit (ICU) with a variety of underlying disorders. Healthy controls included 30 individuals who underwent OP and NP sampling and 12 subjects who had undergone bronchoscopy and bronchoalveolar lavage (BAL) previously, as described elsewhere (17–19).

### 16S rRNA gene sequencing and analysis.

DNA extraction, 16S rRNA gene PCR amplification using V1V2 primers (Table S5), and Illumina sequencing were carried out as described elsewhere (23, 24). NP, OP, and BAL sample 16S rRNA gene V1V2 sequences from healthy controls were acquired previously using the Roche 454 GS-FLX platform (17–19), which we showed yields comparable results to and can be integrated with Illumina data (18). Processing using the QIIME2 pipeline, calculations of alpha diversity, UniFrac distances, principal coordinate analysis (PCoA), and permutational multivariate analysis of variance (PERMANOVA) testing are detailed in the supplemental methods. For analyses comparing groups with different numbers of samples per subject, PERMANOVA testing used specimens randomly subsampled 1,000 times to one sample per patient, and mean *P* values are reported.

10.1128/mBio.01777-21.9 TABLE S5 Synthetic oligonucleotides used in this study. Download Table S5, PDF file, 0.01 MB. Copyright © 2021 Merenstein et al. 2021 Merenstein et al. https://creativecommons.org/licenses/by/4.0/ This content is distributed under the terms of the Creative Commons Attribution 4.0 International license.

### Viral analysis.

Extracted DNA was amplified using Phi29 DNA polymerase and random hexamers and then subjected to qPCR using primers/probes that target *Redondoviridae* (RV) and *Anelloviridae* (Table S5) as described elsewhere (15, 43). Levels of SARS-CoV-2 RNA were quantified in total RNA extracted from ETA samples or VTM, and complete SARS-CoV-2 genome sequences were generated as recently reported (20).

### Clinical and immune data.

Flow cytometric cellular immune profiling of PBMCs was available on a subset of subjects as described elsewhere (29). The unbiased uniform manifold approximation and projection (UMAP) approach was used to distill 193 individual immune components into two principal components (29). The microbiome unweighted UniFrac PCoA was compared with blood cellular UMAP analysis using Mantel’s test and Procrustes analysis.

### Statistical analysis.

Nonparametric tests were used to compare two independent groups (Wilcoxon rank sum test), two related groups (Wilcoxon signed-rank test), and multiple groups (Kruskal-Wallis test). Spearman’s rank order correlation was used for nonparametric correlation analysis. Fisher’s exact tests were used to test differences between two categorical variables. *P* values are from two-sided comparisons. *P* values for multiple comparisons were corrected using the Benjamini-Hochberg FDR method. Random forest classification was implemented using the randomForest package (v4.6-14) in R. Decision trees were trained on data consisting of bacterial relative abundance at the genus level and small circular DNA virus copy numbers (*Redondoviridae* or *Anelloviridae*) in samples from the first two time points, as detailed in the supplemental methods.

### Data availability.

Sample information and raw sequences analyzed in this study are available in the National Center for Biotechnology Information Sequence Read Archive under accession BioProject accession IDs PRJNA678105 and PRJNA683617 (details listed in Table S6). Computer code used in this study is available at https://github.com/BushmanLab/covid_microbiome_2021.

10.1128/mBio.01777-21.10 TABLE S6 Accession numbers of sequence data generated in this study. Download Table S6, PDF file, 0.06 MB. Copyright © 2021 Merenstein et al. 2021 Merenstein et al. https://creativecommons.org/licenses/by/4.0/ This content is distributed under the terms of the Creative Commons Attribution 4.0 International license.

10.1128/mBio.01777-21.1 TEXT S1 Supplemental methods. Download Text S1, PDF file, 0.07 MB. Copyright © 2021 Merenstein et al. 2021 Merenstein et al. https://creativecommons.org/licenses/by/4.0/ This content is distributed under the terms of the Creative Commons Attribution 4.0 International license.
