# Correction for Merenstein et al., “Signatures of COVID-19 Severity and Immune Response in the Respiratory Tract Microbiome”

**DOI:** 10.1128/mbio.02293-22

**Published:** 2022-08-31

**Authors:** Carter Merenstein, Guanxiang Liang, Samantha A. Whiteside, Ana G. Cobián-Güemes, Madeline S. Merlino, Louis J. Taylor, Abigail Glascock, Kyle Bittinger, Ceylan Tanes, Jevon Graham-Wooten, Layla A. Khatib, Ayannah S. Fitzgerald, Shantan Reddy, Amy E. Baxter, Josephine R. Giles, Derek A. Oldridge, Nuala J. Meyer, E. John Wherry, John E. McGinniss, Frederic D. Bushman, Ronald G. Collman

## AUTHOR CORRECTION

Volume 12, no. 4, e01777-21. https://doi.org/10.1128/mBio.01777-21. In Table S5 in the supplemental material, two primer sequences were listed incorrectly. The actual sequence of TTV_3656_Fwd is AGACTCCGAGDTGCYMTTGG, and the actual sequence of TTV_3656_Rev is CTYAAAATGGCBGCYGTGAC.

10.1128/mbio.02293-22.1 TABLE S5 Synthetic oligonucleotides used in this study. Download Table S5, XLSX file, 0.01 MB. Copyright © 2022 Merenstein et al. 2022 Merenstein et al. https://creativecommons.org/licenses/by/4.0/ This content is distributed under the terms of the Creative Commons Attribution 4.0 International license.

