# Supplementary figures and images for: Signatures of COVID-19 Severity and Immune Response in the Respiratory Tract Microbiome
Source: mBio. 2021 Aug 17;12(4):e01777-21. doi: 10.1128/mBio.01777-21 (PMC8406335; doi:10.1128/mBio.01777-21)

Figure S1

A

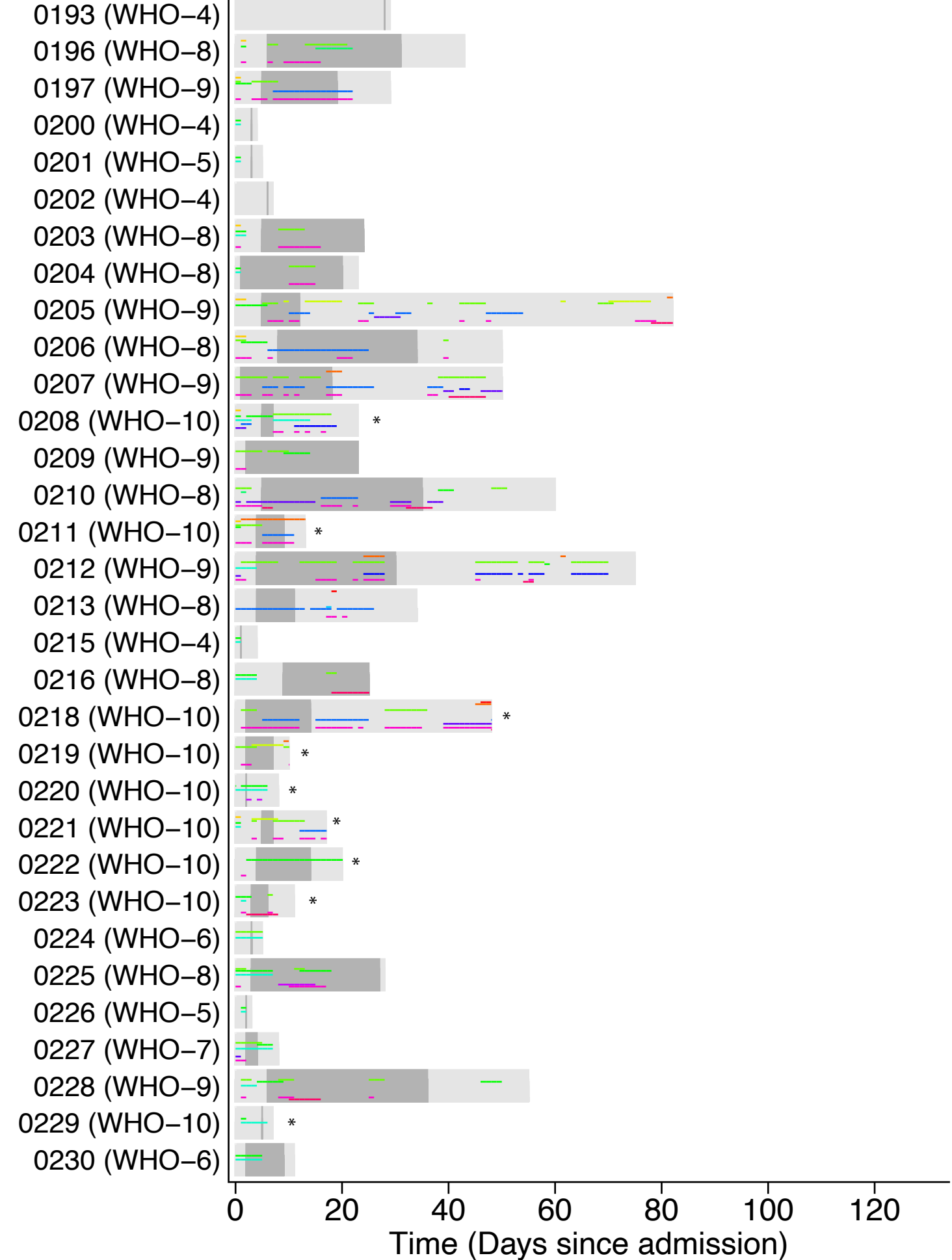

B

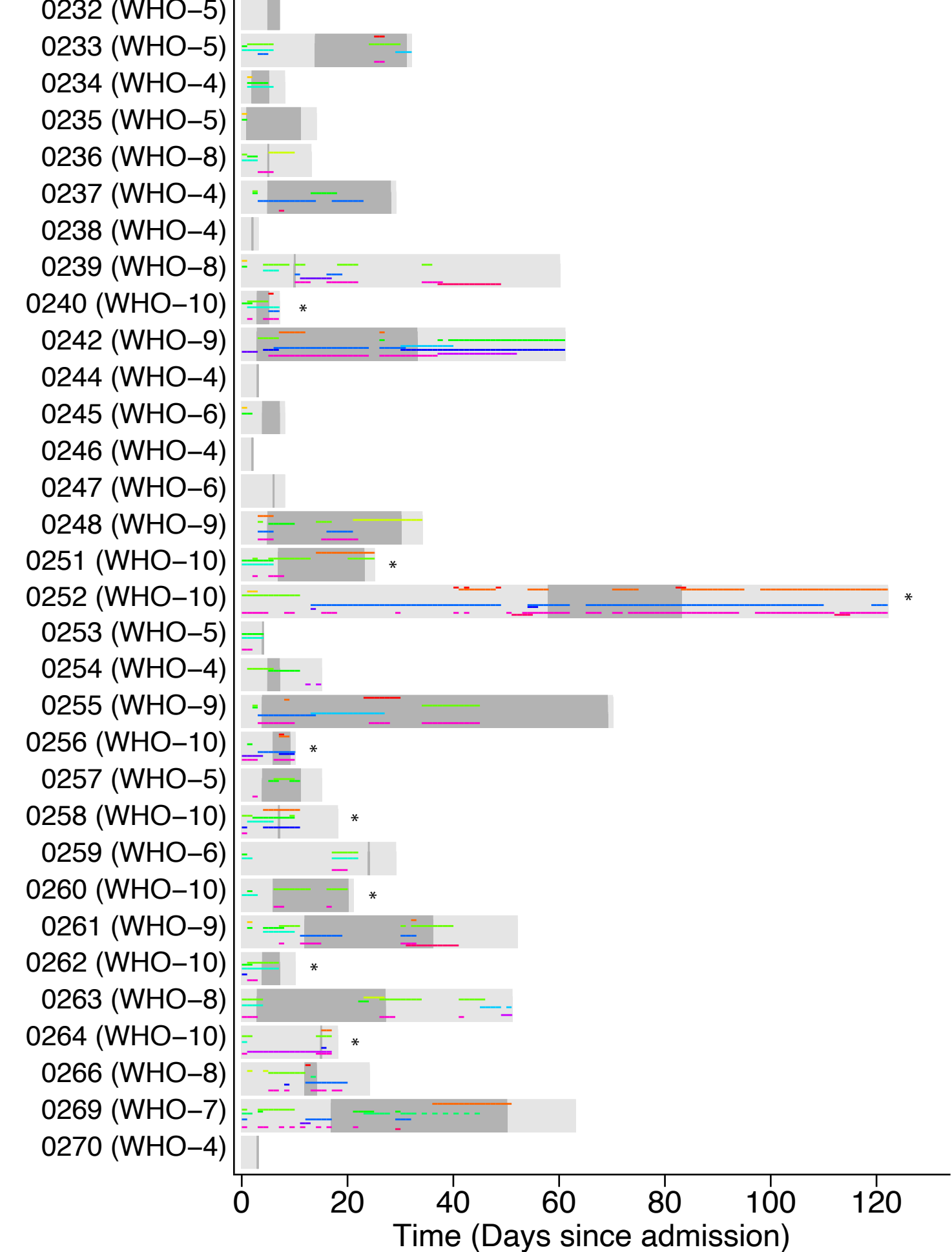

C

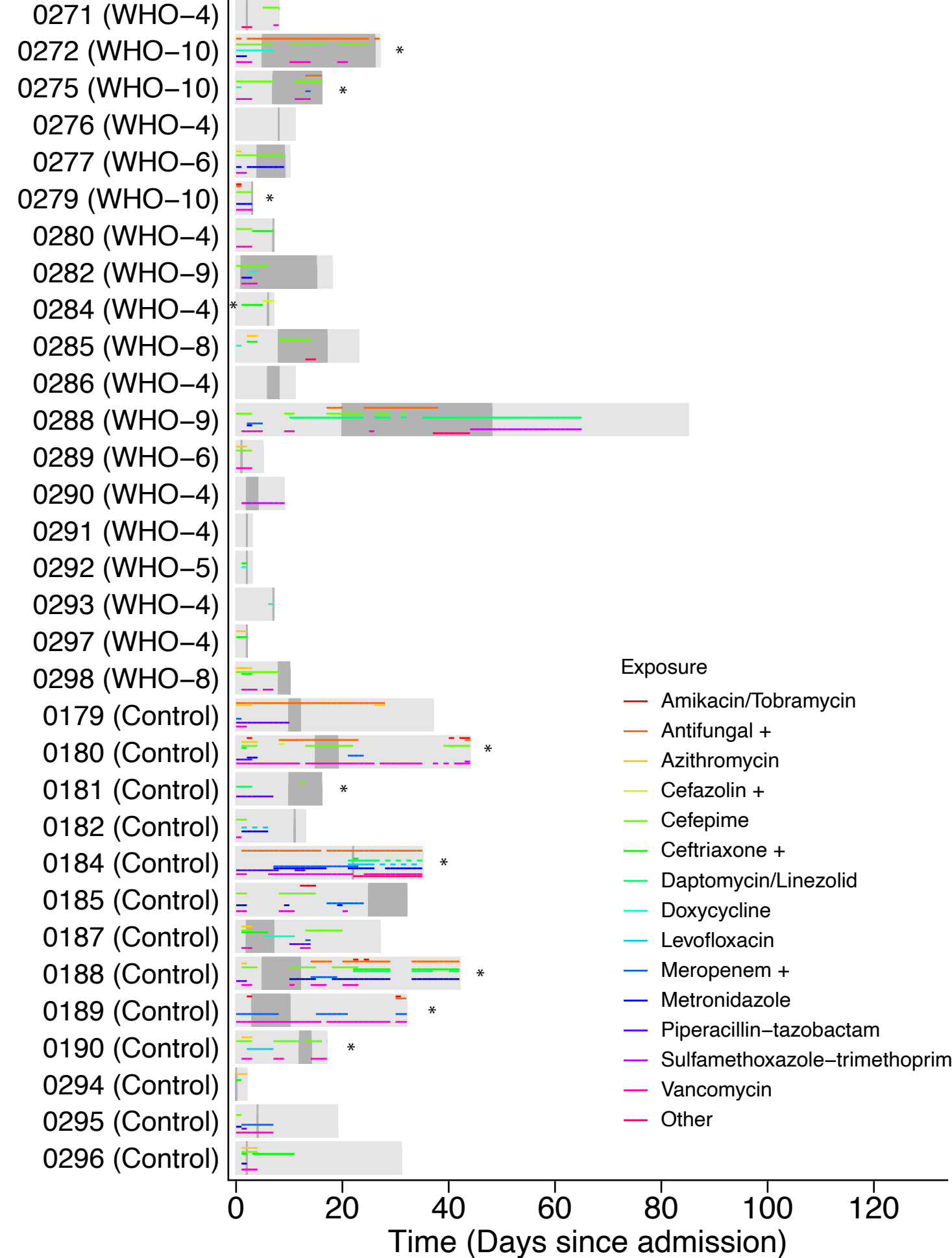

Supplement: FIG S1 [file mbio.01777-21-sf001.pdf]

**Figure S2**

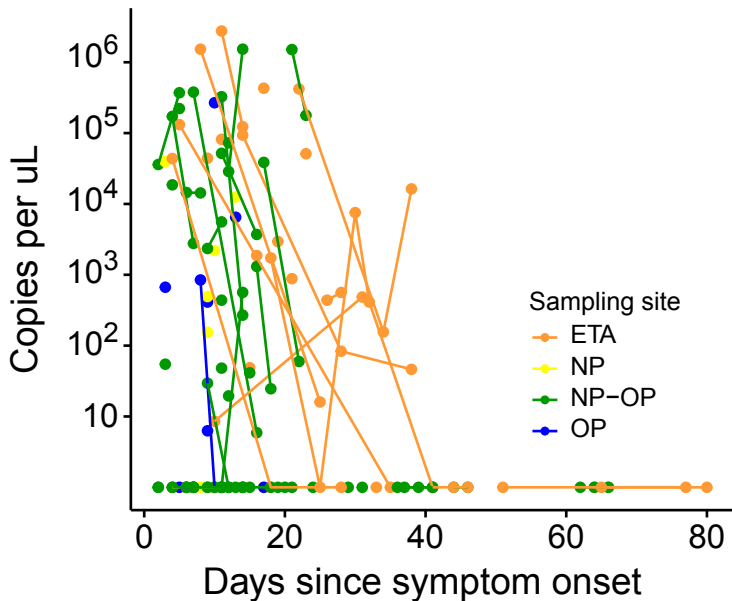

Supplement: FIG S2 [file mbio.01777-21-sf002.pdf]

**Figure S3****A**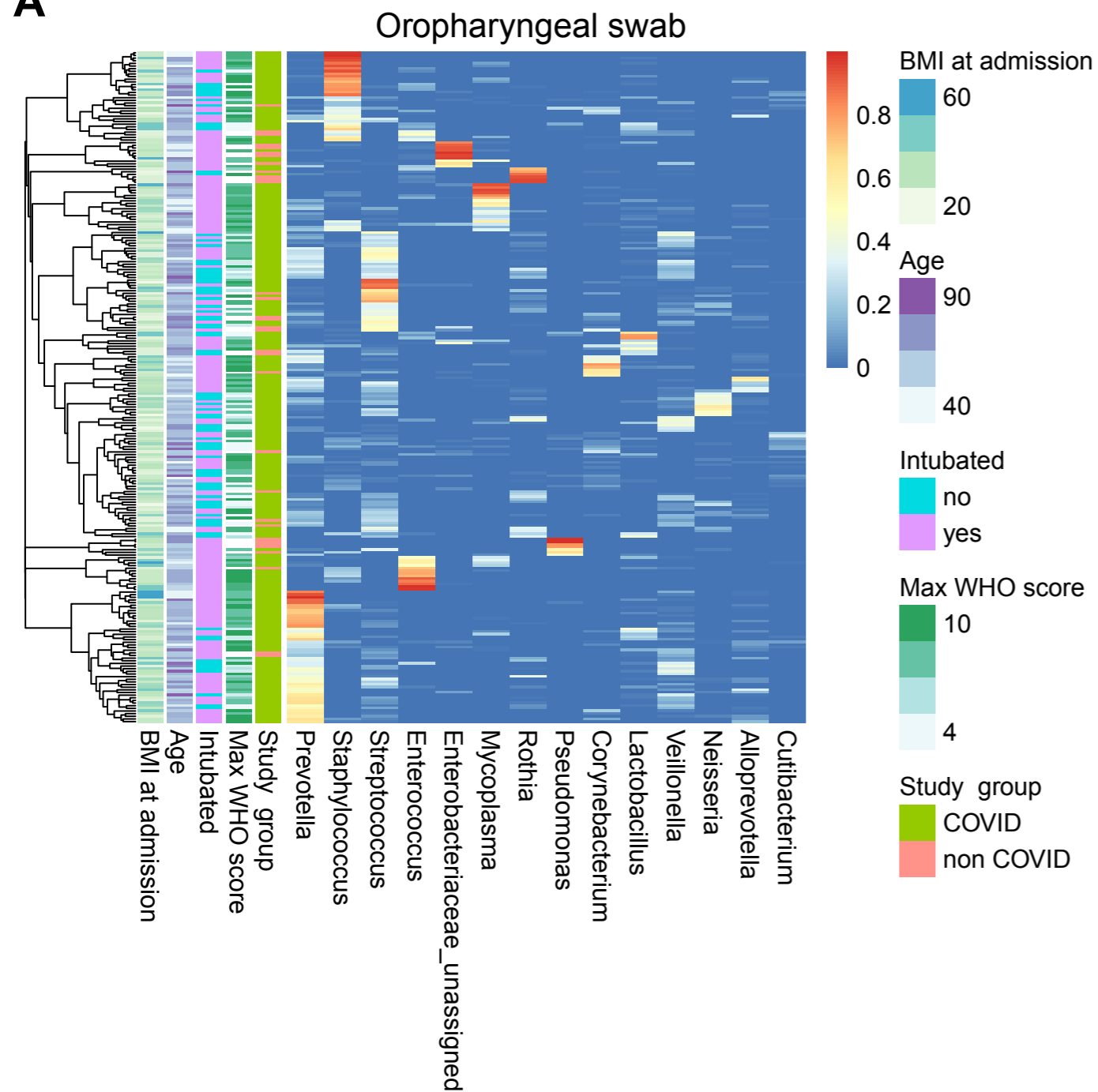**B**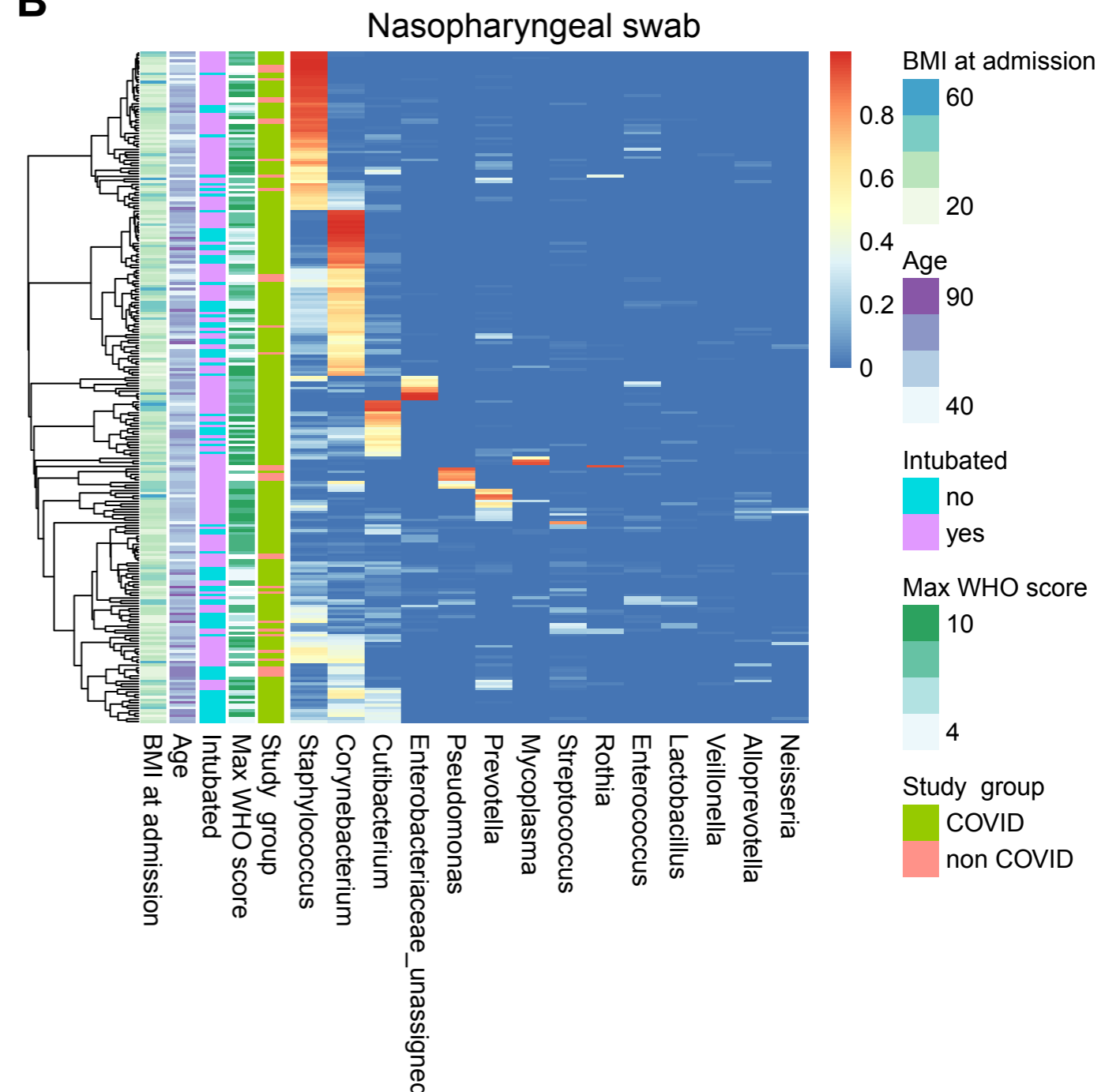**C**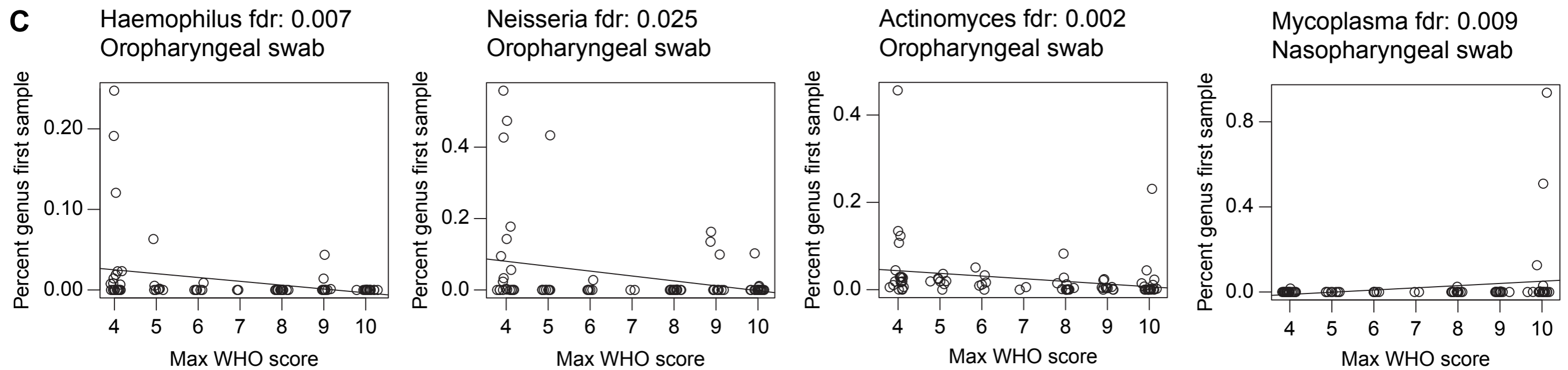

Supplement: FIG S3 [file mbio.01777-21-sf003.pdf]
