# Supplementary material for: Signatures of COVID-19 Severity and Immune Response in the Respiratory Tract Microbiome
Source: mBio. 2021 Aug 17;12(4):e01777-21. doi: 10.1128/mBio.01777-21 (PMC8406335; doi:10.1128/mBio.01777-21)
Supplement: TABLE S1 [file mbio.01777-21-st001.pdf]

Table S1. Demographic and clinical information on subjects.

		Diagnosis and outcome						COVID-19 treatment				Preexisting comorbidities															
Subject ID	Primary diagnosis	+ COVID test (days from symtoms)	Hospitali-zation (days from symptoms)	Intubation	Max WHO score	Outcome	Day of live discharge or death	Remdesivir	Cortico-steroids	Hydroxy-chloroquine	Convale-scent plasma	BMI	Diabetes	Hyper-tension	Coronary artery disease	Stroke	Other cardio-vascular	Asthma	COPD	Other chronic lung	Renal (stage ≥4)	HIV infection	Cancer (within 6 months)	Organ transplant	Chemo or immune suppression (within 3 months)	Any major comorbidity	PBMC Immune Profiling
CORE-0193	COVID	6	-3	No	4	Live	29	No	No	Yes	No	24	Yes	Yes	No	Yes	Yes	No	No	Yes	No	Yes	No	No	No	Yes	
CORE-0196	COVID	7	6	Yes	8	Live	43	Yes	Yes	Yes	No	62	No	Yes	No	No	Yes	No	No	Yes	No	No	No	No	No	Yes	
CORE-0197	COVID	5	4	Yes	9	Live	29	Yes	No	Yes	No	48	Yes	Yes	No	No	No	No	No	Yes	No	No	No	No	No	Yes	
CORE-0200	COVID	3	3	No	4	Live	4	No	Yes	Yes	No	25	No	Yes	No	No	No	No	No	No	No	No	No	No	No	Yes	
CORE-0201	COVID	6	9	No	5	Live	5	Yes	No	Yes	No	37	Yes	Yes	No	No	No	No	No	No	No	No	No	No	No	Yes	
CORE-0202	COVID	8	7	No	4	Live	7	No	No	Yes	No	27	Yes	Yes	No	No	No	No	No	No	Yes	No	No	Yes	Yes	Yes	
CORE-0203	COVID	3	7	Yes	8	Live	24	No	Yes	Yes	No	31	No	No	No	No	No	No	No	No	No	No	No	No	No	No	
CORE-0204	COVID	7	7	Yes	8	Live	23	No	No	Yes	No	22	No	No	No	No	No	No	No	No	No	No	No	No	No	No	
CORE-0205	COVID	6	6	Yes	9	Live	82	No	No	Yes	No	37	Yes	Yes	No	Yes	No	No	No	No	No	No	No	No	No	Yes	Yes
CORE-0206	COVID	4	4	Yes	8	Live	50	No	Yes	Yes	No	57	Yes	Yes	Yes	No	Yes	No	Yes	Yes	No	No	No	No	No	Yes	
CORE-0207	COVID	4	4	Yes	9	Live	50	Yes	Yes	Yes	No	49	Yes	Yes	Yes	No	No	Yes	No	Yes	No	No	No	No	No	Yes	Yes
CORE-0208	COVID	7	7	No	10	Dead	23	No	No	Yes	No	28	Yes	Yes	No	No	Yes	No	Yes	No	Yes	No	No	Yes	Yes	Yes	
CORE-0209	COVID	5	13	Yes	9	Live	23	No	No	Yes	No	33	No	Yes	Yes	No	No	No	No	Yes	No	No	Yes	No	No	Yes	Yes
CORE-0210	COVID	0	0	Yes	8	Live	60	No	Yes	Yes	No	24	Yes	Yes	No	No	Yes	No	No	No	No	No	No	No	No	Yes	Yes
CORE-0211	COVID	8	8	Yes	10	Dead	13	No	Yes	Yes	No	34	No	Yes	No	No	No	No	No	No	No	No	Yes	No	Yes	Yes	Yes
CORE-0212	COVID	3	3	Yes	9	Live	75	No	Yes	Yes	No	33	Yes	Yes	No	No	Yes	No	Yes	No	Yes	No	No	No	No	Yes	
CORE-0213	COVID	1	1	Yes	8	Live	34	No	Yes	Yes	No	26	Yes	Yes	No	Yes	Yes	No	No	No	Yes	No	No	No	No	Yes	
CORE-0215	COVID	12	12	No	4	Live	4	No	No	Yes	No	39	No	Yes	No	No	No	No	No	No	No	No	No	No	No	Yes	Yes
CORE-0216	COVID	3	3	Yes	8	Live	25	Yes	Yes	Yes	No	37	Yes	Yes	No	No	No	No	Yes	Yes	No	No	No	No	No	Yes	
CORE-0218	COVID	3	24	Yes	10	Dead	48	No	Yes	Yes	No	24	No	No	Yes	Yes	No	No	No	No	No	No	No	No	No	Yes	Yes
CORE-0219	COVID	10	12	Yes	10	Dead	10	No	Yes	Yes	No	29	No	No	Yes	Yes	No	Yes	No	No	No	No	No	No	No	Yes	Yes
CORE-0220	COVID	5	4	No	10	Dead	8	No	Yes	Yes	No	47	No	Yes	No	No	No	Yes	No	No	No	No	Yes	No	Yes	Yes	
CORE-0221	COVID	28	28	Yes	10	Dead	17	No	Yes	Yes	No	28	No	Yes	No	No	No	No	No	No	No	No	No	No	No	Yes	Yes
CORE-0222	COVID	5	4	Yes	10	Dead	20	No	No	Yes	No	46	Yes	Yes	No	No	Yes	No	No	No	Yes	No	No	No	No	Yes	Yes
CORE-0223	COVID	14	14	Yes	10	Dead	11	Yes	Yes	Yes	No	27	Yes	Yes	Yes	No	No	No	No	No	No	No	No	No	No	Yes	Yes
CORE-0224	COVID	5	5	No	6	Live	5	No	No	Yes	No	26	Yes	Yes	No	No	Yes	Yes	No	No	Yes	No	No	Yes	Yes	Yes	Yes
CORE-0225	COVID	8	8	Yes	8	Live	28	No	Yes	Yes	No	31	Yes	Yes	No	No	No	No	No	No	No	No	No	No	No	Yes	
CORE-0226	COVID	2	9	No	5	Live	3	No	No	Yes	No	29	No	No	No	No	No	No	No	No	No	No	No	No	No	No	
CORE-0227	COVID	6	6	Yes	7	Live	8	No	Yes	Yes	No	25	Yes	Yes	No	No	No	No	No	No	No	No	No	No	No	Yes	Yes
CORE-0228	COVID	4	4	Yes	9	Live	55	No	Yes	Yes	No	33	Yes	Yes	Yes	No	Yes	No	No	Yes	Yes	No	No	No	No	Yes	Yes
CORE-0229	COVID	2	2	No	10	Dead	7	No	Yes	Yes	No	17	No	Yes	No	No	No	No	Yes	No	No	No	No	No	No	Yes	Yes
CORE-0230	COVID	2	2	No	6	Live	11	Yes	Yes	No	No	40	Yes	Yes	No	No	No	No	No	No	No	No	Yes	No	No	Yes	
CORE-0232	COVID	-8	0	No	5	Live	7	No	Yes	Yes	No	37	No	Yes	No	No	No	No	No	Yes	No	No	No	No	No	Yes	Yes
CORE-0233	COVID	3	3	No	5	Live	32	Yes	No	Yes	No	24	No	Yes	Yes	Yes	Yes	No	No	No	No	No	No	No	No	Yes	Yes
CORE-0234	COVID	7	7	No	4	Live	8	No	No	No	No	33	No	Yes	Yes	No	No	No	No	No	No	No	No	No	No	Yes	
CORE-0235	COVID	1	1	No	5	Live	14	No	No	Yes	No	25	Yes	Yes	No	No	No	No	No	No	Yes	No	No	No	No	Yes	Yes
CORE-0236	COVID	2	6	Yes	8	Live	13	No	Yes	Yes	No	27	Yes	Yes	No	No	No	No	No	No	No	No	No	No	No	Yes	Yes
CORE-0237	COVID	2	2	No	4	Live	29	No	Yes	No	No	48	Yes	Yes	No	Yes	Yes	Yes	Yes	Yes	Yes	No	No	No	No	Yes	
CORE-0238	COVID	3	7	No	4	Live	3	No	Yes	No	No	43	Yes	Yes	No	Yes	No	Yes	No	Yes	No	No	No	No	No	Yes	
CORE-0239	COVID	3	3	Yes	8	Live	60	No	Yes	Yes	No	39	Yes	Yes	No	No	No	Yes	No	No	No	No	No	No	No	Yes	
CORE-0240	COVID	7	7	Yes	10	Dead	7	No	Yes	No	No	25	No	Yes	No	Yes	Yes	No	No	Yes	Yes	No	No	No	No	Yes	
CORE-0242	COVID	0	0	Yes	9	Live	61	No	Yes	No	No	28	No	Yes	No	No	No	No	No	No	No	No	No	No	No	Yes	
CORE-0244	COVID	21	21	No	4	Live	3	No	No	No	No	23	No	Yes	No	No	No	No	No	No	Yes	No	No	No	No	Yes	Yes
CORE-0245	COVID	7	7	No	6	Live	8	Yes	Yes	No	No	27	No	Yes	No	No	No	No	No	No	No	No	No	No	No	Yes	Yes
CORE-0246	COVID	10	18	No	4	Live	2	No	No	Yes	No	30	No	Yes	No	No	No	Yes	No	No	No	No	No	No	No	Yes	
CORE-0247	COVID	6	5	No	6	Live	8	Yes	Yes	No	No	27	No	No	No	No	No	No	No	Yes	No	No	No	No	No	Yes	Yes
CORE-0248	COVID	12	11	Yes	9	Live	34	Yes	Yes	No	No	40	No	Yes	No	No	No	Yes	No	No	No	No	No	No	No	Yes	Yes
CORE-0251	COVID	19	14	Yes	10	Dead	25	No	Yes	No	No	22	No	Yes	No	No	No	Yes	Yes	Yes	No	No	Yes	No	Yes	Yes	

CORE-0252	COVID	5	4	Yes	10	Dead	122	No	Yes	Yes	No	29	No	No	Yes	Yes	No	No	No	No	No	No	No	No	No	Yes	Yes
CORE-0253	COVID	0	3	No	5	Live	4	No	No	No	No	21	No	Yes	No	No	No	No	Yes	No	Yes	No	No	No	No	Yes	
CORE-0254	COVID	7	14	No	4	Live	15	No	Yes	No	No	29	No	Yes	Yes	No	Yes	No	No	No	No	No	No	Yes	No	Yes	Yes
CORE-0255	COVID	40	40	Yes	9	Live	70	No	Yes	No	No	33	Yes	Yes	No	No	No	No	No	No	No	No	No	No	No	Yes	
CORE-0256	COVID	6	5	Yes	10	Dead	10	No	Yes	No	No	45	Yes	Yes	No	No	No	No	No	No	No	No	Yes	No	Yes	Yes	Yes
CORE-0257	COVID	8	7	No	5	Live	15	Yes	No	No	No	31	No	Yes	No	No	Yes	No	No	No	No	No	No	No	No	Yes	Yes
CORE-0258	COVID	0	7	No	10	Dead	18	No	No	No	No	27	Yes	Yes	No	No	No	No	No	No	No	No	No	No	No	Yes	
CORE-0259	COVID	7	7	No	6	Live	29	No	No	Yes	No	43	Yes	Yes	Yes	No	Yes	No	No	No	No	No	No	No	No	Yes	
CORE-0260	COVID	0	7	Yes	10	Dead	21	Yes	Yes	No	No	30	Yes	No	No	No	No	No	No	No	No	No	No	No	No	Yes	Yes
CORE-0261	COVID	0	2	Yes	9	Live	52	No	Yes	No	No	39	Yes	Yes	No	Yes	Yes	No	No	No	Yes	No	No	No	No	Yes	Yes
CORE-0262	COVID	0	0	No	10	Dead	10	No	Yes	No	No	17	Yes	Yes	No	Yes	Yes	No	No	No	No	Yes	No	No	No	Yes	Yes
CORE-0263	COVID	1	1	Yes	8	Live	51	No	Yes	No	No	42	Yes	Yes	No	Yes	Yes	No	Yes	Yes	No	Yes	No	No	No	Yes	Yes
CORE-0264	COVID	0	0	No	10	Dead	18	No	No	No	No	20	No	No	No	No	No	No	No	No	No	No	Yes	No	Yes	Yes	
CORE-0266	COVID	0	0	Yes	8	Live	24	No	Yes	No	No	38	No	Yes	No	No	Yes	No	No	Yes	Yes	No	No	No	No	Yes	
CORE-0269	COVID	3	0	Yes	7	Live	63	No	Yes	No	No	22	No	Yes	Yes	No	Yes	No	No	No	Yes	No	No	Yes	Yes	Yes	Yes
CORE-0270	COVID	3	3	No	4	Live	3	No	No	No	No	26	No	No	No	No	No	No	No	No	No	No	No	No	No	No	Yes
CORE-0271	COVID	7	14	No	4	Live	8	No	No	No	No	32	Yes	Yes	No	No	Yes	No	Yes	No	No	No	No	No	No	Yes	
CORE-0272	COVID	1	1	Yes	10	Dead	27	Yes	Yes	No	No	33	Yes	Yes	Yes	Yes	No	Yes	No	No	No	No	No	No	No	Yes	Yes
CORE-0275	COVID	7	7	Yes	10	Dead	16	Yes	Yes	No	Yes	30	No	Yes	No	No	No	No	No	Yes	No	No	Yes	No	Yes	Yes	
CORE-0276	COVID	0	4	No	4	Live	11	No	No	No	No	27	No	Yes	No	Yes	No	No	No	No	No	No	No	No	No	Yes	
CORE-0277	COVID	0	0	No	6	Live	10	No	No	No	No	19	No	No	No	No	No	No	Yes	No	No	No	No	No	No	Yes	
CORE-0279	COVID	0	0	Yes	10	Dead	3	No	Yes	No	No	22	Yes	Yes	Yes	No	Yes	No	No	No	No	No	No	No	No	Yes	
CORE-0280	COVID	0	0	No	4	Live	7	No	No	No	No	28	No	Yes	Yes	Yes	Yes	No	No	No	No	No	No	No	No	Yes	
CORE-0282	COVID	5	5	Yes	9	Live	18	No	Yes	No	Yes	32	Yes	No	No	No	Yes	No	No	No	No	No	No	No	No	Yes	
CORE-0284	COVID	5	5	No	4	Live	7	No	No	No	No	18	No	Yes	No	No	No	No	No	No	No	No	Yes	No	Yes	Yes	
CORE-0285	COVID	22	28	No	8	Live	23	Yes	Yes	No	No	23	No	No	No	No	No	No	No	No	No	No	No	No	No	No	
CORE-0286	COVID	2	3	No	4	Live	11	No	No	No	No	42	No	Yes	No	No	Yes	No	No	No	No	No	No	No	No	Yes	
CORE-0288	COVID	10	17	Yes	9	Live	85	Yes	Yes	No	Yes	28	No	No	No	No	Yes	No	No	No	No	No	No	No	No	Yes	
CORE-0289	COVID	3	1	No	6	Live	5	Yes	Yes	No	No	39	Yes	Yes	No	Yes	Yes	No	No	Yes	No	No	No	No	No	Yes	
CORE-0290	COVID	8	7	No	4	Live	9	No	Yes	No	No	17	No	Yes	No	No	No	No	No	No	No	No	No	No	No	Yes	
CORE-0291	COVID	1	4	No	4	Live	3	No	No	No	No	43	No	Yes	No	No	No	No	No	No	No	No	No	No	No	Yes	
CORE-0292	COVID	6	5	No	5	Live	3	No	Yes	No	No	35	Yes	Yes	No	No	No	No	No	Yes	No	No	No	No	No	Yes	
CORE-0293	COVID	7	7	No	4	Live	7	No	Yes	No	No	50	Yes	No	No	No	Yes	No	No	No	No	No	No	No	No	Yes	
CORE-0297	COVID	2	2	No	4	Live	2	No	No	No	No	39	No	No	No	No	No	No	No	No	No	No	Yes	No	Yes	Yes	
CORE-0298	COVID	0	2	No	8	Live	10	No	Yes	No	Yes	21	No	Yes	No	No	Yes	No	No	Yes	No	No	No	No	No	Yes	
**NON-COVID**																											
CORE-0179	ARDS (culture-neg pneumonia vs lung drug toxicity)			Yes		Live	37					31	Yes	Yes	Yes	No	No	No	Yes	No	No	No	No	No	No	Yes	
CORE-0180	MRSA septic shock & pneumonia			Yes		Dead	44					22	No	No	No	No	No	No	Yes	No	No	No	No	No	No	Yes	
CORE-0181	post-cardiac arrest, end stage renal disease			Yes		Dead	13					29	Yes	No	No	No	No	No	No	No	Yes	No	No	No	No	Yes	
CORE-0182	diarrhea, sepsis (culture neg), acute kidey injury			No		Live	13					23	No	Yes	No	No	No	No	No	No	No	No	No	No	No	Yes	
CORE-0184	acute pancreatitis & sepsis			Yes		Dead	35					22	Yes	Yes	Yes	No	No	No	No	No	No	No	No	Yes	Yes	Yes	
CORE-0185	acute pulmonary emboli, aspiration pneumonia			Yes		Live	32					23	No	No	No	Yes	No	No	No	Yes	No	No	Yes	No	Yes	Yes	
CORE-0187	respiratory failure due to H. flu, rhino/enterovirus			Yes		Live	27					28	No	Yes	No	Yes	Yes	No	No	No	Yes	No	No	No	No	Yes	
CORE-0188	acute pancreatitis, resp failure			Yes		Dead	42					31	No	No	Yes	No	No	No	No	No	No	No	No	No	No	Yes	
CORE-0189	septic shock (culture neg), end stage renal disease			No		Dead	32					20	Yes	Yes	No	No	Yes	No	No	No	Yes	No	No	No	No	Yes	
CORE-0190	pneumonia due to aspiration & rhino/enterovirus+			Yes		Dead	17					26	Yes	No	No	No	No	No	No	Yes	No	No	Yes	No	No	Yes	
CORE-0294	COPD exacerbation, hypercapnea			No		Live	2					21	No	Yes	No	No	No	No	Yes	No	No	No	No	No	No	Yes	
CORE-0295	septic shock due to biliary obstruction and cholangitis			No		Live	19					22	No	No	No	No	No	No	No	Yes	No	No	Yes	No	Yes	Yes	
CORE-0296	sepsis due to urinary source, hypercapnea			No		Live	10					19	Yes	Yes	No	No	Yes	No	No	No	No	No	Yes	No	No	Yes	
