# Supplementary material for: Signatures of COVID-19 Severity and Immune Response in the Respiratory Tract Microbiome
Source: mBio. 2021 Aug 17;12(4):e01777-21. doi: 10.1128/mBio.01777-21 (PMC8406335; doi:10.1128/mBio.01777-21)
Supplement: TABLE S2 [file mbio.01777-21-st002.pdf]

Table S2. Samples analyzed by 16S rRNA marker gene sequencing.

SampleID	sample_type	dna_kit	SubjectID	study_day	study_group	host_species	sample_ldms	dtt_added	dna_conc_ng_ul	library_conc_ng_ul	vol_for.epMotion
CORE0181.V1.nonCOVID.NP.PS	Nasopharyngeal swab	PS	CORE0181	V1	non COVID	Human	None	No	None	35.08	2.9
CORE0181.V1.nonCOVID.OP.PS	Oropharyngeal swab	PS	CORE0181	V1	non COVID	Human	None	No	None	21.93	4.6
CORE0181.V2.nonCOVID.ETA.PRO	Endotracheal aspirate	PRO	CORE0181	V2	non COVID	Human	None	No	None	64.91	1.5
CORE0181.V2.nonCOVID.NP.PS	Nasopharyngeal swab	PS	CORE0181	V2	non COVID	Human	None	No	None	20.01	5
CORE0181.V2.nonCOVID.OP.PS	Oropharyngeal swab	PS	CORE0181	V2	non COVID	Human	None	No	None	13.4	7.5
CORE0181.V3.nonCOVID.ETA.PRO	Endotracheal aspirate	PRO	CORE0181	V3	non COVID	Human	None	No	None	53.54	1.9
CORE0181.V3.nonCOVID.NP.PS	Nasopharyngeal swab	PS	CORE0181	V3	non COVID	Human	None	No	None	18.49	5.4
CORE0181.V3.nonCOVID.OP.PS	Oropharyngeal swab	PS	CORE0181	V3	non COVID	Human	None	No	None	17.42	5.7
CORE0181.V4.nonCOVID.NP.PS	Nasopharyngeal swab	PS	CORE0181	V4	non COVID	Human	None	No	None	16.54	6.1
CORE0181.V4.nonCOVID.OP.PS	Oropharyngeal swab	PS	CORE0181	V4	non COVID	Human	None	No	None	61.99	1.6
CORE0189.V1.nonCOVID.NP.PS	Nasopharyngeal swab	PS	CORE0189	V1	non COVID	Human	None	No	None	73.98	1.5
CORE0189.V1.nonCOVID.OP.PS	Oropharyngeal swab	PS	CORE0189	V1	non COVID	Human	None	No	None	69.18	1.5
CORE0189.V2.nonCOVID.NP.PS	Nasopharyngeal swab	PS	CORE0189	V2	non COVID	Human	None	No	None	28.03	3.6
CORE0189.V2.nonCOVID.OP.PS	Oropharyngeal swab	PS	CORE0189	V2	non COVID	Human	None	No	None	30.68	3.3
CORE0189.V3.nonCOVID.ETA.PRO	Endotracheal aspirate	PRO	CORE0189	V3	non COVID	Human	None	No	None	7.8	12.8
CORE0189.V3.nonCOVID.NP.PS	Nasopharyngeal swab	PS	CORE0189	V3	non COVID	Human	None	No	None	30.65	3.3
CORE0189.V3.nonCOVID.OP.PS	Oropharyngeal swab	PS	CORE0189	V3	non COVID	Human	None	No	None	9.36	10.7
CORE0189.V4.nonCOVID.ETA.PRO	Endotracheal aspirate	PRO	CORE0189	V4	non COVID	Human	None	No	None	10.67	9.4
CORE0189.V4.nonCOVID.NP.PS	Nasopharyngeal swab	PS	CORE0189	V4	non COVID	Human	None	No	None	36.07	2.8
CORE0189.V4.nonCOVID.OP.PS	Oropharyngeal swab	PS	CORE0189	V4	non COVID	Human	None	No	None	62.18	1.6
CORE0206.V1.COVID.ETA.PRO	Endotracheal aspirate	PRO	CORE0206	V1	COVID	Human	None	No	None	8.53	11.7
CORE0206.V1.COVID.NP.PRO	Nasopharyngeal swab	PRO	CORE0206	V1	COVID	Human	None	No	141.97	22.71	4.4
CORE0206.V1.COVID.OP.PRO	Oropharyngeal swab	PRO	CORE0206	V1	COVID	Human	None	No	153.54	21.13	4.7
CORE0206.V2.COVID.NP.PRO	Nasopharyngeal swab	PRO	CORE0206	V2	COVID	Human	None	No	98.82	28.3	3.5
CORE0206.V2.COVID.OP.PRO	Oropharyngeal swab	PRO	CORE0206	V2	COVID	Human	None	No	149.49	16.23	6.2
CORE0206.V3.COVID.ETA.PRO	Endotracheal aspirate	PRO	CORE0206	V3	COVID	Human	None	No	None	12.57	8
CORE0206.V3.COVID.NP.PRO	Nasopharyngeal swab	PRO	CORE0206	V3	COVID	Human	None	No	14.67	47	2.1
CORE0206.V3.COVID.OP.PRO	Oropharyngeal swab	PRO	CORE0206	V3	COVID	Human	None	No	6.75	59.29	1.7
CORE0207.V1.COVID.NP.PRO	Nasopharyngeal swab	PRO	CORE0207	V1	COVID	Human	None	No	10.1	37.71	2.7
CORE0207.V1.COVID.OP.PRO	Oropharyngeal swab	PRO	CORE0207	V1	COVID	Human	None	No	2.34	84.93	1.5
CORE0207.V2.COVID.NP.PRO	Nasopharyngeal swab	PRO	CORE0207	V2	COVID	Human	None	No	139.93	30.37	3.3
CORE0207.V2.COVID.OP.PRO	Oropharyngeal swab	PRO	CORE0207	V2	COVID	Human	None	No	147.16	22.99	4.4
CORE0207.V3.COVID.NP.PRO	Nasopharyngeal swab	PRO	CORE0207	V3	COVID	Human	None	No	4.09	1.69	38
CORE0207.V3.COVID.OP.PRO	Oropharyngeal swab	PRO	CORE0207	V3	COVID	Human	None	No	16.24	53.19	1.9
CORE0208.V1.COVID.NP.PRO	Nasopharyngeal swab	PRO	CORE0208	V1	COVID	Human	None	No	36.3	51.51	1.9
CORE0208.V1.COVID.OP.PRO	Oropharyngeal swab	PRO	CORE0208	V1	COVID	Human	None	No	4.63	15.51	6.5
CORE0208.V2.COVID.NP.PRO	Nasopharyngeal swab	PRO	CORE0208	V2	COVID	Human	None	No	5.45	38.85	2.6
CORE0208.V2.COVID.OP.PRO	Oropharyngeal swab	PRO	CORE0208	V2	COVID	Human	None	No	4.09	32.53	3.1
CORE0209.V1.COVID.NP.PRO	Nasopharyngeal swab	PRO	CORE0209	V1	COVID	Human	None	No	11.55	5.27	19
CORE0209.V1.COVID.OP.PRO	Oropharyngeal swab	PRO	CORE0209	V1	COVID	Human	None	No	18.97	88.78	1.5
CORE0209.V2.COVID.ETA.PRO	Endotracheal aspirate	PRO	CORE0209	V2	COVID	Human	None	No	None	6.14	16.3
CORE0209.V2.COVID.NP.PRO	Nasopharyngeal swab	PRO	CORE0209	V2	COVID	Human	None	No	114.11	43.95	2.3
CORE0209.V2.COVID.OP.PRO	Oropharyngeal swab	PRO	CORE0209	V2	COVID	Human	None	No	43.76	86.3	1.5
CORE0209.V3.COVID.NP.PRO	Nasopharyngeal swab	PRO	CORE0209	V3	COVID	Human	None	No	69.03	19.16	5.2
CORE0209.V3.COVID.OP.PRO	Oropharyngeal swab	PRO	CORE0209	V3	COVID	Human	None	No	2.13	71.62	1.5
CORE0216.V1.COVID.OP.PRO	Oropharyngeal swab	PRO	CORE0216	V1	COVID	Human	None	No	114.77	47.01	2.1
CORE0216.V2.COVID.OP.PRO	Oropharyngeal swab	PRO	CORE0216	V2	COVID	Human	None	No	91.09	73.22	1.5
CORE0216.V3.COVID.OP.PRO	Oropharyngeal swab	PRO	CORE0216	V3	COVID	Human	None	No	41.99	88.29	1.5
CORE0216.V4.COVID.OP.PRO	Oropharyngeal swab	PRO	CORE0216	V4	COVID	Human	None	No	43.76	2.3	38
CORE0220.V1.COVID.NP.PRO	Nasopharyngeal swab	PRO	CORE0220	V1	COVID	Human	None	No	16.61	57.65	1.7
CORE0220.V1.COVID.OP.PRO	Oropharyngeal swab	PRO	CORE0220	V1	COVID	Human	None	No	6.39	71.01	1.5
CORE0223.V1.COVID.OP.PRO	Oropharyngeal swab	PRO	CORE0223	V1	COVID	Human	None	No	None	58.72	1.7
CORE0223.V2.COVID.OP.PRO	Oropharyngeal swab	PRO	CORE0223	V2	COVID	Human	None	No	None	43.47	2.3
CORE0224.V1.COVID.OP.PRO	Oropharyngeal swab	PRO	CORE0224	V1	COVID	Human	None	No	None	80.08	1.5
CORE0229.V1.COVID.NP.PRO	Nasopharyngeal swab	PRO	CORE0229	V1	COVID	Human	None	No	None	58.11	1.7
CORE0229.V1.COVID.OP.PRO	Oropharyngeal swab	PRO	CORE0229	V1	COVID	Human	None	No	None	74.99	1.5
CORE0232.V1.COVID.NP.PRO	Nasopharyngeal swab	PRO	CORE0232	V1	COVID	Human	None	No	None	35.21	2.8
CORE0232.V1.COVID.OP.PRO	Oropharyngeal swab	PRO	CORE0232	V1	COVID	Human	None	No	None	81.21	1.5
CORE0232.V2.COVID.NP.PRO	Nasopharyngeal swab	PRO	CORE0232	V2	COVID	Human	None	No	None	61.08	1.6
CORE0232.V2.COVID.OP.PRO	Oropharyngeal swab	PRO	CORE0232	V2	COVID	Human	None	No	None	77.88	1.5
CORE0234.V1.COVID.NP.PRO	Nasopharyngeal swab	PRO	CORE0234	V1	COVID	Human	None	No	None	49.79	2
CORE0234.V1.COVID.OP.PRO	Oropharyngeal swab	PRO	CORE0234	V1	COVID	Human	None	No	None	75.64	1.5
CORE0234.V2.COVID.NP.PRO	Nasopharyngeal swab	PRO	CORE0234	V2	COVID	Human	None	No	None	60.16	1.7
CORE0234.V2.COVID.OP.PRO	Oropharyngeal swab	PRO	CORE0234	V2	COVID	Human	None	No	None	85.33	1.5
CORE0235.V1.COVID.NP.PRO	Nasopharyngeal swab	PRO	CORE0235	V1	COVID	Human	None	No	None	66.33	1.5
CORE0235.V1.COVID.OP.PRO	Oropharyngeal swab	PRO	CORE0235	V1	COVID	Human	None	No	None	94.83	1.5
CORE0235.V2.COVID.NP.PRO	Nasopharyngeal swab	PRO	CORE0235	V2	COVID	Human	None	No	None	82.98	1.5
CORE0235.V2.COVID.OP.PRO	Oropharyngeal swab	PRO	CORE0235	V2	COVID	Human	None	No	None	75.78	1.5
CORE0235.V3.COVID.NP.PRO	Nasopharyngeal swab	PRO	CORE0235	V3	COVID	Human	None	No	None	61.9	1.6
CORE0235.V3.COVID.OP.PRO	Oropharyngeal swab	PRO	CORE0235	V3	COVID	Human	None	No	None	79.77	1.5
CORE0246.V1.COVID.NP.PRO	Nasopharyngeal swab	PRO	CORE0246	V1	COVID	Human	None	No	None	10.29	9.7
CORE0246.V1.COVID.OP.PRO	Oropharyngeal swab	PRO	CORE0246	V1	COVID	Human	None	No	None	45.35	2.2
CORE0248.V1.COVID.NP.PRO	Nasopharyngeal swab	PRO	CORE0248	V1	COVID	Human	None	No	None	49.3	2
CORE0248.V1.COVID.OP.PRO	Oropharyngeal swab	PRO	CORE0248	V1	COVID	Human	None	No	None	46.79	2.1
CORE0248.V2.COVID.ETA.PRO	Endotracheal aspirate	PRO	CORE0248	V2	COVID	Human	None	No	139.87	12.54	8
CORE0248.V2.COVID.NP.PRO	Nasopharyngeal swab	PRO	CORE0248	V2	COVID	Human	None	No	None	49.76	2
CORE0248.V2.COVID.OP.PRO	Oropharyngeal swab	PRO	CORE0248	V2	COVID	Human	None	No	None	40.67	2.5
CORE0248.V3.COVID.ETA.PRO	Endotracheal aspirate	PRO	CORE0248	V3	COVID	Human	None	No	165.45	23.55	4.3
CORE0248.V3.COVID.NP.PRO	Nasopharyngeal swab	PRO	CORE0248	V3	COVID	Human	None	No	None	60.31	1.7
CORE0248.V3.COVID.OP.PRO	Oropharyngeal swab	PRO	CORE0248	V3	COVID	Human	None	No	None	53.56	1.9
CORE0248.V4.COVID.ETA.PRO	Endotracheal aspirate	PRO	CORE0248	V4	COVID	Human	None	No	102.93	16.88	5.9
CORE0248.V4.COVID.NP.PRO	Nasopharyngeal swab	PRO	CORE0248	V4	COVID	Human	None	No	None	73.71	1.5
CORE0248.V4.COVID.OP.PRO	Oropharyngeal swab	PRO	CORE0248	V4	COVID	Human	None	No	None	61.64	1.6
CORE0248.V5.COVID.ETA.PRO	Endotracheal aspirate	PRO	CORE0248	V5	COVID	Human	None	No	95.69	11.67	8.6
CORE0248.V5.COVID.NP.PRO	Nasopharyngeal swab	PRO	CORE0248	V5	COVID	Human	None	No	None	53.83	1.9
CORE0248.V5.COVID.OP.PRO	Oropharyngeal swab	PRO	CORE0248	V5	COVID	Human	None	No	None	51.71	1.9
CORE0248.V6.COVID.ETA.PRO	Endotracheal aspirate	PRO	CORE0248	V6	COVID	Human	None	No	113.4	13.46	7.4
CORE0248.V6.COVID.NP.PRO	Nasopharyngeal swab	PRO	CORE0248	V6	COVID	Human	None	No	None	57.96	1.7
CORE0248.V6.COVID.OP.PRO	Oropharyngeal swab	PRO	CORE0248	V6	COVID	Human	None	No	None	56.43	1.8
CORE0251.V1.COVID.NP.PRO	Nasopharyngeal swab	PRO	CORE0251	V1	COVID	Human	None	No	None	41.3	2.4
CORE0251.V1.COVID.OP.PRO	Oropharyngeal swab	PRO	CORE0251	V1	COVID	Human	None	No	None	55.97	1.8
CORE0251.V2.COVID.ETA.PRO	Endotracheal aspirate	PRO	CORE0251	V2	COVID	Human	None	No	6.25	16.4	6.1
CORE0251.V2.COVID.NP.PRO	Nasopharyngeal swab	PRO	CORE0251	V2	COVID	Human	None	No	None	20.57	4.9
CORE0251.V2.COVID.OP.PRO	Oropharyngeal swab	PRO	CORE0251	V2	COVID	Human	None	No	None	52.53	1.9
CORE0251.V3.COVID.NP.PRO	Nasopharyngeal swab	PRO	CORE0251	V3	COVID	Human	None	No	None	38.53	2.6
CORE0251.V3.COVID.OP.PRO	Oropharyngeal swab	PRO	CORE0251	V3	COVID	Human	None	No	None	45.3	2.2
CORE0251.V4.COVID.NP.PRO	Nasopharyngeal swab	PRO	CORE0251	V4	COVID	Human	None	No	None	38.11	2.6
CORE0251.V4.COVID.OP.PRO	Oropharyngeal swab	PRO	CORE0251	V4	COVID	Human	None	No	None	60.32	1.7
CORE0254.V1.COVID.NP.PRO	Nasopharyngeal swab	PRO	CORE0254	V1	COVID	Human	None	No	None	54.81	1.8
CORE0254.V1.COVID.OP.PRO	Oropharyngeal swab	PRO	CORE0254	V1	COVID	Human	None	No	None	62.05	1.6
CORE0254.V2.COVID.NP.PRO	Nasopharyngeal swab	PRO	CORE0254	V2	COVID	Human	None	No	None	43.02	2.3
CORE0254.V2.COVID.OP.PRO	Oropharyngeal swab	PRO	CORE0254	V2	COVID	Human	None	No	None	68.44	1.5
CORE0260.V1.COVID.NP.PRO	Nasopharyngeal swab	PRO	CORE0260	V1	COVID	Human	None	No	None	23.72	4.2
CORE0260.V1.COVID.OP.PRO	Oropharyngeal swab	PRO	CORE0260	V1	COVID	Human	None	No	None	61.27	1.6
CORE0260.V2.COVID.NP.PRO	Nasopharyngeal swab	PRO	CORE0260	V2	COVID	Human	None	No	None	59.03	1.7

CORE0260.V2.COVID.OP.PRO	Oropharyngeal swab	PRO	CORE0260	V2	COVID	Human	None	No	None	69.46	1.5
CORE0260.V3.COVID.NP.PRO	Nasopharyngeal swab	PRO	CORE0260	V3	COVID	Human	None	No	None	26.81	3.7
CORE0260.V3.COVID.OP.PRO	Oropharyngeal swab	PRO	CORE0260	V3	COVID	Human	None	No	None	67.66	1.5
CORE0260.V4.COVID.NP.PRO	Nasopharyngeal swab	PRO	CORE0260	V4	COVID	Human	None	No	None	46.55	2.2
CORE0260.V4.COVID.OP.PRO	Oropharyngeal swab	PRO	CORE0260	V4	COVID	Human	None	No	None	64.59	1.6
CORE0264.V2.COVID.ETA.PRO	Endotracheal aspirate	PRO	CORE0264	V2	COVID	Human	None	No	3.93	41.96	2.4
CORE0264.V2.COVID.NP.PRO	Nasopharyngeal swab	PRO	CORE0264	V2	COVID	Human	None	No	None	0.24	38
CORE0264.V2.COVID.OP.PRO	Oropharyngeal swab	PRO	CORE0264	V2	COVID	Human	None	No	None	69.17	1.5
CORE0269.V1.COVID.NP.PRO	Nasopharyngeal swab	PRO	CORE0269	V1	COVID	Human	None	No	None	23.62	4.2
CORE0269.V2.COVID.ETA.PRO	Endotracheal aspirate	PRO	CORE0269	V2	COVID	Human	None	No	84.53	9.44	10.6
CORE0269.V2.COVID.NP.PRO	Nasopharyngeal swab	PRO	CORE0269	V2	COVID	Human	None	No	None	36.22	2.8
CORE0269.V2.COVID.OP.PRO	Oropharyngeal swab	PRO	CORE0269	V2	COVID	Human	None	No	None	59.77	1.7
CORE0269.V3.COVID.ETA.PRO	Endotracheal aspirate	PRO	CORE0269	V3	COVID	Human	None	No	135.6	11.59	8.6
CORE0269.V3.COVID.NP.PRO	Nasopharyngeal swab	PRO	CORE0269	V3	COVID	Human	None	No	None	9.25	10.8
CORE0269.V3.COVID.OP.PRO	Oropharyngeal swab	PRO	CORE0269	V3	COVID	Human	None	No	None	42.77	2.3
CORE0269.V4.COVID.ETA.PRO	Endotracheal aspirate	PRO	CORE0269	V4	COVID	Human	None	No	45.92	24.54	4.1
CORE0269.V4.COVID.NP.PRO	Nasopharyngeal swab	PRO	CORE0269	V4	COVID	Human	None	No	None	1.6	36
CORE0269.V4.COVID.OP.PRO	Oropharyngeal swab	PRO	CORE0269	V4	COVID	Human	None	No	None	60.23	1.7
CORE0269.V5.COVID.NP.PRO	Nasopharyngeal swab	PRO	CORE0269	V5	COVID	Human	None	No	None	11.01	9.1
CORE0269.V5.COVID.OP.PRO	Oropharyngeal swab	PRO	CORE0269	V5	COVID	Human	None	No	None	63.87	1.5
CORE0270.V1.COVID.NP.PRO	Nasopharyngeal swab	PRO	CORE0270	V1	COVID	Human	None	No	None	0.61	36
CORE0270.V1.COVID.OP.PRO	Oropharyngeal swab	PRO	CORE0270	V1	COVID	Human	None	No	None	0.21	36
CORE0277.V1.COVID.NP.PRO	Nasopharyngeal swab	PRO	CORE0277	V1	COVID	Human	None	No	None	0.42	36
CORE0277.V1.COVID.OP.PRO	Oropharyngeal swab	PRO	CORE0277	V1	COVID	Human	None	No	None	0.35	36
CORE0277.V2.COVID.NP.PRO	Nasopharyngeal swab	PRO	CORE0277	V2	COVID	Human	None	No	None	3.06	32.7
CORE0277.V2.COVID.OP.PRO	Oropharyngeal swab	PRO	CORE0277	V2	COVID	Human	None	No	None	6.57	15.2
CORE0279.V1.COVID.NP.PRO	Nasopharyngeal swab	PRO	CORE0279	V1	COVID	Human	None	No	None	35.82	2.8
CORE0279.V1.COVID.OP.PRO	Oropharyngeal swab	PRO	CORE0279	V1	COVID	Human	None	No	None	52.69	1.9
CORE0284.V1.COVID.NP.PRO	Nasopharyngeal swab	PRO	CORE0284	V1	COVID	Human	None	No	None	59.08	1.7
CORE0284.V1.COVID.OP.PRO	Oropharyngeal swab	PRO	CORE0284	V1	COVID	Human	None	No	None	70.51	1.5
CORE0286.V1.COVID.NP.PRO	Nasopharyngeal swab	PRO	CORE0286	V1	COVID	Human	None	No	None	1.2	38
CORE0286.V1.COVID.OP.PRO	Oropharyngeal swab	PRO	CORE0286	V1	COVID	Human	None	No	None	1.73	38
CORE0286.V2.COVID.NP.PRO	Nasopharyngeal swab	PRO	CORE0286	V2	COVID	Human	None	No	None	4.7	21.3
CORE0286.V2.COVID.OP.PRO	Oropharyngeal swab	PRO	CORE0286	V2	COVID	Human	None	No	None	10.48	9.5
CORE0292.V1.COVID.NP.PRO	Nasopharyngeal swab	PRO	CORE0292	V1	COVID	Human	None	No	None	42.5	2.4
CORE0292.V1.COVID.OP.PRO	Oropharyngeal swab	PRO	CORE0292	V1	COVID	Human	None	No	None	51.84	1.9
CORE0295.V1.nonCOVID.NP.PRO	Nasopharyngeal swab	PRO	CORE0295	V1	non COVID	Human	None	No	None	2.18	38
CORE0295.V1.nonCOVID.NP.PS	Nasopharyngeal swab	PS	CORE0295	V1	non COVID	Human	None	No	None	20.83	4.8
CORE0295.V1.nonCOVID.OP.PRO	Oropharyngeal swab	PRO	CORE0295	V1	non COVID	Human	None	No	None	8.48	11.8
CORE0295.V1.nonCOVID.OP.PS	Oropharyngeal swab	PS	CORE0295	V1	non COVID	Human	None	No	None	15.68	6.4
CORE0266.V1.COVID.NP.PRO	Nasopharyngeal swab	PRO	CORE0266	V1	COVID	Human	None	No	None	3.47	28.8
CORE0266.V1.COVID.OP.PRO	Oropharyngeal swab	PRO	CORE0266	V1	COVID	Human	None	No	None	62.66	1.6
CORE0266.V2.COVID.NP.PRO	Nasopharyngeal swab	PRO	CORE0266	V2	COVID	Human	None	No	None	9.64	10.4
CORE0266.V2.COVID.OP.PRO	Oropharyngeal swab	PRO	CORE0266	V2	COVID	Human	None	No	None	62.6	1.6
CORE0185.V1.nonCOVID.NP.PS	Nasopharyngeal swab	PS	CORE0185	V1	non COVID	Human	None	No	None	27.14	3.7
CORE0185.V1.nonCOVID.OP.PS	Oropharyngeal swab	PS	CORE0185	V1	non COVID	Human	None	No	None	58.44	1.7
CORE0185.V2.nonCOVID.ETA.PRO	Endotracheal aspirate	PRO	CORE0185	V2	non COVID	Human	None	No	None	0.26	38
CORE0185.V2.nonCOVID.NP.PS	Nasopharyngeal swab	PS	CORE0185	V2	non COVID	Human	None	No	None	8.76	11.4
CORE0185.V2.nonCOVID.OP.PS	Oropharyngeal swab	PS	CORE0185	V2	non COVID	Human	None	No	None	2.73	36.6
CORE0185.V3.nonCOVID.NP.PS	Nasopharyngeal swab	PS	CORE0185	V3	non COVID	Human	None	No	None	7.22	13.9
CORE0185.V3.nonCOVID.OP.PS	Oropharyngeal swab	PS	CORE0185	V3	non COVID	Human	None	No	None	13.91	7.2
CORE0185.V4.nonCOVID.NP.PS	Nasopharyngeal swab	PS	CORE0185	V4	non COVID	Human	None	No	None	8.21	12.2
CORE0185.V4.nonCOVID.OP.PS	Oropharyngeal swab	PS	CORE0185	V4	non COVID	Human	None	No	None	9.14	10.9
CORE0193.V5.COVID.NP.PRO	Nasopharyngeal swab	PRO	CORE0193	V5	COVID	Human	None	No	0.16	13.86	7.2
CORE0193.V5.COVID.OP.PRO	Oropharyngeal swab	PRO	CORE0193	V5	COVID	Human	None	No	2.64	49.73	2
CORE0213.V1.COVID.NP.PRO	Nasopharyngeal swab	PRO	CORE0213	V1	COVID	Human	None	No	48.18	73.4	1.5
CORE0213.V1.COVID.OP.PRO	Oropharyngeal swab	PRO	CORE0213	V1	COVID	Human	None	No	11.26	55.21	1.8
CORE0213.V2.COVID.NP.PRO	Nasopharyngeal swab	PRO	CORE0213	V2	COVID	Human	None	No	63.33	73.38	1.5
CORE0213.V2.COVID.OP.PRO	Oropharyngeal swab	PRO	CORE0213	V2	COVID	Human	None	No	13.61	53.91	1.9
CORE0213.V3.COVID.ETA.PRO	Endotracheal aspirate	PRO	CORE0213	V3	COVID	Human	None	No	30.55	14.67	6.8
CORE0213.V3.COVID.NP.PRO	Nasopharyngeal swab	PRO	CORE0213	V3	COVID	Human	None	No	4.83	101.07	1.5
CORE0213.V3.COVID.OP.PRO	Oropharyngeal swab	PRO	CORE0213	V3	COVID	Human	None	No	16.61	51.46	1.9
CORE0233.V1.COVID.NP.PRO	Nasopharyngeal swab	PRO	CORE0233	V1	COVID	Human	None	No	None	56.5	1.8
CORE0233.V1.COVID.OP.PRO	Oropharyngeal swab	PRO	CORE0233	V1	COVID	Human	None	No	None	74.48	1.5
CORE0233.V2.COVID.NP.PRO	Nasopharyngeal swab	PRO	CORE0233	V2	COVID	Human	None	No	None	39.09	2.6
CORE0233.V2.COVID.OP.PRO	Oropharyngeal swab	PRO	CORE0233	V2	COVID	Human	None	No	None	58.05	1.7
CORE0233.V3.COVID.NP.PRO	Nasopharyngeal swab	PRO	CORE0233	V3	COVID	Human	None	No	None	54.73	1.8
CORE0233.V3.COVID.OP.PRO	Oropharyngeal swab	PRO	CORE0233	V3	COVID	Human	None	No	None	65.28	1.5
CORE0233.V4.COVID.NP.PRO	Nasopharyngeal swab	PRO	CORE0233	V4	COVID	Human	None	No	None	61.66	1.6
CORE0233.V4.COVID.OP.PRO	Oropharyngeal swab	PRO	CORE0233	V4	COVID	Human	None	No	None	43.85	2.3
CORE0238.V1.COVID.NP.PRO	Nasopharyngeal swab	PRO	CORE0238	V1	COVID	Human	None	No	None	15.28	6.5
CORE0238.V1.COVID.OP.PRO	Oropharyngeal swab	PRO	CORE0238	V1	COVID	Human	None	No	None	46.39	2.2
CORE0252.V1.COVID.NP.PRO	Nasopharyngeal swab	PRO	CORE0252	V1	COVID	Human	None	No	None	33.43	3
CORE0252.V1.COVID.OP.PRO	Oropharyngeal swab	PRO	CORE0252	V1	COVID	Human	None	No	None	37.67	2.7
CORE0252.V2.COVID.ETA.PRO	Endotracheal aspirate	PRO	CORE0252	V2	COVID	Human	None	No	123.18	23.14	4.3
CORE0252.V2.COVID.NP.PRO	Nasopharyngeal swab	PRO	CORE0252	V2	COVID	Human	None	No	None	24.38	4.1
CORE0252.V2.COVID.OP.PRO	Oropharyngeal swab	PRO	CORE0252	V2	COVID	Human	None	No	None	56.34	1.8
CORE0252.V3.COVID.ETA.PRO	Endotracheal aspirate	PRO	CORE0252	V3	COVID	Human	None	No	130.09	12.05	8.3
CORE0252.V3.COVID.NP.PRO	Nasopharyngeal swab	PRO	CORE0252	V3	COVID	Human	None	No	None	42.67	2.3
CORE0252.V3.COVID.OP.PRO	Oropharyngeal swab	PRO	CORE0252	V3	COVID	Human	None	No	None	58.05	1.7
CORE0252.V4.COVID.NP.PRO	Nasopharyngeal swab	PRO	CORE0252	V4	COVID	Human	None	No	None	7.82	12.8
CORE0252.V4.COVID.OP.PRO	Oropharyngeal swab	PRO	CORE0252	V4	COVID	Human	None	No	None	69.16	1.5
CORE0252.V5.COVID.ETA.PRO	Endotracheal aspirate	PRO	CORE0252	V5	COVID	Human	None	No	13.71	36.64	2.7
CORE0252.V5.COVID.NP.PRO	Nasopharyngeal swab	PRO	CORE0252	V5	COVID	Human	None	No	None	33.54	3
CORE0252.V5.COVID.OP.PRO	Oropharyngeal swab	PRO	CORE0252	V5	COVID	Human	None	No	None	72.17	1.5
CORE0252.V6.COVID.NP.PRO	Nasopharyngeal swab	PRO	CORE0252	V6	COVID	Human	None	No	None	41.79	2.4
CORE0252.V6.COVID.OP.PRO	Oropharyngeal swab	PRO	CORE0252	V6	COVID	Human	None	No	None	53.87	1.9
CORE0262.V1.COVID.NP.PRO	Nasopharyngeal swab	PRO	CORE0262	V1	COVID	Human	None	No	None	46.91	2.1
CORE0262.V2.COVID.NP.PRO	Nasopharyngeal swab	PRO	CORE0262	V2	COVID	Human	None	No	None	60.17	1.7
CORE0262.V2.COVID.OP.PRO	Oropharyngeal swab	PRO	CORE0262	V2	COVID	Human	None	No	None	53.26	1.9
CORE0263.V1.COVID.ETA.PRO	Endotracheal aspirate	PRO	CORE0263	V1	COVID	Human	None	No	67.62	2.72	36.8
CORE0263.V1.COVID.NP.PRO	Nasopharyngeal swab	PRO	CORE0263	V1	COVID	Human	None	No	None	42.13	2.4
CORE0263.V1.COVID.OP.PRO	Oropharyngeal swab	PRO	CORE0263	V1	COVID	Human	None	No	None	61.02	1.6
CORE0263.V2.COVID.NP.PRO	Nasopharyngeal swab	PRO	CORE0263	V2	COVID	Human	None	No	None	63.05	1.6
CORE0263.V2.COVID.OP.PRO	Oropharyngeal swab	PRO	CORE0263	V2	COVID	Human	None	No	None	63.78	1.6
CORE0263.V3.COVID.NP.PRO	Nasopharyngeal swab	PRO	CORE0263	V3	COVID	Human	None	No	None	64.6	1.6
CORE0263.V3.COVID.OP.PRO	Oropharyngeal swab	PRO	CORE0263	V3	COVID	Human	None	No	None	63.11	1.6
CORE0263.V4.COVID.NP.PRO	Nasopharyngeal swab	PRO	CORE0263	V4	COVID	Human	None	No	None	69.92	1.5
CORE0263.V4.COVID.OP.PRO	Oropharyngeal swab	PRO	CORE0263	V4	COVID	Human	None	No	None	63.91	1.6
CORE0263.V5.COVID.ETA.PRO	Endotracheal aspirate	PRO	CORE0263	V5	COVID	Human	None	No	89.08	13.15	7.6
CORE0263.V5.COVID.NP.PRO	Nasopharyngeal swab	PRO	CORE0263	V5	COVID	Human	None	No	None	26.66	3.8
CORE0263.V5.COVID.OP.PRO	Oropharyngeal swab	PRO	CORE0263	V5	COVID	Human	None	No	None	64.32	1.6
CORE0263.V6.COVID.ETA.PRO	Endotracheal aspirate	PRO	CORE0263	V6	COVID	Human	None	No	113.36	30.33	3.3
CORE0263.V6.COVID.NP.PRO	Nasopharyngeal swab	PRO	CORE0263	V6	COVID	Human	None	No	None	47.58	2.1
CORE0263.V6.COVID.OP.PRO	Oropharyngeal swab	PRO	CORE0263	V6	COVID	Human	None	No	None	61.44	1.6
CORE0272.V1.COVID.NP.PRO	Nasopharyngeal swab	PRO	CORE0272	V1	COVID	Human	None	No	None	41.45	2.4
CORE0272.V1.COVID.OP.PRO	Oropharyngeal swab	PRO	CORE0272	V1	COVID	Human	None	No	None	65.64	1.5

CORE0272.V2.COVID.ETA.PRO	Endotracheal aspirate	PRO	CORE0272	V2	COVID	Human	None	No	90.59	10.32	9.7
CORE0272.V2.COVID.NP.PRO	Nasopharyngeal swab	PRO	CORE0272	V2	COVID	Human	None	No	None	36.5	2.7
CORE0272.V2.COVID.OP.PRO	Oropharyngeal swab	PRO	CORE0272	V2	COVID	Human	None	No	None	55.76	1.8
CORE0272.V3.COVID.NP.PRO	Nasopharyngeal swab	PRO	CORE0272	V3	COVID	Human	None	No	None	0.25	36
CORE0272.V3.COVID.OP.PRO	Oropharyngeal swab	PRO	CORE0272	V3	COVID	Human	None	No	None	0.38	36
CORE0272.V4.COVID.NP.PRO	Nasopharyngeal swab	PRO	CORE0272	V4	COVID	Human	None	No	None	15.07	6.6
CORE0272.V4.COVID.OP.PRO	Oropharyngeal swab	PRO	CORE0272	V4	COVID	Human	None	No	None	65.11	1.5
CORE0272.V5.COVID.ETA.PRO	Endotracheal aspirate	PRO	CORE0272	V5	COVID	Human	None	No	72	72.39	1.5
CORE0272.V5.COVID.NP.PRO	Nasopharyngeal swab	PRO	CORE0272	V5	COVID	Human	None	No	None	12.99	7.7
CORE0272.V5.COVID.OP.PRO	Oropharyngeal swab	PRO	CORE0272	V5	COVID	Human	None	No	None	64.15	1.6
CORE0272.V6.COVID.NP.PRO	Nasopharyngeal swab	PRO	CORE0272	V6	COVID	Human	None	No	None	32.79	3.1
CORE0272.V6.COVID.OP.PRO	Oropharyngeal swab	PRO	CORE0272	V6	COVID	Human	None	No	None	65.31	1.5
CORE0289.V1.COVID.NP.PRO	Nasopharyngeal swab	PRO	CORE0289	V1	COVID	Human	None	No	None	82.36	1.5
CORE0289.V1.COVID.OP.PRO	Oropharyngeal swab	PRO	CORE0289	V1	COVID	Human	None	No	None	63.32	1.6
CORE0179.V1.nonCOVID.NP.PS	Nasopharyngeal swab	PS	CORE0179	V1	non COVID	Human	None	No	None	0.34	38
CORE0179.V1.nonCOVID.OP.PS	Oropharyngeal swab	PS	CORE0179	V1	non COVID	Human	None	No	None	12.73	7.9
CORE0179.V2.nonCOVID.NP.PS	Nasopharyngeal swab	PS	CORE0179	V2	non COVID	Human	None	No	None	0.73	38
CORE0179.V2.nonCOVID.OP.PS	Oropharyngeal swab	PS	CORE0179	V2	non COVID	Human	None	No	None	5.06	19.8
CORE0182.V1.nonCOVID.NP.PS	Nasopharyngeal swab	PS	CORE0182	V1	non COVID	Human	None	No	None	3.29	30.4
CORE0182.V1.nonCOVID.OP.PS	Oropharyngeal swab	PS	CORE0182	V1	non COVID	Human	None	No	None	10.74	9.3
CORE0184.V1.nonCOVID.NP.PS	Nasopharyngeal swab	PS	CORE0184	V1	non COVID	Human	None	No	None	6.25	16
CORE0184.V1.nonCOVID.OP.PS	Oropharyngeal swab	PS	CORE0184	V1	non COVID	Human	None	No	None	0.35	38
CORE0190.V1.nonCOVID.NP.PS	Nasopharyngeal swab	PS	CORE0190	V1	non COVID	Human	None	No	None	16.41	6.1
CORE0190.V1.nonCOVID.OP.PS	Oropharyngeal swab	PS	CORE0190	V1	non COVID	Human	None	No	None	0.21	38
CORE0190.V2.nonCOVID.NP.PS	Nasopharyngeal swab	PS	CORE0190	V2	non COVID	Human	None	No	None	0.71	38
CORE0190.V2.nonCOVID.OP.PS	Oropharyngeal swab	PS	CORE0190	V2	non COVID	Human	None	No	None	0.87	38
CORE0196.V2.COVID.ETA.PRO	Endotracheal aspirate	PRO	CORE0196	V2	COVID	Human	None	No	None	48.06	2.1
CORE0196.V3.COVID.ETA.PRO	Endotracheal aspirate	PRO	CORE0196	V3	COVID	Human	None	No	None	32.45	3.1
CORE0196.V3.COVID.NP.PRO	Nasopharyngeal swab	PRO	CORE0196	V3	COVID	Human	None	No	73.91	41.25	2.4
CORE0196.V3.COVID.OP.PRO	Oropharyngeal swab	PRO	CORE0196	V3	COVID	Human	None	No	81.98	33.65	3
CORE0196.V4.COVID.NP.PRO	Nasopharyngeal swab	PRO	CORE0196	V4	COVID	Human	None	No	77.27	15.97	6.3
CORE0196.V4.COVID.OP.PRO	Oropharyngeal swab	PRO	CORE0196	V4	COVID	Human	None	No	74.96	64.88	1.5
CORE0196.V5.COVID.ETA.PRO	Endotracheal aspirate	PRO	CORE0196	V5	COVID	Human	None	No	None	13.45	7.4
CORE0196.V5.COVID.NP.PRO	Nasopharyngeal swab	PRO	CORE0196	V5	COVID	Human	None	No	114.94	22.52	4.4
CORE0196.V5.COVID.OP.PRO	Oropharyngeal swab	PRO	CORE0196	V5	COVID	Human	None	No	122.21	33.72	3
CORE0196.V6.COVID.NP.PRO	Nasopharyngeal swab	PRO	CORE0196	V6	COVID	Human	None	No	64.8	40.18	2.5
CORE0196.V6.COVID.OP.PRO	Oropharyngeal swab	PRO	CORE0196	V6	COVID	Human	None	No	119.63	24.03	4.2
CORE0196.V7.COVID.NP.PRO	Nasopharyngeal swab	PRO	CORE0196	V7	COVID	Human	None	No	71.58	40.57	2.5
CORE0196.V7.COVID.OP.PRO	Oropharyngeal swab	PRO	CORE0196	V7	COVID	Human	None	No	3.61	42.57	2.4
CORE0197.V2.COVID.ETA.PRO	Endotracheal aspirate	PRO	CORE0197	V2	COVID	Human	None	No	None	15.7	6.4
CORE0197.V3.COVID.NP.PRO	Nasopharyngeal swab	PRO	CORE0197	V3	COVID	Human	None	No	58.99	63.01	1.6
CORE0197.V3.COVID.OP.PRO	Oropharyngeal swab	PRO	CORE0197	V3	COVID	Human	None	No	54.72	83.34	1.5
CORE0197.V4.COVID.NP.PRO	Nasopharyngeal swab	PRO	CORE0197	V4	COVID	Human	None	No	111.91	71.11	1.5
CORE0197.V4.COVID.OP.PRO	Oropharyngeal swab	PRO	CORE0197	V4	COVID	Human	None	No	60.22	51.34	2
CORE0197.V5.COVID.NP.PRO	Nasopharyngeal swab	PRO	CORE0197	V5	COVID	Human	None	No	0.57	6	16.7
CORE0197.V5.COVID.OP.PRO	Oropharyngeal swab	PRO	CORE0197	V5	COVID	Human	None	No	53.23	41.48	2.4
CORE0197.V6.COVID.ETA.PRO	Endotracheal aspirate	PRO	CORE0197	V6	COVID	Human	None	No	None	22.84	4.4
CORE0197.V6.COVID.NP.PRO	Nasopharyngeal swab	PRO	CORE0197	V6	COVID	Human	None	No	92.26	13.76	7.3
CORE0197.V6.COVID.OP.PRO	Oropharyngeal swab	PRO	CORE0197	V6	COVID	Human	None	No	25.2	45.1	2.2
CORE0200.V1.COVID.NP.PRO	Nasopharyngeal swab	PRO	CORE0200	V1	COVID	Human	None	No	84.52	11.84	8.5
CORE0200.V1.COVID.OP.PRO	Oropharyngeal swab	PRO	CORE0200	V1	COVID	Human	None	No	1.78	53.5	1.9
CORE0201.V1.COVID.NP.PRO	Nasopharyngeal swab	PRO	CORE0201	V1	COVID	Human	None	No	0.66	16.57	6
CORE0201.V1.COVID.OP.PRO	Oropharyngeal swab	PRO	CORE0201	V1	COVID	Human	None	No	0.82	32.7	3.1
CORE0202.V1.COVID.NP.PRO	Nasopharyngeal swab	PRO	CORE0202	V1	COVID	Human	None	No	15.32	49.21	2
CORE0202.V1.COVID.OP.PRO	Oropharyngeal swab	PRO	CORE0202	V1	COVID	Human	None	No	2.7	46.85	2.1
CORE0203.V1.COVID.ETA.PRO	Endotracheal aspirate	PRO	CORE0203	V1	COVID	Human	None	No	None	19.31	5.2
CORE0203.V1.COVID.NP.PRO	Nasopharyngeal swab	PRO	CORE0203	V1	COVID	Human	None	No	66.16	40.05	2.5
CORE0203.V1.COVID.OP.PRO	Oropharyngeal swab	PRO	CORE0203	V1	COVID	Human	None	No	28.52	53.54	1.9
CORE0203.V2.COVID.ETA.PRO	Endotracheal aspirate	PRO	CORE0203	V2	COVID	Human	None	No	None	30.15	3.3
CORE0203.V2.COVID.NP.PRO	Nasopharyngeal swab	PRO	CORE0203	V2	COVID	Human	None	No	84.66	34.39	2.9
CORE0203.V2.COVID.OP.PRO	Oropharyngeal swab	PRO	CORE0203	V2	COVID	Human	None	No	22.06	43.39	2.3
CORE0203.V3.COVID.ETA.PRO	Endotracheal aspirate	PRO	CORE0203	V3	COVID	Human	None	No	None	27.86	3.6
CORE0203.V3.COVID.NP.PRO	Nasopharyngeal swab	PRO	CORE0203	V3	COVID	Human	None	No	7.01	38.23	2.6
CORE0203.V3.COVID.OP.PRO	Oropharyngeal swab	PRO	CORE0203	V3	COVID	Human	None	No	58.79	42.76	2.3
CORE0203.V4.COVID.ETA.PRO	Endotracheal aspirate	PRO	CORE0203	V4	COVID	Human	None	No	None	13.72	7.3
CORE0203.V4.COVID.NP.PRO	Nasopharyngeal swab	PRO	CORE0203	V4	COVID	Human	None	No	75.84	20.38	4.9
CORE0203.V4.COVID.OP.PRO	Oropharyngeal swab	PRO	CORE0203	V4	COVID	Human	None	No	18.59	44	2.3
CORE0203.V5.COVID.NP.PRO	Nasopharyngeal swab	PRO	CORE0203	V5	COVID	Human	None	No	82.54	30.79	3.3
CORE0203.V5.COVID.OP.PRO	Oropharyngeal swab	PRO	CORE0203	V5	COVID	Human	None	No	16.85	48.71	2.1
CORE0210.V1.COVID.ETA.PRO	Endotracheal aspirate	PRO	CORE0210	V1	COVID	Human	None	No	None	13.76	7.3
CORE0210.V1.COVID.NP.PRO	Nasopharyngeal swab	PRO	CORE0210	V1	COVID	Human	None	No	70.74	61.64	1.6
CORE0210.V1.COVID.OP.PRO	Oropharyngeal swab	PRO	CORE0210	V1	COVID	Human	None	No	24.98	53.71	1.9
CORE0210.V2.COVID.NP.PRO	Nasopharyngeal swab	PRO	CORE0210	V2	COVID	Human	None	No	72.69	76.87	1.5
CORE0210.V2.COVID.OP.PRO	Oropharyngeal swab	PRO	CORE0210	V2	COVID	Human	None	No	75.03	75.88	1.5
CORE0210.V3.COVID.ETA.PRO	Endotracheal aspirate	PRO	CORE0210	V3	COVID	Human	None	No	None	11.48	8.7
CORE0210.V3.COVID.NP.PRO	Nasopharyngeal swab	PRO	CORE0210	V3	COVID	Human	None	No	95.12	49.31	2
CORE0210.V3.COVID.OP.PRO	Oropharyngeal swab	PRO	CORE0210	V3	COVID	Human	None	No	95.03	66.7	1.5
CORE0210.V4.COVID.NP.PRO	Nasopharyngeal swab	PRO	CORE0210	V4	COVID	Human	None	No	31.21	84.53	1.5
CORE0210.V4.COVID.OP.PRO	Oropharyngeal swab	PRO	CORE0210	V4	COVID	Human	None	No	75.42	64.47	1.6
CORE0210.V5.COVID.ETA.PRO	Endotracheal aspirate	PRO	CORE0210	V5	COVID	Human	None	No	None	26.59	3.8
CORE0210.V5.COVID.NP.PRO	Nasopharyngeal swab	PRO	CORE0210	V5	COVID	Human	None	No	70.63	63.26	1.6
CORE0210.V5.COVID.OP.PRO	Oropharyngeal swab	PRO	CORE0210	V5	COVID	Human	None	No	34.27	71.55	1.5
CORE0211.V1.COVID.NP.PRO	Nasopharyngeal swab	PRO	CORE0211	V1	COVID	Human	None	No	35.69	3.52	28.4
CORE0211.V1.COVID.OP.PRO	Oropharyngeal swab	PRO	CORE0211	V1	COVID	Human	None	No	103.65	66.91	1.5
CORE0211.V2.COVID.ETA.PRO	Endotracheal aspirate	PRO	CORE0211	V2	COVID	Human	None	No	54.85	11.78	8.5
CORE0211.V2.COVID.OP.PRO	Oropharyngeal swab	PRO	CORE0211	V2	COVID	Human	None	No	112.61	72.47	1.5
CORE0212.V1.COVID.NP.PRO	Nasopharyngeal swab	PRO	CORE0212	V1	COVID	Human	None	No	19.61	37.89	2.6
CORE0212.V1.COVID.OP.PRO	Oropharyngeal swab	PRO	CORE0212	V1	COVID	Human	None	No	45.22	58.38	1.7
CORE0212.V2.COVID.NP.PRO	Nasopharyngeal swab	PRO	CORE0212	V2	COVID	Human	None	No	41.1	3.35	29.9
CORE0212.V2.COVID.OP.PRO	Oropharyngeal swab	PRO	CORE0212	V2	COVID	Human	None	No	8.31	1.63	38
CORE0212.V3.COVID.NP.PRO	Nasopharyngeal swab	PRO	CORE0212	V3	COVID	Human	None	No	1.71	0.63	38
CORE0212.V3.COVID.OP.PRO	Oropharyngeal swab	PRO	CORE0212	V3	COVID	Human	None	No	6.06	4.41	22.7
CORE0212.V4.COVID.ETA.PRO	Endotracheal aspirate	PRO	CORE0212	V4	COVID	Human	None	No	16.24	0.6	38
CORE0212.V4.COVID.NP.PRO	Nasopharyngeal swab	PRO	CORE0212	V4	COVID	Human	None	No	0.01	0.24	38
CORE0212.V4.COVID.OP.PRO	Oropharyngeal swab	PRO	CORE0212	V4	COVID	Human	None	No	0.04	0.29	38
CORE0212.V5.COVID.ETA.PRO	Endotracheal aspirate	PRO	CORE0212	V5	COVID	Human	None	No	39.87	1.29	38
CORE0212.V5.COVID.NP.PRO	Nasopharyngeal swab	PRO	CORE0212	V5	COVID	Human	None	No	1.08	0.7	38
CORE0212.V5.COVID.OP.PRO	Oropharyngeal swab	PRO	CORE0212	V5	COVID	Human	None	No	-0.01	0.21	38
CORE0212.V6.COVID.ETA.PRO	Endotracheal aspirate	PRO	CORE0212	V6	COVID	Human	None	No	2.21	0.55	38
CORE0212.V6.COVID.NP.PRO	Nasopharyngeal swab	PRO	CORE0212	V6	COVID	Human	None	No	2.75	2.73	36.6
CORE0212.V6.COVID.OP.PRO	Oropharyngeal swab	PRO	CORE0212	V6	COVID	Human	None	No	6.46	3.54	28.3
CORE0215.V1.COVID.NP.PRO	Nasopharyngeal swab	PRO	CORE0215	V1	COVID	Human	None	No	60.79	34.12	2.9
CORE0215.V1.COVID.OP.PRO	Oropharyngeal swab	PRO	CORE0215	V1	COVID	Human	None	No	1.27	67.47	1.5
CORE0221.V1.COVID.NP.PRO	Nasopharyngeal swab	PRO	CORE0221	V1	COVID	Human	None	No	78.77	34.49	2.9
CORE0221.V1.COVID.OP.PRO	Oropharyngeal swab	PRO	CORE0221	V1	COVID	Human	None	No	73.45	66.72	1.5
CORE0221.V2.COVID.NP.PRO	Nasopharyngeal swab	PRO	CORE0221	V2	COVID	Human	None	No	72.01	37.79	2.7
CORE0221.V2.COVID.OP.PRO	Oropharyngeal swab	PRO	CORE0221	V2	COVID	Human	None	No	9.54	77.32	1.5

CORE0222.V1.COVID.NP.PRO	Nasopharyngeal swab	PRO	CORE0222	V1	COVID	Human	None	No	None	26.61	3.8
CORE0222.V1.COVID.OP.PRO	Oropharyngeal swab	PRO	CORE0222	V1	COVID	Human	None	No	None	71.71	1.5
CORE0222.V2.COVID.NP.PRO	Nasopharyngeal swab	PRO	CORE0222	V2	COVID	Human	None	No	None	19.61	5.1
CORE0222.V2.COVID.OP.PRO	Oropharyngeal swab	PRO	CORE0222	V2	COVID	Human	None	No	None	64.62	1.6
CORE0222.V3.COVID.NP.PRO	Nasopharyngeal swab	PRO	CORE0222	V3	COVID	Human	None	No	None	40.19	2.5
CORE0222.V3.COVID.OP.PRO	Oropharyngeal swab	PRO	CORE0222	V3	COVID	Human	None	No	None	65.6	1.5
CORE0222.V4.COVID.NP.PRO	Nasopharyngeal swab	PRO	CORE0222	V4	COVID	Human	None	No	None	60.98	1.6
CORE0222.V4.COVID.OP.PRO	Oropharyngeal swab	PRO	CORE0222	V4	COVID	Human	None	No	None	62.87	1.6
CORE0225.V1.COVID.ETA.PRO	Endotracheal aspirate	PRO	CORE0225	V1	COVID	Human	None	No	1.26	46.27	2.2
CORE0225.V1.COVID.NP.PRO	Nasopharyngeal swab	PRO	CORE0225	V1	COVID	Human	None	No	None	43.64	2.3
CORE0225.V1.COVID.OP.PRO	Oropharyngeal swab	PRO	CORE0225	V1	COVID	Human	None	No	None	68.6	1.5
CORE0225.V2.COVID.NP.PRO	Nasopharyngeal swab	PRO	CORE0225	V2	COVID	Human	None	No	None	53.27	1.9
CORE0225.V2.COVID.OP.PRO	Oropharyngeal swab	PRO	CORE0225	V2	COVID	Human	None	No	None	61.53	1.6
CORE0225.V3.COVID.NP.PRO	Nasopharyngeal swab	PRO	CORE0225	V3	COVID	Human	None	No	None	62.25	1.6
CORE0225.V3.COVID.OP.PRO	Oropharyngeal swab	PRO	CORE0225	V3	COVID	Human	None	No	None	77.9	1.5
CORE0225.V4.COVID.ETA.PRO	Endotracheal aspirate	PRO	CORE0225	V4	COVID	Human	None	No	73.86	37.41	2.7
CORE0225.V4.COVID.NP.PRO	Nasopharyngeal swab	PRO	CORE0225	V4	COVID	Human	None	No	None	52.29	1.9
CORE0225.V4.COVID.OP.PRO	Oropharyngeal swab	PRO	CORE0225	V4	COVID	Human	None	No	None	78.59	1.5
CORE0225.V5.COVID.NP.PRO	Nasopharyngeal swab	PRO	CORE0225	V5	COVID	Human	None	No	None	80.81	1.5
CORE0225.V5.COVID.OP.PRO	Oropharyngeal swab	PRO	CORE0225	V5	COVID	Human	None	No	None	79.41	1.5
CORE0225.V6.COVID.NP.PRO	Nasopharyngeal swab	PRO	CORE0225	V6	COVID	Human	None	No	None	53.06	1.9
CORE0225.V6.COVID.OP.PRO	Oropharyngeal swab	PRO	CORE0225	V6	COVID	Human	None	No	None	73.17	1.5
CORE0226.V1.COVID.NP.PRO	Nasopharyngeal swab	PRO	CORE0226	V1	COVID	Human	None	No	None	49.65	2
CORE0226.V1.COVID.OP.PRO	Oropharyngeal swab	PRO	CORE0226	V1	COVID	Human	None	No	None	75.21	1.5
CORE0227.V1.COVID.NP.PRO	Nasopharyngeal swab	PRO	CORE0227	V1	COVID	Human	None	No	None	71.3	1.5
CORE0227.V1.COVID.OP.PRO	Oropharyngeal swab	PRO	CORE0227	V1	COVID	Human	None	No	None	73.68	1.5
CORE0227.V2.COVID.NP.PRO	Nasopharyngeal swab	PRO	CORE0227	V2	COVID	Human	None	No	None	45.48	2.2
CORE0227.V2.COVID.OP.PRO	Oropharyngeal swab	PRO	CORE0227	V2	COVID	Human	None	No	None	73.72	1.5
CORE0228.V1.COVID.NP.PRO	Nasopharyngeal swab	PRO	CORE0228	V1	COVID	Human	None	No	None	43.67	2.3
CORE0228.V1.COVID.OP.PRO	Oropharyngeal swab	PRO	CORE0228	V1	COVID	Human	None	No	None	77.57	1.5
CORE0228.V2.COVID.NP.PRO	Nasopharyngeal swab	PRO	CORE0228	V2	COVID	Human	None	No	None	53.48	1.9
CORE0228.V2.COVID.OP.PRO	Oropharyngeal swab	PRO	CORE0228	V2	COVID	Human	None	No	None	104.41	1.5
CORE0228.V3.COVID.NP.PRO	Nasopharyngeal swab	PRO	CORE0228	V3	COVID	Human	None	No	None	14.56	6.9
CORE0228.V3.COVID.OP.PRO	Oropharyngeal swab	PRO	CORE0228	V3	COVID	Human	None	No	None	50.8	2
CORE0228.V4.COVID.NP.PRO	Nasopharyngeal swab	PRO	CORE0228	V4	COVID	Human	None	No	None	70.32	1.5
CORE0228.V4.COVID.OP.PRO	Oropharyngeal swab	PRO	CORE0228	V4	COVID	Human	None	No	None	79.66	1.5
CORE0228.V5.COVID.NP.PRO	Nasopharyngeal swab	PRO	CORE0228	V5	COVID	Human	None	No	None	49.14	2
CORE0228.V5.COVID.OP.PRO	Oropharyngeal swab	PRO	CORE0228	V5	COVID	Human	None	No	None	83.23	1.5
CORE0230.V1.COVID.NP.PRO	Nasopharyngeal swab	PRO	CORE0230	V1	COVID	Human	None	No	None	29.45	3.4
CORE0230.V1.COVID.OP.PRO	Oropharyngeal swab	PRO	CORE0230	V1	COVID	Human	None	No	None	81.88	1.5
CORE0230.V2.COVID.NP.PRO	Nasopharyngeal swab	PRO	CORE0230	V2	COVID	Human	None	No	None	2.57	38
CORE0230.V2.COVID.OP.PRO	Oropharyngeal swab	PRO	CORE0230	V2	COVID	Human	None	No	None	64.5	1.6
CORE0236.V1.COVID.ETA.PRO	Endotracheal aspirate	PRO	CORE0236	V1	COVID	Human	None	No	49.84	29.18	3.4
CORE0236.V1.COVID.NP.PRO	Nasopharyngeal swab	PRO	CORE0236	V1	COVID	Human	None	No	None	50.17	2
CORE0236.V1.COVID.OP.PRO	Oropharyngeal swab	PRO	CORE0236	V1	COVID	Human	None	No	None	79.99	1.5
CORE0239.V1.COVID.NP.PRO	Nasopharyngeal swab	PRO	CORE0239	V1	COVID	Human	None	No	None	9.02	11.1
CORE0239.V1.COVID.OP.PRO	Oropharyngeal swab	PRO	CORE0239	V1	COVID	Human	None	No	None	46.01	2.2
CORE0242.V1.COVID.NP.PRO	Nasopharyngeal swab	PRO	CORE0242	V1	COVID	Human	None	No	None	30.32	3.3
CORE0242.V1.COVID.OP.PRO	Oropharyngeal swab	PRO	CORE0242	V1	COVID	Human	None	No	None	45.85	2.2
CORE0242.V2.COVID.NP.PRO	Nasopharyngeal swab	PRO	CORE0242	V2	COVID	Human	None	No	None	12.85	7.8
CORE0242.V2.COVID.OP.PRO	Oropharyngeal swab	PRO	CORE0242	V2	COVID	Human	None	No	None	13.59	7.4
CORE0242.V3.COVID.NP.PRO	Nasopharyngeal swab	PRO	CORE0242	V3	COVID	Human	None	No	None	4.53	22.1
CORE0242.V3.COVID.OP.PRO	Oropharyngeal swab	PRO	CORE0242	V3	COVID	Human	None	No	None	60.44	1.7
CORE0242.V4.COVID.ETA.PRO	Endotracheal aspirate	PRO	CORE0242	V4	COVID	Human	None	No	37.89	3.28	30.5
CORE0242.V4.COVID.NP.PRO	Nasopharyngeal swab	PRO	CORE0242	V4	COVID	Human	None	No	None	19.38	5.2
CORE0242.V4.COVID.OP.PRO	Oropharyngeal swab	PRO	CORE0242	V4	COVID	Human	None	No	None	50.04	2
CORE0242.V5.COVID.ETA.PRO	Endotracheal aspirate	PRO	CORE0242	V5	COVID	Human	None	No	129.02	16.29	6.1
CORE0242.V5.COVID.NP.PRO	Nasopharyngeal swab	PRO	CORE0242	V5	COVID	Human	None	No	None	60.33	1.7
CORE0242.V5.COVID.OP.PRO	Oropharyngeal swab	PRO	CORE0242	V5	COVID	Human	None	No	None	54.8	1.8
CORE0242.V6.COVID.NP.PRO	Nasopharyngeal swab	PRO	CORE0242	V6	COVID	Human	None	No	None	41.1	2.4
CORE0242.V6.COVID.OP.PRO	Oropharyngeal swab	PRO	CORE0242	V6	COVID	Human	None	No	None	48.44	2.1
CORE0244.V1.COVID.NP.PRO	Nasopharyngeal swab	PRO	CORE0244	V1	COVID	Human	None	No	None	62.6	1.6
CORE0244.V1.COVID.OP.PRO	Oropharyngeal swab	PRO	CORE0244	V1	COVID	Human	None	No	None	64.72	1.6
CORE0245.V1.COVID.NP.PRO	Nasopharyngeal swab	PRO	CORE0245	V1	COVID	Human	None	No	None	0.7	38
CORE0245.V1.COVID.OP.PRO	Oropharyngeal swab	PRO	CORE0245	V1	COVID	Human	None	No	None	72.33	1.5
CORE0245.V2.COVID.NP.PRO	Nasopharyngeal swab	PRO	CORE0245	V2	COVID	Human	None	No	None	25.71	3.9
CORE0245.V2.COVID.OP.PRO	Oropharyngeal swab	PRO	CORE0245	V2	COVID	Human	None	No	None	61.12	1.6
CORE0247.V1.COVID.NP.PRO	Nasopharyngeal swab	PRO	CORE0247	V1	COVID	Human	None	No	None	55.63	1.8
CORE0247.V1.COVID.OP.PRO	Oropharyngeal swab	PRO	CORE0247	V1	COVID	Human	None	No	None	0.13	38
CORE0253.V1.COVID.NP.PRO	Nasopharyngeal swab	PRO	CORE0253	V1	COVID	Human	None	No	None	40.74	2.5
CORE0253.V1.COVID.OP.PRO	Oropharyngeal swab	PRO	CORE0253	V1	COVID	Human	None	No	None	57.85	1.7
CORE0255.V1.COVID.NP.PRO	Nasopharyngeal swab	PRO	CORE0255	V1	COVID	Human	None	No	None	33.05	3
CORE0255.V1.COVID.OP.PRO	Oropharyngeal swab	PRO	CORE0255	V1	COVID	Human	None	No	None	52.64	1.9
CORE0255.V2.COVID.NP.PRO	Nasopharyngeal swab	PRO	CORE0255	V2	COVID	Human	None	No	None	52.89	1.9
CORE0255.V2.COVID.OP.PRO	Oropharyngeal swab	PRO	CORE0255	V2	COVID	Human	None	No	None	28.83	3.5
CORE0255.V3.COVID.ETA.PRO	Endotracheal aspirate	PRO	CORE0255	V3	COVID	Human	None	No	108.44	11.74	8.5
CORE0255.V3.COVID.NP.PRO	Nasopharyngeal swab	PRO	CORE0255	V3	COVID	Human	None	No	None	35.79	2.8
CORE0255.V3.COVID.OP.PRO	Oropharyngeal swab	PRO	CORE0255	V3	COVID	Human	None	No	None	70.35	1.5
CORE0255.V4.COVID.NP.PRO	Nasopharyngeal swab	PRO	CORE0255	V4	COVID	Human	None	No	None	66.85	1.5
CORE0255.V4.COVID.OP.PRO	Oropharyngeal swab	PRO	CORE0255	V4	COVID	Human	None	No	None	44.19	2.3
CORE0255.V5.COVID.ETA.PRO	Endotracheal aspirate	PRO	CORE0255	V5	COVID	Human	None	No	52.9	28.79	3.5
CORE0255.V5.COVID.NP.PRO	Nasopharyngeal swab	PRO	CORE0255	V5	COVID	Human	None	No	None	52.93	1.9
CORE0255.V5.COVID.OP.PRO	Oropharyngeal swab	PRO	CORE0255	V5	COVID	Human	None	No	None	48.84	2.1
CORE0255.V6.COVID.ETA.PRO	Endotracheal aspirate	PRO	CORE0255	V6	COVID	Human	None	No	122.61	11.3	8.9
CORE0255.V6.COVID.NP.PRO	Nasopharyngeal swab	PRO	CORE0255	V6	COVID	Human	None	No	None	48.6	2.1
CORE0255.V6.COVID.OP.PRO	Oropharyngeal swab	PRO	CORE0255	V6	COVID	Human	None	No	None	45.78	2.2
CORE0255.V7.COVID.NP.PRO	Nasopharyngeal swab	PRO	CORE0255	V7	COVID	Human	None	No	None	75.29	1.5
CORE0255.V7.COVID.OP.PRO	Oropharyngeal swab	PRO	CORE0255	V7	COVID	Human	None	No	None	70.34	1.5
CORE0256.V1.COVID.ETA.PRO	Endotracheal aspirate	PRO	CORE0256	V1	COVID	Human	None	No	18.89	21.33	4.7
CORE0256.V1.COVID.NP.PRO	Nasopharyngeal swab	PRO	CORE0256	V1	COVID	Human	None	No	None	17.42	5.7
CORE0256.V1.COVID.OP.PRO	Oropharyngeal swab	PRO	CORE0256	V1	COVID	Human	None	No	None	18.49	5.4
CORE0256.V2.COVID.ETA.PRO	Endotracheal aspirate	PRO	CORE0256	V2	COVID	Human	None	No	45.09	18.98	5.3
CORE0256.V2.COVID.NP.PRO	Nasopharyngeal swab	PRO	CORE0256	V2	COVID	Human	None	No	None	0.08	38
CORE0256.V2.COVID.OP.PRO	Oropharyngeal swab	PRO	CORE0256	V2	COVID	Human	None	No	None	46.12	2.2
CORE0257.V1.COVID.NP.PRO	Nasopharyngeal swab	PRO	CORE0257	V1	COVID	Human	None	No	None	39.08	2.6
CORE0257.V1.COVID.OP.PRO	Oropharyngeal swab	PRO	CORE0257	V1	COVID	Human	None	No	None	53.02	1.9
CORE0257.V2.COVID.NP.PRO	Nasopharyngeal swab	PRO	CORE0257	V2	COVID	Human	None	No	None	24.33	4.1
CORE0257.V2.COVID.OP.PRO	Oropharyngeal swab	PRO	CORE0257	V2	COVID	Human	None	No	None	59.49	1.7
CORE0257.V3.COVID.NP.PRO	Nasopharyngeal swab	PRO	CORE0257	V3	COVID	Human	None	No	None	45.59	2.2
CORE0257.V3.COVID.OP.PRO	Oropharyngeal swab	PRO	CORE0257	V3	COVID	Human	None	No	None	62.37	1.6
CORE0258.V1.COVID.NP.PRO	Nasopharyngeal swab	PRO	CORE0258	V1	COVID	Human	None	No	None	15.34	6.5
CORE0258.V1.COVID.OP.PRO	Oropharyngeal swab	PRO	CORE0258	V1	COVID	Human	None	No	None	38.04	2.6
CORE0259.V2.COVID.NP.PRO	Nasopharyngeal swab	PRO	CORE0259	V2	COVID	Human	None	No	None	4.52	22.1
CORE0259.V2.COVID.OP.PRO	Oropharyngeal swab	PRO	CORE0259	V2	COVID	Human	None	No	None	46.68	2.1
CORE0271.V1.COVID.NP.PRO	Nasopharyngeal swab	PRO	CORE0271	V1	COVID	Human	None	No	None	1.03	36
CORE0271.V1.COVID.OP.PRO	Oropharyngeal swab	PRO	CORE0271	V1	COVID	Human	None	No	None	2.01	36
CORE0285.V1.COVID.NP.PRO	Nasopharyngeal swab	PRO	CORE0285	V1	COVID	Human	None	No	None	51.05	2

CORE0285.V1.COVID.OP.PRO	Oropharyngeal swab	PRO	CORE0285	V1	COVID	Human	None	No	None	99.57	1.5
CORE0285.V2.COVID.NP.PRO	Nasopharyngeal swab	PRO	CORE0285	V2	COVID	Human	None	No	None	40.9	2.4
CORE0285.V2.COVID.OP.PRO	Oropharyngeal swab	PRO	CORE0285	V2	COVID	Human	None	No	None	65.02	1.5
CORE0285.V3.COVID.NP.PRO	Nasopharyngeal swab	PRO	CORE0285	V3	COVID	Human	None	No	None	0.75	38
CORE0285.V3.COVID.OP.PRO	Oropharyngeal swab	PRO	CORE0285	V3	COVID	Human	None	No	None	3.38	29.6
CORE0285.V4.COVID.NP.PRO	Nasopharyngeal swab	PRO	CORE0285	V4	COVID	Human	None	No	None	23.87	4.2
CORE0285.V4.COVID.OP.PRO	Oropharyngeal swab	PRO	CORE0285	V4	COVID	Human	None	No	None	9.71	10.3
CORE0288.V1.COVID.NP.PRO	Nasopharyngeal swab	PRO	CORE0288	V1	COVID	Human	None	No	None	63.34	1.6
CORE0288.V1.COVID.OP.PRO	Oropharyngeal swab	PRO	CORE0288	V1	COVID	Human	None	No	None	90.47	1.5
CORE0288.V2.COVID.NP.PRO	Nasopharyngeal swab	PRO	CORE0288	V2	COVID	Human	None	No	None	25.1	4
CORE0288.V2.COVID.OP.PRO	Oropharyngeal swab	PRO	CORE0288	V2	COVID	Human	None	No	None	37.14	2.7
CORE0288.V3.COVID.ETA.PRO	Endotracheal aspirate	PRO	CORE0288	V3	COVID	Human	None	No	122.49	24.54	4.1
CORE0288.V3.COVID.NP.PRO	Nasopharyngeal swab	PRO	CORE0288	V3	COVID	Human	None	No	None	30.25	3.3
CORE0288.V3.COVID.OP.PRO	Oropharyngeal swab	PRO	CORE0288	V3	COVID	Human	None	No	None	55.62	1.8
CORE0288.V4.COVID.ETA.PRO	Endotracheal aspirate	PRO	CORE0288	V4	COVID	Human	None	No	83.18	26.36	3.8
CORE0288.V4.COVID.NP.PRO	Nasopharyngeal swab	PRO	CORE0288	V4	COVID	Human	None	No	None	64.92	1.5
CORE0288.V4.COVID.OP.PRO	Oropharyngeal swab	PRO	CORE0288	V4	COVID	Human	None	No	None	78.22	1.5
CORE0288.V5.COVID.ETA.PRO	Endotracheal aspirate	PRO	CORE0288	V5	COVID	Human	None	No	151.43	13.57	7.4
CORE0288.V5.COVID.NP.PRO	Nasopharyngeal swab	PRO	CORE0288	V5	COVID	Human	None	No	None	75.23	1.5
CORE0288.V5.COVID.OP.PRO	Oropharyngeal swab	PRO	CORE0288	V5	COVID	Human	None	No	None	81.75	1.5
CORE0288.V6.COVID.NP.PRO	Nasopharyngeal swab	PRO	CORE0288	V6	COVID	Human	None	No	None	2.55	38
CORE0288.V6.COVID.OP.PRO	Oropharyngeal swab	PRO	CORE0288	V6	COVID	Human	None	No	None	0.78	38
CORE0288.V7.COVID.NP.PRO	Nasopharyngeal swab	PRO	CORE0288	V7	COVID	Human	None	No	None	0.79	38
CORE0288.V7.COVID.OP.PRO	Oropharyngeal swab	PRO	CORE0288	V7	COVID	Human	None	No	None	0.66	38
CORE0288.V8.COVID.NP.PRO	Nasopharyngeal swab	PRO	CORE0288	V8	COVID	Human	None	No	None	51.93	1.9
CORE0288.V8.COVID.OP.PRO	Oropharyngeal swab	PRO	CORE0288	V8	COVID	Human	None	No	None	62.24	1.6
CORE0288.V9.COVID.NP.PRO	Nasopharyngeal swab	PRO	CORE0288	V9	COVID	Human	None	No	None	23.55	4.3
CORE0288.V9.COVID.OP.PRO	Oropharyngeal swab	PRO	CORE0288	V9	COVID	Human	None	No	None	0.63	38
CORE0290.V1.COVID.NP.PRO	Nasopharyngeal swab	PRO	CORE0290	V1	COVID	Human	None	No	None	76.78	1.5
CORE0290.V1.COVID.OP.PRO	Oropharyngeal swab	PRO	CORE0290	V1	COVID	Human	None	No	None	61.89	1.6
CORE0290.V2.COVID.NP.PRO	Nasopharyngeal swab	PRO	CORE0290	V2	COVID	Human	None	No	None	40.37	2.5
CORE0290.V2.COVID.OP.PRO	Oropharyngeal swab	PRO	CORE0290	V2	COVID	Human	None	No	None	75.64	1.5
CORE0291.V1.COVID.NP.PRO	Nasopharyngeal swab	PRO	CORE0291	V1	COVID	Human	None	No	None	77.11	1.5
CORE0291.V1.COVID.OP.PRO	Oropharyngeal swab	PRO	CORE0291	V1	COVID	Human	None	No	None	83.14	1.5
CORE0293.V1.COVID.NP.PRO	Nasopharyngeal swab	PRO	CORE0293	V1	COVID	Human	None	No	None	35.95	2.8
CORE0293.V1.COVID.OP.PRO	Oropharyngeal swab	PRO	CORE0293	V1	COVID	Human	None	No	None	65.47	1.5
CORE0297.V1.COVID.NP.PRO	Nasopharyngeal swab	PRO	CORE0297	V1	COVID	Human	None	No	None	0.46	38
CORE0297.V1.COVID.OP.PRO	Oropharyngeal swab	PRO	CORE0297	V1	COVID	Human	None	No	None	0.16	38
CORE0298.V1.COVID.NP.PRO	Nasopharyngeal swab	PRO	CORE0298	V1	COVID	Human	None	No	None	0.25	38
CORE0298.V1.COVID.OP.PRO	Oropharyngeal swab	PRO	CORE0298	V1	COVID	Human	None	No	None	0.21	38
CORE0298.V2.COVID.NP.PRO	Nasopharyngeal swab	PRO	CORE0298	V2	COVID	Human	None	No	None	6.09	16.4
CORE0298.V2.COVID.OP.PRO	Oropharyngeal swab	PRO	CORE0298	V2	COVID	Human	None	No	None	53.02	1.9
CORE0205.V1.COVID.NP.PRO	Nasopharyngeal swab	PRO	CORE0205	V1	COVID	Human	None	No	14.94	60.94	1.6
CORE0205.V1.COVID.OP.PRO	Oropharyngeal swab	PRO	CORE0205	V1	COVID	Human	None	No	0.79	78.04	1.5
CORE0205.V2.COVID.NP.PRO	Nasopharyngeal swab	PRO	CORE0205	V2	COVID	Human	None	No	34.5	57.06	1.8
CORE0205.V2.COVID.OP.PRO	Oropharyngeal swab	PRO	CORE0205	V2	COVID	Human	None	No	49.7	66.95	1.5
CORE0205.V3.COVID.NP.PRO	Nasopharyngeal swab	PRO	CORE0205	V3	COVID	Human	None	No	93.96	27.38	3.7
CORE0205.V3.COVID.OP.PRO	Oropharyngeal swab	PRO	CORE0205	V3	COVID	Human	None	No	182.27	48.57	2.1
CORE0218.V1.COVID.ETA.PRO	Endotracheal aspirate	PRO	CORE0218	V1	COVID	Human	None	No	31.18	19.21	5.2
CORE0218.V1.COVID.NP.PRO	Nasopharyngeal swab	PRO	CORE0218	V1	COVID	Human	None	No	70.67	60.66	1.7
CORE0218.V1.COVID.OP.PRO	Oropharyngeal swab	PRO	CORE0218	V1	COVID	Human	None	No	22.4	76.98	1.5
CORE0218.V2.COVID.ETA.PRO	Endotracheal aspirate	PRO	CORE0218	V2	COVID	Human	None	No	99.79	13.28	7.5
CORE0218.V2.COVID.NP.PRO	Nasopharyngeal swab	PRO	CORE0218	V2	COVID	Human	None	No	42.32	25.2	4
CORE0218.V2.COVID.OP.PRO	Oropharyngeal swab	PRO	CORE0218	V2	COVID	Human	None	No	34.98	83.11	1.5
CORE0218.V3.COVID.NP.PRO	Nasopharyngeal swab	PRO	CORE0218	V3	COVID	Human	None	No	55.39	50.25	2
CORE0218.V3.COVID.OP.PRO	Oropharyngeal swab	PRO	CORE0218	V3	COVID	Human	None	No	24.16	81.94	1.5
CORE0218.V4.COVID.NP.PRO	Nasopharyngeal swab	PRO	CORE0218	V4	COVID	Human	None	No	116.59	53.11	1.9
CORE0218.V4.COVID.OP.PRO	Oropharyngeal swab	PRO	CORE0218	V4	COVID	Human	None	No	3.26	53.91	1.9
CORE0218.V5.COVID.NP.PRO	Nasopharyngeal swab	PRO	CORE0218	V5	COVID	Human	None	No	25.41	50.94	2
CORE0218.V5.COVID.OP.PRO	Oropharyngeal swab	PRO	CORE0218	V5	COVID	Human	None	No	18.17	73.71	1.5
CORE0219.V1.COVID.NP.PRO	Nasopharyngeal swab	PRO	CORE0219	V1	COVID	Human	None	No	66.38	59.42	1.7
CORE0219.V1.COVID.OP.PRO	Oropharyngeal swab	PRO	CORE0219	V1	COVID	Human	None	No	124.8	49.52	2
CORE0219.V2.COVID.ETA.PRO	Endotracheal aspirate	PRO	CORE0219	V2	COVID	Human	None	No	94.87	25.38	3.9
CORE0219.V2.COVID.NP.PRO	Nasopharyngeal swab	PRO	CORE0219	V2	COVID	Human	None	No	28.41	60.35	1.7
CORE0219.V2.COVID.OP.PRO	Oropharyngeal swab	PRO	CORE0219	V2	COVID	Human	None	No	110.34	71.51	1.5
CORE0219.V3.COVID.NP.PRO	Nasopharyngeal swab	PRO	CORE0219	V3	COVID	Human	None	No	37.42	54.16	1.9
CORE0219.V3.COVID.OP.PRO	Oropharyngeal swab	PRO	CORE0219	V3	COVID	Human	None	No	75.76	53.52	1.9
CORE0237.V1.COVID.NP.PRO	Nasopharyngeal swab	PRO	CORE0237	V1	COVID	Human	None	No	None	45.76	2.2
CORE0237.V1.COVID.OP.PRO	Oropharyngeal swab	PRO	CORE0237	V1	COVID	Human	None	No	None	85.5	1.5
CORE0237.V2.COVID.NP.PRO	Nasopharyngeal swab	PRO	CORE0237	V2	COVID	Human	None	No	None	27.88	3.6
CORE0237.V2.COVID.OP.PRO	Oropharyngeal swab	PRO	CORE0237	V2	COVID	Human	None	No	None	85.48	1.5
CORE0237.V3.COVID.NP.PRO	Nasopharyngeal swab	PRO	CORE0237	V3	COVID	Human	None	No	None	9.01	11.1
CORE0237.V3.COVID.OP.PRO	Oropharyngeal swab	PRO	CORE0237	V3	COVID	Human	None	No	None	0.9	38
CORE0237.V4.COVID.NP.PRO	Nasopharyngeal swab	PRO	CORE0237	V4	COVID	Human	None	No	None	19.61	5.1
CORE0237.V4.COVID.OP.PRO	Oropharyngeal swab	PRO	CORE0237	V4	COVID	Human	None	No	None	56.73	1.8
CORE0237.V5.COVID.NP.PRO	Nasopharyngeal swab	PRO	CORE0237	V5	COVID	Human	None	No	None	2.07	38
CORE0237.V5.COVID.OP.PRO	Oropharyngeal swab	PRO	CORE0237	V5	COVID	Human	None	No	None	40.64	2.5
CORE0237.V6.COVID.NP.PRO	Nasopharyngeal swab	PRO	CORE0237	V6	COVID	Human	None	No	None	23.71	4.2
CORE0237.V6.COVID.OP.PRO	Oropharyngeal swab	PRO	CORE0237	V6	COVID	Human	None	No	None	56.3	1.8
CORE0237.V7.COVID.NP.PRO	Nasopharyngeal swab	PRO	CORE0237	V7	COVID	Human	None	No	None	21.43	4.7
CORE0237.V7.COVID.OP.PRO	Oropharyngeal swab	PRO	CORE0237	V7	COVID	Human	None	No	None	40.14	2.5
CORE0237.V8.COVID.NP.PRO	Nasopharyngeal swab	PRO	CORE0237	V8	COVID	Human	None	No	None	9.92	10.1
CORE0237.V8.COVID.OP.PRO	Oropharyngeal swab	PRO	CORE0237	V8	COVID	Human	None	No	None	20.4	4.9
CORE0237.V9.COVID.NP.PRO	Nasopharyngeal swab	PRO	CORE0237	V9	COVID	Human	None	No	None	34.13	2.9
CORE0237.V9.COVID.OP.PRO	Oropharyngeal swab	PRO	CORE0237	V9	COVID	Human	None	No	None	33.95	3
CORE0240.V1.COVID.NP.PRO	Nasopharyngeal swab	PRO	CORE0240	V1	COVID	Human	None	No	None	46.15	2.2
CORE0240.V2.COVID.NP.PRO	Nasopharyngeal swab	PRO	CORE0240	V2	COVID	Human	None	No	None	52.03	1.9
CORE0240.V2.COVID.OP.PRO	Oropharyngeal swab	PRO	CORE0240	V2	COVID	Human	None	No	None	57.85	1.7
CORE0261.V1.COVID.ETA.PRO	Endotracheal aspirate	PRO	CORE0261	V1	COVID	Human	None	No	23.95	10.86	9.2
CORE0261.V3.COVID.ETA.PRO	Endotracheal aspirate	PRO	CORE0261	V3	COVID	Human	None	No	172.34	28.41	3.5
CORE0261.V4.COVID.ETA.PRO	Endotracheal aspirate	PRO	CORE0261	V4	COVID	Human	None	No	83.15	10.48	9.5
CORE0280.V1.COVID.NP.PRO	Nasopharyngeal swab	PRO	CORE0280	V1	COVID	Human	None	No	None	0.3	36
CORE0280.V1.COVID.OP.PRO	Oropharyngeal swab	PRO	CORE0280	V1	COVID	Human	None	No	None	0.21	36
CORE0296.V1.nonCOVID.NP.PRO	Nasopharyngeal swab	PRO	CORE0296	V1	non COVID	Human	None	No	None	0.15	38
CORE0296.V1.nonCOVID.NP.PS	Nasopharyngeal swab	PS	CORE0296	V1	non COVID	Human	None	No	None	0.4	38
CORE0296.V1.nonCOVID.OP.PRO	Oropharyngeal swab	PRO	CORE0296	V1	non COVID	Human	None	No	None	0.25	38
CORE0296.V1.nonCOVID.OP.PS	Oropharyngeal swab	PS	CORE0296	V1	non COVID	Human	None	No	None	1.18	38
CORE0204.V1.COVID.NP.PRO	Nasopharyngeal swab	PRO	CORE0204	V1	COVID	Human	None	No	17.52	22.65	4.4
CORE0204.V1.COVID.OP.PRO	Oropharyngeal swab	PRO	CORE0204	V1	COVID	Human	None	No	29.04	47.67	2.1
CORE0204.V2.COVID.ETA.PRO	Endotracheal aspirate	PRO	CORE0204	V2	COVID	Human	None	No	None	26.38	3.8
CORE0204.V2.COVID.NP.PRO	Nasopharyngeal swab	PRO	CORE0204	V2	COVID	Human	None	No	91.73	30.14	3.3
CORE0204.V2.COVID.OP.PRO	Oropharyngeal swab	PRO	CORE0204	V2	COVID	Human	None	No	13.65	55.63	1.8
CORE0204.V3.COVID.ETA.PRO	Endotracheal aspirate	PRO	CORE0204	V3	COVID	Human	None	No	None	20.21	5
CORE0204.V3.COVID.NP.PRO	Nasopharyngeal swab	PRO	CORE0204	V3	COVID	Human	None	No	70.5	36.04	2.8
CORE0204.V3.COVID.OP.PRO	Oropharyngeal swab	PRO	CORE0204	V3	COVID	Human	None	No	64.14	38.67	2.6
CORE0204.V4.COVID.NP.PRO	Nasopharyngeal swab	PRO	CORE0204	V4	COVID	Human	None	No	86.97	45.14	2.2
CORE0204.V4.COVID.OP.PRO	Oropharyngeal swab	PRO	CORE0204	V4	COVID	Human	None	No	11.47	46.47	2.2

CORE0204.V5.COVID.NP.PRO	Nasopharyngeal swab	PRO	CORE0204	V5	COVID	Human	None	No	88.44	46.81	2.1
CORE0204.V5.COVID.OP.PRO	Oropharyngeal swab	PRO	CORE0204	V5	COVID	Human	None	No	45.61	67.65	1.5
CORE0180.V1.nonCOVID.NP.PS	Nasopharyngeal swab	PS	CORE0180	V1	non COVID	Human	None	No	None	16.54	6.1
CORE0180.V1.nonCOVID.OP.PS	Oropharyngeal swab	PS	CORE0180	V1	non COVID	Human	None	No	None	19.88	5
CORE0180.V2.nonCOVID.ETA.PRO	Endotracheal aspirate	PRO	CORE0180	V2	non COVID	Human	None	No	None	14.15	7.1
CORE0180.V2.nonCOVID.NP.PS	Nasopharyngeal swab	PS	CORE0180	V2	non COVID	Human	None	No	None	12.67	7.9
CORE0180.V2.nonCOVID.OP.PS	Oropharyngeal swab	PS	CORE0180	V2	non COVID	Human	None	No	None	11.56	8.7
CORE0180.V3.nonCOVID.NP.PS	Nasopharyngeal swab	PS	CORE0180	V3	non COVID	Human	None	No	None	19.07	5.2
CORE0180.V3.nonCOVID.OP.PS	Oropharyngeal swab	PS	CORE0180	V3	non COVID	Human	None	No	None	2.77	36.1
CORE0188.V1.nonCOVID.NP.PS	Nasopharyngeal swab	PS	CORE0188	V1	non COVID	Human	None	No	None	17.51	5.7
CORE0188.V1.nonCOVID.OP.PS	Oropharyngeal swab	PS	CORE0188	V1	non COVID	Human	None	No	None	18.94	5.3
CORE0188.V2.nonCOVID.NP.PS	Nasopharyngeal swab	PS	CORE0188	V2	non COVID	Human	None	No	None	7.71	13
CORE0188.V2.nonCOVID.OP.PS	Oropharyngeal swab	PS	CORE0188	V2	non COVID	Human	None	No	None	15.53	6.4
CORE0188.V3.nonCOVID.NP.PS	Nasopharyngeal swab	PS	CORE0188	V3	non COVID	Human	None	No	None	8.24	12.1
CORE0188.V3.nonCOVID.OP.PS	Oropharyngeal swab	PS	CORE0188	V3	non COVID	Human	None	No	None	0.97	38
CORE0188.V4.nonCOVID.NP.PS	Nasopharyngeal swab	PS	CORE0188	V4	non COVID	Human	None	No	None	11.76	8.5
CORE0188.V4.nonCOVID.OP.PS	Oropharyngeal swab	PS	CORE0188	V4	non COVID	Human	None	No	None	0.07	38
CORE0275.V1.COVID.NP.PRO	Nasopharyngeal swab	PRO	CORE0275	V1	COVID	Human	None	No	None	0.39	36
CORE0275.V1.COVID.OP.PRO	Oropharyngeal swab	PRO	CORE0275	V1	COVID	Human	None	No	None	0.48	36
CORE0275.V2.COVID.NP.PRO	Nasopharyngeal swab	PRO	CORE0275	V2	COVID	Human	None	No	None	58.83	1.7
CORE0275.V2.COVID.OP.PRO	Oropharyngeal swab	PRO	CORE0275	V2	COVID	Human	None	No	None	56.76	1.8
CORE0275.V3.COVID.NP.PRO	Nasopharyngeal swab	PRO	CORE0275	V3	COVID	Human	None	No	None	53.19	1.9
CORE0275.V3.COVID.OP.PRO	Oropharyngeal swab	PRO	CORE0275	V3	COVID	Human	None	No	None	59.35	1.7
CORE0275.V4.COVID.NP.PRO	Nasopharyngeal swab	PRO	CORE0275	V4	COVID	Human	None	No	None	24.52	4.1
CORE0275.V4.COVID.OP.PRO	Oropharyngeal swab	PRO	CORE0275	V4	COVID	Human	None	No	None	61.38	1.6
CORE0282.V1.COVID.ETA.PRO	Endotracheal aspirate	PRO	CORE0282	V1	COVID	Human	None	No	36.59	5.07	19.7
CORE0282.V1.COVID.NP.PRO	Nasopharyngeal swab	PRO	CORE0282	V1	COVID	Human	None	No	None	42.32	2.4
CORE0282.V1.COVID.OP.PRO	Oropharyngeal swab	PRO	CORE0282	V1	COVID	Human	None	No	None	62.88	1.6
CORE0282.V2.COVID.NP.PRO	Nasopharyngeal swab	PRO	CORE0282	V2	COVID	Human	None	No	None	13.86	7.2
CORE0282.V2.COVID.OP.PRO	Oropharyngeal swab	PRO	CORE0282	V2	COVID	Human	None	No	None	10	10
CORE0282.V3.COVID.NP.PRO	Nasopharyngeal swab	PRO	CORE0282	V3	COVID	Human	None	No	None	22.37	4.5
CORE0282.V3.COVID.OP.PRO	Oropharyngeal swab	PRO	CORE0282	V3	COVID	Human	None	No	None	53.89	1.9
CORE0282.V4.COVID.NP.PRO	Nasopharyngeal swab	PRO	CORE0282	V4	COVID	Human	None	No	None	1.49	36
CORE0282.V4.COVID.OP.PRO	Oropharyngeal swab	PRO	CORE0282	V4	COVID	Human	None	No	None	1.38	36
CORE0282.V5.COVID.NP.PRO	Nasopharyngeal swab	PRO	CORE0282	V5	COVID	Human	None	No	None	0.33	36
CORE0282.V5.COVID.OP.PRO	Oropharyngeal swab	PRO	CORE0282	V5	COVID	Human	None	No	None	0.09	38
CORE0294.V1.nonCOVID.NP.PRO	Nasopharyngeal swab	PRO	CORE0294	V1	non COVID	Human	None	No	None	42.81	2.3
CORE0294.V1.nonCOVID.NP.PS	Nasopharyngeal swab	PS	CORE0294	V1	non COVID	Human	None	No	None	49.98	2
CORE0294.V1.nonCOVID.OP.PRO	Oropharyngeal swab	PRO	CORE0294	V1	non COVID	Human	None	No	None	67.8	1.5
CORE0294.V1.nonCOVID.OP.PS	Oropharyngeal swab	PS	CORE0294	V1	non COVID	Human	None	No	None	23.08	4.3
CORE0187.V1.nonCOVID.NP.PS	Nasopharyngeal swab	PS	CORE0187	V1	non COVID	Human	None	No	None	43.44	2.3
CORE0187.V1.nonCOVID.OP.PS	Oropharyngeal swab	PS	CORE0187	V1	non COVID	Human	None	No	None	48.31	2.1
CORE0187.V2.nonCOVID.ETA.PRO	Endotracheal aspirate	PRO	CORE0187	V2	non COVID	Human	None	No	None	31.34	3.2
CORE0187.V2.nonCOVID.NP.PS	Nasopharyngeal swab	PS	CORE0187	V2	non COVID	Human	None	No	None	37.67	2.7
CORE0187.V2.nonCOVID.OP.PS	Oropharyngeal swab	PS	CORE0187	V2	non COVID	Human	None	No	None	38.64	2.6
CORE0187.V3.nonCOVID.NP.PS	Nasopharyngeal swab	PS	CORE0187	V3	non COVID	Human	None	No	None	52.07	1.9
CORE0187.V3.nonCOVID.OP.PS	Oropharyngeal swab	PS	CORE0187	V3	non COVID	Human	None	No	None	50.73	2
CORE0276.V1.COVID.NP.PRO	Nasopharyngeal swab	PRO	CORE0276	V1	COVID	Human	None	No	None	0.18	36
CORE0276.V1.COVID.OP.PRO	Oropharyngeal swab	PRO	CORE0276	V1	COVID	Human	None	No	None	0.14	36
