# Supplementary material for: Signatures of COVID-19 Severity and Immune Response in the Respiratory Tract Microbiome
Source: mBio. 2021 Aug 17;12(4):e01777-21. doi: 10.1128/mBio.01777-21 (PMC8406335; doi:10.1128/mBio.01777-21)
Supplement: TABLE S3 [file mbio.01777-21-st003.pdf]

Table S3. Results of qPCR assays to quantify Anelloviridae and Redondoviridae levels.

SampleID	ttv_ct	ttv_copy	rv_ct	rv_copy
CORE0260.V4.COVID.OP.PRO	Undetermined	0	21.90873019	2404576.604
CORE0196.V3.COVID.OP.PRO	Undetermined	0	24.06678581	220602.0573
CORE0203.V3.COVID.OP.PRO	Undetermined	0	25.17985662	107148.0352
CORE0248.V2.COVID.OP.PRO	Undetermined	0	25.33306758	138491.0026
CORE0221.V1.COVID.OP.PRO	Undetermined	0	25.62192472	73821.08724
CORE0196.V6.COVID.OP.PRO	Undetermined	0	26.17271932	49146.05599
CORE0227.V1.COVID.OP.PRO	Undetermined	0	27.19131152	26067.78337
CORE0211.V1.COVID.OP.PRO	37.38286972	3.392	29.10970561	19764.91677
CORE0204.V1.COVID.OP.PRO	35.03816986	27.777	29.20874977	30286.22571
CORE0248.V3.COVID.OP.PRO	Undetermined	0	29.54088338	13209.88127
CORE0205.V2.COVID.OP.PRO	Undetermined	0	30.5138429	6372.855428
CORE0197.V4.COVID.OP.PRO	Undetermined	0	31.25070572	16232.77297
CORE0203.V4.COVID.OP.PRO	Undetermined	0	31.27158674	1506.519033
CORE0211.V2.COVID.ETA.PRO	Undetermined	0	32.18125343	288.1639938
CORE0203.V2.COVID.OP.PRO	37.55819321	8.0001	32.87889163	388.4609375
CORE0221.V2.COVID.OP.PRO	Undetermined	0	33.20122147	1106.850712
CORE0295.V1.nonCOVID.OP.PS	Undetermined	0	34.62224197	1761.377507
CORE0260.V3.COVID.OP.PRO	Undetermined	0	34.73281924	499.5622937
CORE0203.V2.COVID.ETA.PRO	Undetermined	0	36.11144002	205.7604586
CORE0204.V2.COVID.OP.PRO	Undetermined	0	36.73073387	23.81450915
CORE0227.V2.COVID.OP.PRO	Undetermined	0	37.63503456	54.31451797
CORE0203.V1.COVID.OP.PRO	Undetermined	0	38.39667511	3.127684593
CORE0255.V1.COVID.NP.PRO	8.023150444	2004329344	Undetermined	0
CORE0203.V4.COVID.ETA.PRO	13.27375746	127563812	Undetermined	0
CORE0263.V6.COVID.NP.PRO	24.64973068	27858.68952	Undetermined	0
CORE0263.V6.COVID.ETA.PRO	25.14343071	60431.699	Undetermined	0
CORE0187.V1.nonCOVID.OP.PS	26.52873103	102931.9338	Undetermined	0
CORE0275.V4.COVID.OP.PRO	26.93263817	6056.070426	Undetermined	0
CORE0275.V2.COVID.OP.PRO	27.07812627	6475.126814	Undetermined	0
CORE0275.V3.COVID.OP.PRO	27.36677361	4421.530503	Undetermined	0
CORE0187.V1.nonCOVID.NP.PS	27.58120918	38798.93241	Undetermined	0
CORE0263.V5.COVID.NP.PRO	27.58805021	3795.938492	Undetermined	0
CORE0187.V3.nonCOVID.NP.PS	27.96755981	60916.05491	Undetermined	0
CORE0255.V6.COVID.OP.PRO	28.79712741	2434.560545	Undetermined	0
CORE0275.V3.COVID.NP.PRO	28.85164642	1860.691822	Undetermined	0
CORE0187.V2.nonCOVID.NP.PS	29.15480614	31006.94037	Undetermined	0
CORE0263.V5.COVID.ETA.PRO	29.41250038	5726.687	Undetermined	0
CORE0206.V1.COVID.NP.PRO	29.58557638	1172.667891	Undetermined	0
CORE0181.V4.nonCOVID.OP.PS	29.91299629	2364.552318	Undetermined	0
CORE0206.V3.COVID.ETA.PRO	30.02832413	2139.955688	Undetermined	0
CORE0187.V2.nonCOVID.OP.PS	30.05544345	12118.84686	Undetermined	0
CORE0180.V2.nonCOVID.ETA.PRO	30.24927044	2188.204259	Undetermined	0
CORE0181.V3.nonCOVID.OP.PS	30.32191181	1719.429823	Undetermined	0
CORE0255.V6.COVID.ETA.PRO	30.3742733	1302.367	Undetermined	0
CORE0263.V1.COVID.NP.PRO	30.39976056	604.8591995	Undetermined	0
CORE0210.V1.COVID.ETA.PRO	30.56324005	332.6386108	Undetermined	0
CORE0185.V4.nonCOVID.NP.PS	30.60750008	1000	Undetermined	0
CORE0181.V3.nonCOVID.ETA.PRO	30.69320869	7259.205127	Undetermined	0
CORE0288.V3.COVID.ETA.PRO	30.96118164	1000	Undetermined	0
CORE0180.V1.nonCOVID.NP.PS	31.08769321	1018.096486	Undetermined	0
CORE0263.V5.COVID.OP.PRO	31.09645398	374.8508068	Undetermined	0
CORE0206.V1.COVID.ETA.PRO	31.31845029	1065.522644	Undetermined	0
CORE0237.V3.COVID.NP.PRO	31.4683183	635.7533217	Undetermined	0
CORE0187.V2.nonCOVID.ETA.PRO	31.79879697	4937.957814	Undetermined	0
CORE0263.V2.COVID.OP.PRO	31.82056554	228.9634192	Undetermined	0
CORE0206.V1.COVID.OP.PRO	31.91152382	219.4428009	Undetermined	0
CORE0181.V1.nonCOVID.OP.PS	31.98509502	655.2206453	Undetermined	0
CORE0206.V2.COVID.OP.PRO	32.30992381	159.7716081	Undetermined	0
CORE0263.V3.COVID.OP.PRO	32.43441137	149.5595595	Undetermined	0
CORE0218.V2.COVID.ETA.PRO	32.51799711	178.665	Undetermined	0
CORE0255.V3.COVID.ETA.PRO	32.57891846	262.929	Undetermined	0
CORE0204.V1.COVID.NP.PRO	33.03251648	256.777	Undetermined	0
CORE0251.V4.COVID.OP.PRO	33.47891235	99.56077373	Undetermined	0
CORE0275.V4.COVID.NP.PRO	33.50373713	80.64315659	Undetermined	0
CORE0181.V2.nonCOVID.ETA.PRO	33.60037804	222.8435585	Undetermined	0
CORE0251.V3.COVID.OP.PRO	33.66664569	201.807329	Undetermined	0
CORE0237.V1.COVID.NP.PRO	33.86125692	215.1060396	Undetermined	0
CORE0251.V2.COVID.NP.PRO	33.91192881	64.24984082	Undetermined	0
CORE0180.V1.nonCOVID.OP.PS	34.69527054	94.26752914	Undetermined	0
CORE0263.V3.COVID.NP.PRO	34.72804515	37.42050178	Undetermined	0
CORE0225.V2.COVID.OP.PRO	34.77864456	77.97517014	Undetermined	0
CORE0239.V1.COVID.OP.PRO	34.87983068	59.11471066	Undetermined	0
CORE0207.V2.COVID.NP.PRO	34.94377899	20.852	Undetermined	0
CORE0275.V2.COVID.NP.PRO	35.04739761	27.75885747	Undetermined	0
CORE0252.V6.COVID.OP.PRO	35.05678622	227.7935086	Undetermined	0
CORE0207.V2.COVID.OP.PRO	35.11509323	16.478	Undetermined	0
CORE0263.V1.COVID.OP.PRO	35.48049164	23.00827666	Undetermined	0
CORE0263.V4.COVID.OP.PRO	35.48067729	21.0972061	Undetermined	0
CORE0252.V4.COVID.NP.PRO	35.52054342	21.86799259	Undetermined	0
CORE0255.V5.COVID.OP.PRO	35.61569595	21.76948166	Undetermined	0
CORE0216.V1.COVID.OP.PRO	35.97783852	10.072	Undetermined	0
CORE0252.V5.COVID.ETA.PRO	36.15178299	34.421	Undetermined	0
CORE0261.V1.COVID.ETA.PRO	36.38734627	19.727	Undetermined	0
CORE0212.V1.COVID.OP.PRO	36.53817749	6.889	Undetermined	0
CORE0269.V4.COVID.NP.PRO	36.69234848	9.565162659	Undetermined	0
CORE0263.V1.COVID.ETA.PRO	36.72153727	7.7	Undetermined	0
CORE0255.V5.COVID.ETA.PRO	36.75959015	6.777	Undetermined	0
CORE0213.V3.COVID.OP.PRO	36.78461075	5.147	Undetermined	0
CORE0252.V3.COVID.OP.PRO	36.8374939	9.869452477	Undetermined	0
CORE0205.V1.COVID.NP.PRO	36.84800339	5.649	Undetermined	0
CORE0251.V2.COVID.ETA.PRO	37.07096227	5.685	Undetermined	0
CORE0252.V6.COVID.NP.PRO	37.25024414	7.503951073	Undetermined	0
CORE0251.V1.COVID.NP.PRO	37.29006577	4.941350937	Undetermined	0
CORE0201.V1.COVID.NP.PRO	37.35193253	4.6755	Undetermined	0
CORE0255.V4.COVID.OP.PRO	37.38393021	6.795	Undetermined	0

CORE0225.V5.COVID.NP.PRO	37.38463593	4.158	Undetermined	0
CORE0223.V1.COVID.OP.PRO	37.41020203	7.991751194	Undetermined	0
CORE0212.V4.COVID.ETA.PRO	37.61727524	8.429	Undetermined	0
CORE0245.V1.COVID.NP.PRO	37.62509155	5.792	Undetermined	0
CORE0252.V5.COVID.OP.PRO	37.65855789	5.665	Undetermined	0
CORE0222.V1.COVID.OP.PRO	37.72810364	6.219816147	Undetermined	0
CORE0237.V2.COVID.NP.PRO	37.83757019	6.038749695	Undetermined	0
CORE0242.V4.COVID.NP.PRO	37.90576935	4.809	Undetermined	0
CORE0252.V3.COVID.ETA.PRO	38.00390625	2.754	Undetermined	0
CORE0181.V3.nonCOVID.NP.PS	38.01374435	7.747714043	Undetermined	0
CORE0181.V2.nonCOVID.OP.PS	38.04965401	10.12677384	Undetermined	0
CORE0252.V2.COVID.ETA.PRO	38.09830348	3.682	Undetermined	0
CORE0184.V1.nonCOVID.OP.PS	38.14196396	12.185	Undetermined	0
CORE0180.V2.nonCOVID.OP.PS	38.25907898	12.05943691	Undetermined	0
CORE0218.V1.COVID.OP.PRO	38.34646606	1.733	Undetermined	0
CORE0225.V1.COVID.ETA.PRO	38.3864212	1.777	Undetermined	0
CORE0226.V1.COVID.OP.PRO	38.48120117	3.959773779	Undetermined	0
CORE0276.V1.COVID.OP.PRO	38.5529213	2.741428494	Undetermined	0
CORE0206.V3.COVID.OP.PRO	38.70135498	1.441	Undetermined	0
CORE0230.V2.COVID.NP.PRO	38.85615349	2.226162068	Undetermined	0
CORE0228.V1.COVID.OP.PRO	38.93333054	1.417	Undetermined	0
CORE0262.V2.COVID.OP.PRO	38.97470093	1.563	Undetermined	0
CORE0263.V6.COVID.OP.PRO	39.3656044	1.992993474	Undetermined	0
CORE0252.V3.COVID.NP.PRO	39.52157974	1.033163786	Undetermined	0
CORE0225.V6.COVID.OP.PRO	39.6403923	0.867	Undetermined	0
CORE0255.V4.COVID.NP.PRO	39.67790604	1.497451901	Undetermined	0
CORE0181.V2.nonCOVID.NP.PS	39.71445084	2.445384264	Undetermined	0
CORE0179.V1.nonCOVID.NP.PS	Undetermined	0	Undetermined	0
CORE0179.V1.nonCOVID.OP.PS	Undetermined	0	Undetermined	0
CORE0179.V2.nonCOVID.NP.PS	Undetermined	0	Undetermined	0
CORE0179.V2.nonCOVID.OP.PS	Undetermined	0	Undetermined	0
CORE0180.V3.nonCOVID.NP.PS	Undetermined	0	Undetermined	0
CORE0180.V3.nonCOVID.OP.PS	Undetermined	0	Undetermined	0
CORE0180.V2.nonCOVID.NP.PS	Undetermined	0	Undetermined	0
CORE0181.V4.nonCOVID.NP.PS	Undetermined	0	Undetermined	0
CORE0181.V1.nonCOVID.NP.PS	Undetermined	0	Undetermined	0
CORE0182.V1.nonCOVID.NP.PS	Undetermined	0	Undetermined	0
CORE0182.V1.nonCOVID.OP.PS	Undetermined	0	Undetermined	0
CORE0184.V1.nonCOVID.NP.PS	Undetermined	0	Undetermined	0
CORE0185.V4.nonCOVID.OP.PS	Undetermined	0	Undetermined	0
CORE0185.V1.nonCOVID.NP.PS	Undetermined	0	Undetermined	0
CORE0185.V2.nonCOVID.ETA.PRO	Undetermined	0	Undetermined	0
CORE0185.V1.nonCOVID.OP.PS	Undetermined	0	Undetermined	0
CORE0185.V2.nonCOVID.NP.PS	Undetermined	0	Undetermined	0
CORE0185.V2.nonCOVID.OP.PS	Undetermined	0	Undetermined	0
CORE0185.V3.nonCOVID.NP.PS	Undetermined	0	Undetermined	0
CORE0185.V3.nonCOVID.OP.PS	Undetermined	0	Undetermined	0
CORE0187.V3.nonCOVID.OP.PS	Undetermined	0	Undetermined	0
CORE0188.V1.nonCOVID.NP.PS	Undetermined	0	Undetermined	0
CORE0188.V1.nonCOVID.OP.PS	Undetermined	0	Undetermined	0
CORE0188.V2.nonCOVID.NP.PS	Undetermined	0	Undetermined	0
CORE0188.V2.nonCOVID.OP.PS	Undetermined	0	Undetermined	0
CORE0188.V3.nonCOVID.NP.PS	Undetermined	0	Undetermined	0
CORE0188.V3.nonCOVID.OP.PS	Undetermined	0	Undetermined	0
CORE0188.V4.nonCOVID.NP.PS	Undetermined	0	Undetermined	0
CORE0188.V4.nonCOVID.OP.PS	Undetermined	0	Undetermined	0
CORE0189.V1.nonCOVID.NP.PS	Undetermined	0	Undetermined	0
CORE0189.V4.nonCOVID.OP.PS	Undetermined	0	Undetermined	0
CORE0189.V1.nonCOVID.OP.PS	Undetermined	0	Undetermined	0
CORE0189.V2.nonCOVID.NP.PS	Undetermined	0	Undetermined	0
CORE0189.V2.nonCOVID.OP.PS	Undetermined	0	Undetermined	0
CORE0189.V3.nonCOVID.ETA.PRO	Undetermined	0	Undetermined	0
CORE0189.V4.nonCOVID.ETA.PRO	Undetermined	0	Undetermined	0
CORE0189.V3.nonCOVID.NP.PS	Undetermined	0	Undetermined	0
CORE0189.V3.nonCOVID.OP.PS	Undetermined	0	Undetermined	0
CORE0189.V4.nonCOVID.NP.PS	Undetermined	0	Undetermined	0
CORE0190.V1.nonCOVID.NP.PS	Undetermined	0	Undetermined	0
CORE0190.V1.nonCOVID.OP.PS	Undetermined	0	Undetermined	0
CORE0190.V2.nonCOVID.NP.PS	Undetermined	0	Undetermined	0
CORE0190.V2.nonCOVID.OP.PS	Undetermined	0	Undetermined	0
CORE0294.V1.nonCOVID.NP.PS	Undetermined	0	Undetermined	0
CORE0294.V1.nonCOVID.OP.PS	Undetermined	0	Undetermined	0
CORE0294.V1.nonCOVID.NP.PRO	Undetermined	0	Undetermined	0
CORE0294.V1.nonCOVID.OP.PRO	Undetermined	0	Undetermined	0
CORE0295.V1.nonCOVID.NP.PS	Undetermined	0	Undetermined	0
CORE0295.V1.nonCOVID.NP.PRO	Undetermined	0	Undetermined	0
CORE0295.V1.nonCOVID.OP.PRO	Undetermined	0	Undetermined	0
CORE0296.V1.nonCOVID.NP.PS	Undetermined	0	Undetermined	0
CORE0296.V1.nonCOVID.OP.PS	Undetermined	0	Undetermined	0
CORE0296.V1.nonCOVID.NP.PRO	Undetermined	0	Undetermined	0
CORE0296.V1.nonCOVID.OP.PRO	Undetermined	0	Undetermined	0
CORE0193.V5.COVID.OP.PRO	Undetermined	0	Undetermined	0
CORE0193.V5.COVID.NP.PRO	Undetermined	0	Undetermined	0
CORE0196.V5.COVID.ETA.PRO	Undetermined	0	Undetermined	0
CORE0196.V2.COVID.ETA.PRO	Undetermined	0	Undetermined	0
CORE0196.V3.COVID.ETA.PRO	Undetermined	0	Undetermined	0
CORE0196.V6.COVID.NP.PRO	Undetermined	0	Undetermined	0
CORE0196.V3.COVID.NP.PRO	Undetermined	0	Undetermined	0
CORE0196.V7.COVID.NP.PRO	Undetermined	0	Undetermined	0
CORE0196.V4.COVID.NP.PRO	Undetermined	0	Undetermined	0
CORE0196.V7.COVID.OP.PRO	Undetermined	0	Undetermined	0
CORE0196.V4.COVID.OP.PRO	Undetermined	0	Undetermined	0
CORE0196.V5.COVID.NP.PRO	Undetermined	0	Undetermined	0
CORE0196.V5.COVID.OP.PRO	Undetermined	0	Undetermined	0
CORE0197.V2.COVID.ETA.PRO	Undetermined	0	Undetermined	0
CORE0197.V6.COVID.ETA.PRO	Undetermined	0	Undetermined	0
CORE0197.V5.COVID.NP.PRO	Undetermined	0	Undetermined	0
CORE0197.V5.COVID.OP.PRO	Undetermined	0	Undetermined	0

CORE0197.V6.COVID.NP.PRO	Undetermined	0	Undetermined	0
CORE0197.V3.COVID.NP.PRO	Undetermined	0	Undetermined	0
CORE0197.V6.COVID.OP.PRO	Undetermined	0	Undetermined	0
CORE0197.V3.COVID.OP.PRO	Undetermined	0	Undetermined	0
CORE0197.V4.COVID.NP.PRO	Undetermined	0	Undetermined	0
CORE0200.V1.COVID.NP.PRO	Undetermined	0	Undetermined	0
CORE0200.V1.COVID.OP.PRO	Undetermined	0	Undetermined	0
CORE0201.V1.COVID.OP.PRO	Undetermined	0	Undetermined	0
CORE0202.V1.COVID.NP.PRO	Undetermined	0	Undetermined	0
CORE0202.V1.COVID.OP.PRO	Undetermined	0	Undetermined	0
CORE0203.V1.COVID.ETA.PRO	Undetermined	0	Undetermined	0
CORE0203.V3.COVID.ETA.PRO	Undetermined	0	Undetermined	0
CORE0203.V3.COVID.NP.PRO	Undetermined	0	Undetermined	0
CORE0203.V4.COVID.NP.PRO	Undetermined	0	Undetermined	0
CORE0203.V1.COVID.NP.PRO	Undetermined	0	Undetermined	0
CORE0203.V5.COVID.NP.PRO	Undetermined	0	Undetermined	0
CORE0203.V2.COVID.NP.PRO	Undetermined	0	Undetermined	0
CORE0203.V5.COVID.OP.PRO	Undetermined	0	Undetermined	0
CORE0204.V3.COVID.ETA.PRO	Undetermined	0	Undetermined	0
CORE0204.V2.COVID.ETA.PRO	Undetermined	0	Undetermined	0
CORE0204.V2.COVID.NP.PRO	Undetermined	0	Undetermined	0
CORE0204.V3.COVID.NP.PRO	Undetermined	0	Undetermined	0
CORE0204.V3.COVID.OP.PRO	Undetermined	0	Undetermined	0
CORE0204.V4.COVID.NP.PRO	Undetermined	0	Undetermined	0
CORE0204.V4.COVID.OP.PRO	Undetermined	0	Undetermined	0
CORE0204.V5.COVID.NP.PRO	Undetermined	0	Undetermined	0
CORE0204.V5.COVID.OP.PRO	Undetermined	0	Undetermined	0
CORE0205.V1.COVID.OP.PRO	Undetermined	0	Undetermined	0
CORE0205.V2.COVID.NP.PRO	Undetermined	0	Undetermined	0
CORE0205.V3.COVID.NP.PRO	Undetermined	0	Undetermined	0
CORE0205.V3.COVID.OP.PRO	Undetermined	0	Undetermined	0
CORE0206.V2.COVID.NP.PRO	Undetermined	0	Undetermined	0
CORE0206.V3.COVID.NP.PRO	Undetermined	0	Undetermined	0
CORE0207.V3.COVID.NP.PRO	Undetermined	0	Undetermined	0
CORE0207.V3.COVID.OP.PRO	Undetermined	0	Undetermined	0
CORE0207.V1.COVID.NP.PRO	Undetermined	0	Undetermined	0
CORE0207.V1.COVID.OP.PRO	Undetermined	0	Undetermined	0
CORE0208.V2.COVID.OP.PRO	Undetermined	0	Undetermined	0
CORE0208.V1.COVID.NP.PRO	Undetermined	0	Undetermined	0
CORE0208.V1.COVID.OP.PRO	Undetermined	0	Undetermined	0
CORE0208.V2.COVID.NP.PRO	Undetermined	0	Undetermined	0
CORE0209.V2.COVID.ETA.PRO	Undetermined	0	Undetermined	0
CORE0209.V1.COVID.NP.PRO	Undetermined	0	Undetermined	0
CORE0209.V1.COVID.OP.PRO	Undetermined	0	Undetermined	0
CORE0209.V2.COVID.NP.PRO	Undetermined	0	Undetermined	0
CORE0209.V2.COVID.OP.PRO	Undetermined	0	Undetermined	0
CORE0209.V3.COVID.NP.PRO	Undetermined	0	Undetermined	0
CORE0209.V3.COVID.OP.PRO	Undetermined	0	Undetermined	0
CORE0210.V3.COVID.ETA.PRO	Undetermined	0	Undetermined	0
CORE0210.V5.COVID.ETA.PRO	Undetermined	0	Undetermined	0
CORE0210.V1.COVID.NP.PRO	Undetermined	0	Undetermined	0
CORE0210.V5.COVID.NP.PRO	Undetermined	0	Undetermined	0
CORE0210.V1.COVID.OP.PRO	Undetermined	0	Undetermined	0
CORE0210.V5.COVID.OP.PRO	Undetermined	0	Undetermined	0
CORE0210.V2.COVID.NP.PRO	Undetermined	0	Undetermined	0
CORE0210.V2.COVID.OP.PRO	Undetermined	0	Undetermined	0
CORE0210.V3.COVID.NP.PRO	Undetermined	0	Undetermined	0
CORE0210.V3.COVID.OP.PRO	Undetermined	0	Undetermined	0
CORE0210.V4.COVID.NP.PRO	Undetermined	0	Undetermined	0
CORE0210.V4.COVID.OP.PRO	Undetermined	0	Undetermined	0
CORE0211.V1.COVID.NP.PRO	Undetermined	0	Undetermined	0
CORE0211.V2.COVID.OP.PRO	Undetermined	0	Undetermined	0
CORE0212.V5.COVID.ETA.PRO	Undetermined	0	Undetermined	0
CORE0212.V6.COVID.ETA.PRO	Undetermined	0	Undetermined	0
CORE0212.V5.COVID.OP.PRO	Undetermined	0	Undetermined	0
CORE0212.V2.COVID.OP.PRO	Undetermined	0	Undetermined	0
CORE0212.V6.COVID.NP.PRO	Undetermined	0	Undetermined	0
CORE0212.V3.COVID.NP.PRO	Undetermined	0	Undetermined	0
CORE0212.V6.COVID.OP.PRO	Undetermined	0	Undetermined	0
CORE0212.V3.COVID.OP.PRO	Undetermined	0	Undetermined	0
CORE0212.V1.COVID.NP.PRO	Undetermined	0	Undetermined	0
CORE0212.V4.COVID.NP.PRO	Undetermined	0	Undetermined	0
CORE0212.V4.COVID.OP.PRO	Undetermined	0	Undetermined	0
CORE0212.V2.COVID.NP.PRO	Undetermined	0	Undetermined	0
CORE0212.V5.COVID.NP.PRO	Undetermined	0	Undetermined	0
CORE0213.V3.COVID.ETA.PRO	Undetermined	0	Undetermined	0
CORE0213.V1.COVID.NP.PRO	Undetermined	0	Undetermined	0
CORE0213.V1.COVID.OP.PRO	Undetermined	0	Undetermined	0
CORE0213.V2.COVID.NP.PRO	Undetermined	0	Undetermined	0
CORE0213.V2.COVID.OP.PRO	Undetermined	0	Undetermined	0
CORE0213.V3.COVID.NP.PRO	Undetermined	0	Undetermined	0
CORE0215.V1.COVID.NP.PRO	Undetermined	0	Undetermined	0
CORE0215.V1.COVID.OP.PRO	Undetermined	0	Undetermined	0
CORE0216.V2.COVID.OP.PRO	Undetermined	0	Undetermined	0
CORE0216.V3.COVID.OP.PRO	Undetermined	0	Undetermined	0
CORE0216.V4.COVID.OP.PRO	Undetermined	0	Undetermined	0
CORE0218.V1.COVID.ETA.PRO	Undetermined	0	Undetermined	0
CORE0218.V1.COVID.NP.PRO	Undetermined	0	Undetermined	0
CORE0218.V4.COVID.OP.PRO	Undetermined	0	Undetermined	0
CORE0218.V5.COVID.NP.PRO	Undetermined	0	Undetermined	0
CORE0218.V2.COVID.NP.PRO	Undetermined	0	Undetermined	0
CORE0218.V5.COVID.OP.PRO	Undetermined	0	Undetermined	0
CORE0218.V2.COVID.OP.PRO	Undetermined	0	Undetermined	0
CORE0218.V3.COVID.NP.PRO	Undetermined	0	Undetermined	0
CORE0218.V3.COVID.OP.PRO	Undetermined	0	Undetermined	0
CORE0218.V4.COVID.NP.PRO	Undetermined	0	Undetermined	0
CORE0219.V2.COVID.ETA.PRO	Undetermined	0	Undetermined	0
CORE0219.V3.COVID.OP.PRO	Undetermined	0	Undetermined	0

CORE0219.V1.COVID.NP.PRO	Undetermined	0	Undetermined	0
CORE0219.V1.COVID.OP.PRO	Undetermined	0	Undetermined	0
CORE0219.V2.COVID.NP.PRO	Undetermined	0	Undetermined	0
CORE0219.V2.COVID.OP.PRO	Undetermined	0	Undetermined	0
CORE0219.V3.COVID.NP.PRO	Undetermined	0	Undetermined	0
CORE0220.V1.COVID.NP.PRO	Undetermined	0	Undetermined	0
CORE0220.V1.COVID.OP.PRO	Undetermined	0	Undetermined	0
CORE0221.V1.COVID.NP.PRO	Undetermined	0	Undetermined	0
CORE0221.V2.COVID.NP.PRO	Undetermined	0	Undetermined	0
CORE0222.V1.COVID.NP.PRO	Undetermined	0	Undetermined	0
CORE0222.V4.COVID.OP.PRO	Undetermined	0	Undetermined	0
CORE0222.V2.COVID.NP.PRO	Undetermined	0	Undetermined	0
CORE0222.V2.COVID.OP.PRO	Undetermined	0	Undetermined	0
CORE0222.V3.COVID.NP.PRO	Undetermined	0	Undetermined	0
CORE0222.V3.COVID.OP.PRO	Undetermined	0	Undetermined	0
CORE0222.V4.COVID.NP.PRO	Undetermined	0	Undetermined	0
CORE0223.V2.COVID.OP.PRO	Undetermined	0	Undetermined	0
CORE0224.V1.COVID.OP.PRO	Undetermined	0	Undetermined	0
CORE0225.V4.COVID.ETA.PRO	Undetermined	0	Undetermined	0
CORE0225.V3.COVID.NP.PRO	Undetermined	0	Undetermined	0
CORE0225.V3.COVID.OP.PRO	Undetermined	0	Undetermined	0
CORE0225.V4.COVID.NP.PRO	Undetermined	0	Undetermined	0
CORE0225.V4.COVID.OP.PRO	Undetermined	0	Undetermined	0
CORE0225.V1.COVID.NP.PRO	Undetermined	0	Undetermined	0
CORE0225.V1.COVID.OP.PRO	Undetermined	0	Undetermined	0
CORE0225.V5.COVID.OP.PRO	Undetermined	0	Undetermined	0
CORE0225.V2.COVID.NP.PRO	Undetermined	0	Undetermined	0
CORE0225.V6.COVID.NP.PRO	Undetermined	0	Undetermined	0
CORE0226.V1.COVID.NP.PRO	Undetermined	0	Undetermined	0
CORE0227.V1.COVID.NP.PRO	Undetermined	0	Undetermined	0
CORE0227.V2.COVID.NP.PRO	Undetermined	0	Undetermined	0
CORE0228.V1.COVID.NP.PRO	Undetermined	0	Undetermined	0
CORE0228.V4.COVID.OP.PRO	Undetermined	0	Undetermined	0
CORE0228.V5.COVID.NP.PRO	Undetermined	0	Undetermined	0
CORE0228.V2.COVID.NP.PRO	Undetermined	0	Undetermined	0
CORE0228.V5.COVID.OP.PRO	Undetermined	0	Undetermined	0
CORE0228.V2.COVID.OP.PRO	Undetermined	0	Undetermined	0
CORE0228.V3.COVID.NP.PRO	Undetermined	0	Undetermined	0
CORE0228.V3.COVID.OP.PRO	Undetermined	0	Undetermined	0
CORE0228.V4.COVID.NP.PRO	Undetermined	0	Undetermined	0
CORE0229.V1.COVID.NP.PRO	Undetermined	0	Undetermined	0
CORE0229.V1.COVID.OP.PRO	Undetermined	0	Undetermined	0
CORE0230.V2.COVID.OP.PRO	Undetermined	0	Undetermined	0
CORE0230.V1.COVID.NP.PRO	Undetermined	0	Undetermined	0
CORE0230.V1.COVID.OP.PRO	Undetermined	0	Undetermined	0
CORE0232.V1.COVID.NP.PRO	Undetermined	0	Undetermined	0
CORE0232.V1.COVID.OP.PRO	Undetermined	0	Undetermined	0
CORE0232.V2.COVID.NP.PRO	Undetermined	0	Undetermined	0
CORE0232.V2.COVID.OP.PRO	Undetermined	0	Undetermined	0
CORE0233.V2.COVID.NP.PRO	Undetermined	0	Undetermined	0
CORE0233.V2.COVID.OP.PRO	Undetermined	0	Undetermined	0
CORE0233.V3.COVID.NP.PRO	Undetermined	0	Undetermined	0
CORE0233.V3.COVID.OP.PRO	Undetermined	0	Undetermined	0
CORE0233.V4.COVID.NP.PRO	Undetermined	0	Undetermined	0
CORE0233.V4.COVID.OP.PRO	Undetermined	0	Undetermined	0
CORE0233.V1.COVID.NP.PRO	Undetermined	0	Undetermined	0
CORE0233.V1.COVID.OP.PRO	Undetermined	0	Undetermined	0
CORE0234.V2.COVID.NP.PRO	Undetermined	0	Undetermined	0
CORE0234.V2.COVID.OP.PRO	Undetermined	0	Undetermined	0
CORE0234.V1.COVID.NP.PRO	Undetermined	0	Undetermined	0
CORE0234.V1.COVID.OP.PRO	Undetermined	0	Undetermined	0
CORE0235.V3.COVID.OP.PRO	Undetermined	0	Undetermined	0
CORE0235.V1.COVID.NP.PRO	Undetermined	0	Undetermined	0
CORE0235.V1.COVID.OP.PRO	Undetermined	0	Undetermined	0
CORE0235.V2.COVID.NP.PRO	Undetermined	0	Undetermined	0
CORE0235.V2.COVID.OP.PRO	Undetermined	0	Undetermined	0
CORE0235.V3.COVID.NP.PRO	Undetermined	0	Undetermined	0
CORE0236.V1.COVID.ETA.PRO	Undetermined	0	Undetermined	0
CORE0236.V1.COVID.NP.PRO	Undetermined	0	Undetermined	0
CORE0236.V1.COVID.OP.PRO	Undetermined	0	Undetermined	0
CORE0237.V7.COVID.NP.PRO	Undetermined	0	Undetermined	0
CORE0237.V3.COVID.OP.PRO	Undetermined	0	Undetermined	0
CORE0237.V7.COVID.OP.PRO	Undetermined	0	Undetermined	0
CORE0237.V4.COVID.NP.PRO	Undetermined	0	Undetermined	0
CORE0237.V8.COVID.NP.PRO	Undetermined	0	Undetermined	0
CORE0237.V4.COVID.OP.PRO	Undetermined	0	Undetermined	0
CORE0237.V8.COVID.OP.PRO	Undetermined	0	Undetermined	0
CORE0237.V1.COVID.OP.PRO	Undetermined	0	Undetermined	0
CORE0237.V5.COVID.NP.PRO	Undetermined	0	Undetermined	0
CORE0237.V5.COVID.OP.PRO	Undetermined	0	Undetermined	0
CORE0237.V9.COVID.NP.PRO	Undetermined	0	Undetermined	0
CORE0237.V2.COVID.OP.PRO	Undetermined	0	Undetermined	0
CORE0237.V6.COVID.NP.PRO	Undetermined	0	Undetermined	0
CORE0237.V9.COVID.OP.PRO	Undetermined	0	Undetermined	0
CORE0237.V6.COVID.OP.PRO	Undetermined	0	Undetermined	0
CORE0238.V1.COVID.NP.PRO	Undetermined	0	Undetermined	0
CORE0238.V1.COVID.OP.PRO	Undetermined	0	Undetermined	0
CORE0239.V1.COVID.NP.PRO	Undetermined	0	Undetermined	0
CORE0240.V1.COVID.NP.PRO	Undetermined	0	Undetermined	0
CORE0240.V2.COVID.NP.PRO	Undetermined	0	Undetermined	0
CORE0240.V2.COVID.OP.PRO	Undetermined	0	Undetermined	0
CORE0242.V4.COVID.ETA.PRO	Undetermined	0	Undetermined	0
CORE0242.V5.COVID.ETA.PRO	Undetermined	0	Undetermined	0
CORE0242.V1.COVID.OP.PRO	Undetermined	0	Undetermined	0
CORE0242.V5.COVID.OP.PRO	Undetermined	0	Undetermined	0
CORE0242.V2.COVID.NP.PRO	Undetermined	0	Undetermined	0
CORE0242.V6.COVID.NP.PRO	Undetermined	0	Undetermined	0
CORE0242.V2.COVID.OP.PRO	Undetermined	0	Undetermined	0

CORE0242.V6.COVID.OP.PRO	Undetermined	0	Undetermined	0
CORE0242.V3.COVID.NP.PRO	Undetermined	0	Undetermined	0
CORE0242.V3.COVID.OP.PRO	Undetermined	0	Undetermined	0
CORE0242.V4.COVID.OP.PRO	Undetermined	0	Undetermined	0
CORE0242.V1.COVID.NP.PRO	Undetermined	0	Undetermined	0
CORE0242.V5.COVID.NP.PRO	Undetermined	0	Undetermined	0
CORE0244.V1.COVID.NP.PRO	Undetermined	0	Undetermined	0
CORE0244.V1.COVID.OP.PRO	Undetermined	0	Undetermined	0
CORE0245.V2.COVID.NP.PRO	Undetermined	0	Undetermined	0
CORE0245.V2.COVID.OP.PRO	Undetermined	0	Undetermined	0
CORE0245.V1.COVID.OP.PRO	Undetermined	0	Undetermined	0
CORE0246.V1.COVID.NP.PRO	Undetermined	0	Undetermined	0
CORE0246.V1.COVID.OP.PRO	Undetermined	0	Undetermined	0
CORE0247.V1.COVID.NP.PRO	Undetermined	0	Undetermined	0
CORE0247.V1.COVID.OP.PRO	Undetermined	0	Undetermined	0
CORE0248.V4.COVID.ETA.PRO	Undetermined	0	Undetermined	0
CORE0248.V5.COVID.ETA.PRO	Undetermined	0	Undetermined	0
CORE0248.V6.COVID.ETA.PRO	Undetermined	0	Undetermined	0
CORE0248.V2.COVID.ETA.PRO	Undetermined	0	Undetermined	0
CORE0248.V3.COVID.ETA.PRO	Undetermined	0	Undetermined	0
CORE0248.V1.COVID.OP.PRO	Undetermined	0	Undetermined	0
CORE0248.V5.COVID.NP.PRO	Undetermined	0	Undetermined	0
CORE0248.V2.COVID.NP.PRO	Undetermined	0	Undetermined	0
CORE0248.V5.COVID.OP.PRO	Undetermined	0	Undetermined	0
CORE0248.V6.COVID.NP.PRO	Undetermined	0	Undetermined	0
CORE0248.V6.COVID.OP.PRO	Undetermined	0	Undetermined	0
CORE0248.V3.COVID.NP.PRO	Undetermined	0	Undetermined	0
CORE0248.V4.COVID.NP.PRO	Undetermined	0	Undetermined	0
CORE0248.V1.COVID.NP.PRO	Undetermined	0	Undetermined	0
CORE0248.V4.COVID.OP.PRO	Undetermined	0	Undetermined	0
CORE0251.V3.COVID.NP.PRO	Undetermined	0	Undetermined	0
CORE0251.V4.COVID.NP.PRO	Undetermined	0	Undetermined	0
CORE0251.V1.COVID.OP.PRO	Undetermined	0	Undetermined	0
CORE0251.V2.COVID.OP.PRO	Undetermined	0	Undetermined	0
CORE0252.V2.COVID.OP.PRO	Undetermined	0	Undetermined	0
CORE0252.V4.COVID.OP.PRO	Undetermined	0	Undetermined	0
CORE0252.V1.COVID.NP.PRO	Undetermined	0	Undetermined	0
CORE0252.V5.COVID.NP.PRO	Undetermined	0	Undetermined	0
CORE0252.V1.COVID.OP.PRO	Undetermined	0	Undetermined	0
CORE0252.V2.COVID.NP.PRO	Undetermined	0	Undetermined	0
CORE0253.V1.COVID.NP.PRO	Undetermined	0	Undetermined	0
CORE0253.V1.COVID.OP.PRO	Undetermined	0	Undetermined	0
CORE0254.V1.COVID.NP.PRO	Undetermined	0	Undetermined	0
CORE0254.V1.COVID.OP.PRO	Undetermined	0	Undetermined	0
CORE0254.V2.COVID.NP.PRO	Undetermined	0	Undetermined	0
CORE0254.V2.COVID.OP.PRO	Undetermined	0	Undetermined	0
CORE0255.V1.COVID.OP.PRO	Undetermined	0	Undetermined	0
CORE0255.V5.COVID.NP.PRO	Undetermined	0	Undetermined	0
CORE0255.V2.COVID.NP.PRO	Undetermined	0	Undetermined	0
CORE0255.V2.COVID.OP.PRO	Undetermined	0	Undetermined	0
CORE0255.V6.COVID.NP.PRO	Undetermined	0	Undetermined	0
CORE0255.V3.COVID.NP.PRO	Undetermined	0	Undetermined	0
CORE0255.V3.COVID.OP.PRO	Undetermined	0	Undetermined	0
CORE0255.V7.COVID.OP.PRO	Undetermined	0	Undetermined	0
CORE0255.V7.COVID.NP.PRO	Undetermined	0	Undetermined	0
CORE0256.V1.COVID.ETA.PRO	Undetermined	0	Undetermined	0
CORE0256.V2.COVID.ETA.PRO	Undetermined	0	Undetermined	0
CORE0256.V2.COVID.OP.PRO	Undetermined	0	Undetermined	0
CORE0256.V1.COVID.NP.PRO	Undetermined	0	Undetermined	0
CORE0256.V1.COVID.OP.PRO	Undetermined	0	Undetermined	0
CORE0256.V2.COVID.NP.PRO	Undetermined	0	Undetermined	0
CORE0257.V1.COVID.NP.PRO	Undetermined	0	Undetermined	0
CORE0257.V1.COVID.OP.PRO	Undetermined	0	Undetermined	0
CORE0257.V2.COVID.NP.PRO	Undetermined	0	Undetermined	0
CORE0257.V2.COVID.OP.PRO	Undetermined	0	Undetermined	0
CORE0257.V3.COVID.NP.PRO	Undetermined	0	Undetermined	0
CORE0257.V3.COVID.OP.PRO	Undetermined	0	Undetermined	0
CORE0258.V1.COVID.NP.PRO	Undetermined	0	Undetermined	0
CORE0258.V1.COVID.OP.PRO	Undetermined	0	Undetermined	0
CORE0259.V2.COVID.NP.PRO	Undetermined	0	Undetermined	0
CORE0259.V2.COVID.OP.PRO	Undetermined	0	Undetermined	0
CORE0260.V2.COVID.OP.PRO	Undetermined	0	Undetermined	0
CORE0260.V3.COVID.NP.PRO	Undetermined	0	Undetermined	0
CORE0260.V4.COVID.NP.PRO	Undetermined	0	Undetermined	0
CORE0260.V1.COVID.NP.PRO	Undetermined	0	Undetermined	0
CORE0260.V1.COVID.OP.PRO	Undetermined	0	Undetermined	0
CORE0260.V2.COVID.NP.PRO	Undetermined	0	Undetermined	0
CORE0261.V4.COVID.ETA.PRO	Undetermined	0	Undetermined	0
CORE0261.V3.COVID.ETA.PRO	Undetermined	0	Undetermined	0
CORE0262.V1.COVID.NP.PRO	Undetermined	0	Undetermined	0
CORE0262.V2.COVID.NP.PRO	Undetermined	0	Undetermined	0
CORE0263.V2.COVID.NP.PRO	Undetermined	0	Undetermined	0
CORE0263.V4.COVID.NP.PRO	Undetermined	0	Undetermined	0
CORE0264.V2.COVID.ETA.PRO	Undetermined	0	Undetermined	0
CORE0264.V2.COVID.NP.PRO	Undetermined	0	Undetermined	0
CORE0264.V2.COVID.OP.PRO	Undetermined	0	Undetermined	0
CORE0266.V1.COVID.NP.PRO	Undetermined	0	Undetermined	0
CORE0266.V1.COVID.OP.PRO	Undetermined	0	Undetermined	0
CORE0266.V2.COVID.NP.PRO	Undetermined	0	Undetermined	0
CORE0266.V2.COVID.OP.PRO	Undetermined	0	Undetermined	0
CORE0269.V4.COVID.ETA.PRO	Undetermined	0	Undetermined	0
CORE0269.V2.COVID.ETA.PRO	Undetermined	0	Undetermined	0
CORE0269.V3.COVID.ETA.PRO	Undetermined	0	Undetermined	0
CORE0269.V2.COVID.NP.PRO	Undetermined	0	Undetermined	0
CORE0269.V2.COVID.OP.PRO	Undetermined	0	Undetermined	0
CORE0269.V3.COVID.NP.PRO	Undetermined	0	Undetermined	0
CORE0269.V3.COVID.OP.PRO	Undetermined	0	Undetermined	0
CORE0269.V4.COVID.OP.PRO	Undetermined	0	Undetermined	0

CORE0269.V5.COVID.NP.PRO	Undetermined	0	Undetermined	0
CORE0269.V1.COVID.NP.PRO	Undetermined	0	Undetermined	0
CORE0269.V5.COVID.OP.PRO	Undetermined	0	Undetermined	0
CORE0270.V1.COVID.NP.PRO	Undetermined	0	Undetermined	0
CORE0270.V1.COVID.OP.PRO	Undetermined	0	Undetermined	0
CORE0271.V1.COVID.NP.PRO	Undetermined	0	Undetermined	0
CORE0271.V1.COVID.OP.PRO	Undetermined	0	Undetermined	0
CORE0272.V2.COVID.ETA.PRO	Undetermined	0	Undetermined	0
CORE0272.V5.COVID.ETA.PRO	Undetermined	0	Undetermined	0
CORE0272.V2.COVID.OP.PRO	Undetermined	0	Undetermined	0
CORE0272.V6.COVID.OP.PRO	Undetermined	0	Undetermined	0
CORE0272.V3.COVID.NP.PRO	Undetermined	0	Undetermined	0
CORE0272.V3.COVID.OP.PRO	Undetermined	0	Undetermined	0
CORE0272.V4.COVID.NP.PRO	Undetermined	0	Undetermined	0
CORE0272.V4.COVID.OP.PRO	Undetermined	0	Undetermined	0
CORE0272.V1.COVID.NP.PRO	Undetermined	0	Undetermined	0
CORE0272.V5.COVID.NP.PRO	Undetermined	0	Undetermined	0
CORE0272.V1.COVID.OP.PRO	Undetermined	0	Undetermined	0
CORE0272.V5.COVID.OP.PRO	Undetermined	0	Undetermined	0
CORE0272.V2.COVID.NP.PRO	Undetermined	0	Undetermined	0
CORE0272.V6.COVID.NP.PRO	Undetermined	0	Undetermined	0
CORE0275.V1.COVID.NP.PRO	Undetermined	0	Undetermined	0
CORE0275.V1.COVID.OP.PRO	Undetermined	0	Undetermined	0
CORE0276.V1.COVID.NP.PRO	Undetermined	0	Undetermined	0
CORE0277.V2.COVID.OP.PRO	Undetermined	0	Undetermined	0
CORE0277.V1.COVID.NP.PRO	Undetermined	0	Undetermined	0
CORE0277.V1.COVID.OP.PRO	Undetermined	0	Undetermined	0
CORE0277.V2.COVID.NP.PRO	Undetermined	0	Undetermined	0
CORE0279.V1.COVID.NP.PRO	Undetermined	0	Undetermined	0
CORE0279.V1.COVID.OP.PRO	Undetermined	0	Undetermined	0
CORE0280.V1.COVID.NP.PRO	Undetermined	0	Undetermined	0
CORE0280.V1.COVID.OP.PRO	Undetermined	0	Undetermined	0
CORE0282.V1.COVID.ETA.PRO	Undetermined	0	Undetermined	0
CORE0282.V2.COVID.NP.PRO	Undetermined	0	Undetermined	0
CORE0282.V2.COVID.OP.PRO	Undetermined	0	Undetermined	0
CORE0282.V3.COVID.NP.PRO	Undetermined	0	Undetermined	0
CORE0282.V3.COVID.OP.PRO	Undetermined	0	Undetermined	0
CORE0282.V4.COVID.NP.PRO	Undetermined	0	Undetermined	0
CORE0282.V1.COVID.NP.PRO	Undetermined	0	Undetermined	0
CORE0282.V4.COVID.OP.PRO	Undetermined	0	Undetermined	0
CORE0282.V1.COVID.OP.PRO	Undetermined	0	Undetermined	0
CORE0282.V5.COVID.NP.PRO	Undetermined	0	Undetermined	0
CORE0282.V5.COVID.OP.PRO	Undetermined	0	Undetermined	0
CORE0284.V1.COVID.NP.PRO	Undetermined	0	Undetermined	0
CORE0284.V1.COVID.OP.PRO	Undetermined	0	Undetermined	0
CORE0285.V3.COVID.NP.PRO	Undetermined	0	Undetermined	0
CORE0285.V3.COVID.OP.PRO	Undetermined	0	Undetermined	0
CORE0285.V4.COVID.NP.PRO	Undetermined	0	Undetermined	0
CORE0285.V4.COVID.OP.PRO	Undetermined	0	Undetermined	0
CORE0285.V1.COVID.NP.PRO	Undetermined	0	Undetermined	0
CORE0285.V1.COVID.OP.PRO	Undetermined	0	Undetermined	0
CORE0285.V2.COVID.NP.PRO	Undetermined	0	Undetermined	0
CORE0285.V2.COVID.OP.PRO	Undetermined	0	Undetermined	0
CORE0286.V2.COVID.OP.PRO	Undetermined	0	Undetermined	0
CORE0286.V1.COVID.NP.PRO	Undetermined	0	Undetermined	0
CORE0286.V1.COVID.OP.PRO	Undetermined	0	Undetermined	0
CORE0286.V2.COVID.NP.PRO	Undetermined	0	Undetermined	0
CORE0288.V4.COVID.ETA.PRO	Undetermined	0	Undetermined	0
CORE0288.V5.COVID.ETA.PRO	Undetermined	0	Undetermined	0
CORE0288.V2.COVID.NP.PRO	Undetermined	0	Undetermined	0
CORE0288.V5.COVID.OP.PRO	Undetermined	0	Undetermined	0
CORE0288.V2.COVID.OP.PRO	Undetermined	0	Undetermined	0
CORE0288.V6.COVID.NP.PRO	Undetermined	0	Undetermined	0
CORE0288.V7.COVID.NP.PRO	Undetermined	0	Undetermined	0
CORE0288.V3.COVID.NP.PRO	Undetermined	0	Undetermined	0
CORE0288.V6.COVID.OP.PRO	Undetermined	0	Undetermined	0
CORE0288.V7.COVID.OP.PRO	Undetermined	0	Undetermined	0
CORE0288.V8.COVID.NP.PRO	Undetermined	0	Undetermined	0
CORE0288.V3.COVID.OP.PRO	Undetermined	0	Undetermined	0
CORE0288.V8.COVID.OP.PRO	Undetermined	0	Undetermined	0
CORE0288.V4.COVID.NP.PRO	Undetermined	0	Undetermined	0
CORE0288.V9.COVID.NP.PRO	Undetermined	0	Undetermined	0
CORE0288.V1.COVID.NP.PRO	Undetermined	0	Undetermined	0
CORE0288.V4.COVID.OP.PRO	Undetermined	0	Undetermined	0
CORE0288.V9.COVID.OP.PRO	Undetermined	0	Undetermined	0
CORE0288.V1.COVID.OP.PRO	Undetermined	0	Undetermined	0
CORE0288.V5.COVID.NP.PRO	Undetermined	0	Undetermined	0
CORE0289.V1.COVID.NP.PRO	Undetermined	0	Undetermined	0
CORE0289.V1.COVID.OP.PRO	Undetermined	0	Undetermined	0
CORE0290.V2.COVID.OP.PRO	Undetermined	0	Undetermined	0
CORE0290.V1.COVID.NP.PRO	Undetermined	0	Undetermined	0
CORE0290.V1.COVID.OP.PRO	Undetermined	0	Undetermined	0
CORE0290.V2.COVID.NP.PRO	Undetermined	0	Undetermined	0
CORE0291.V1.COVID.NP.PRO	Undetermined	0	Undetermined	0
CORE0291.V1.COVID.OP.PRO	Undetermined	0	Undetermined	0
CORE0292.V1.COVID.NP.PRO	Undetermined	0	Undetermined	0
CORE0292.V1.COVID.OP.PRO	Undetermined	0	Undetermined	0
CORE0293.V1.COVID.NP.PRO	Undetermined	0	Undetermined	0
CORE0293.V1.COVID.OP.PRO	Undetermined	0	Undetermined	0
CORE0297.V1.COVID.NP.PRO	Undetermined	0	Undetermined	0
CORE0297.V1.COVID.OP.PRO	Undetermined	0	Undetermined	0
CORE0298.V1.COVID.NP.PRO	Undetermined	0	Undetermined	0
CORE0298.V2.COVID.NP.PRO	Undetermined	0	Undetermined	0
CORE0298.V1.COVID.OP.PRO	Undetermined	0	Undetermined	0
CORE0298.V2.COVID.OP.PRO	Undetermined	0	Undetermined	0
