# Supplementary material for: Signatures of COVID-19 Severity and Immune Response in the Respiratory Tract Microbiome
Source: mBio. 2021 Aug 17;12(4):e01777-21. doi: 10.1128/mBio.01777-21 (PMC8406335; doi:10.1128/mBio.01777-21)
Supplement: TABLE S4 [file mbio.01777-21-st004.pdf]

Table S4. Comparison of microbiome data to clinical and immunological data

Table E4A. Comparison of microbiome data to patient demographics, treatment, and outcomes.

	Redondoviridae OP presence/absence	Anelloviridae OP presence/absence	Anelloviridae NP presence/absence	Redondoviridae + Anelloviridae OP presence/absence	Bacterial Diversity OP Simpson index	Bacterial Diversity NP Simpson index	Weighted Unifrac OP PCoA Axis 1	Weighted Unifrac NP PCoA Axis 1	Weighted Unifrac OP PCoA Axis 2	Weighted Unifrac NP PCoA Axis 2	Unweighted Unifrac OP PCoA Axis 1	Unweighted Unifrac NP PCoA Axis 1	Unweighted Unifrac OP PCoA Axis 2	Unweighted Unifrac NP PCoA Axis 2
Age	1.62E-01	9.65E-01	7.68E-01	5.03E-01	7.68E-01	7.68E-01	7.68E-01	7.68E-01	7.68E-01	7.68E-01	7.68E-01	7.68E-01	7.68E-01	7.68E-01
Gender (male/female)	4.94E-01	7.68E-01	6.98E-01	1.00E+00	5.03E-01	1.00E+00	1.00E+00	1.00E+00	1.00E+00	1.00E+00	1.00E+00	1.00E+00	1.00E+00	1.00E+00
BMI	4.85E-01	5.78E-01	3.96E-01	2.73E-01	9.16E-01	5.78E-01	5.78E-01	5.78E-01	5.78E-01	5.78E-01	5.78E-01	5.78E-01	5.78E-01	5.78E-01
Hydroxychloroquine (yes/no)	1.49E-01	1.01E-01	1.00E+00	1.23E-02	5.03E-01	4.85E-01	4.85E-01	4.85E-01	4.85E-01	4.85E-01	4.85E-01	4.85E-01	4.85E-01	4.85E-01
Remdesivir (yes/no)	7.54E-01	1.00E+00	4.85E-01	7.22E-01	1.00E+00	4.85E-01	4.85E-01	4.85E-01	4.85E-01	4.85E-01	4.85E-01	4.85E-01	4.85E-01	4.85E-01
Corticosteroids (yes/no)	1.00E+00	4.98E-01	7.68E-01	4.85E-01	1.01E-01	5.03E-01	5.03E-01	5.03E-01	5.03E-01	5.03E-01	5.03E-01	5.03E-01	5.03E-01	5.03E-01
Chemo immunosupression (yes/no)	1.00E+00	1.00E+00	9.21E-01	7.68E-01	9.65E-01	1.00E+00	1.00E+00	1.00E+00	1.00E+00	1.00E+00	1.00E+00	1.00E+00	1.00E+00	1.00E+00
Intubation (yes/no)	2.02E-02	2.23E-02	5.03E-01	3.67E-04	1.62E-01	4.85E-01	4.85E-01	4.85E-01	4.85E-01	4.85E-01	4.85E-01	4.85E-01	4.85E-01	4.85E-01
WHO score (4-6/7-10)	1.21E-01	5.01E-02	7.68E-01	3.49E-03	2.02E-02	7.68E-01	7.68E-01	7.68E-01	7.68E-01	7.68E-01	7.68E-01	7.68E-01	7.68E-01	7.68E-01
Outcome (live/dead)	1.00E+00	6.96E-01	9.65E-01	8.07E-01	1.88E-01	1.00E+00	1.00E+00	1.00E+00	1.00E+00	1.00E+00	1.00E+00	1.00E+00	1.00E+00	1.00E+00

Table S4B. Comparison of microbiome data to clinical laboratory data.

	Redondoviridae OP presence/absence	Anelloviridae OP presence/absence	Anelloviridae NP presence/absence	Redondoviridae + Anelloviridae OP presence/absence	Bacterial Diversity OP Simpson index	Bacterial Diversity NP Simpson index	Weighted Unifrac OP PCoA Axis 1	Weighted Unifrac NP PCoA Axis 1	Weighted Unifrac OP PCoA Axis 2	Weighted Unifrac NP PCoA Axis 2	Unweighted Unifrac OP PCoA Axis 1	Unweighted Unifrac NP PCoA Axis 1	Unweighted Unifrac OP PCoA Axis 2	Unweighted Unifrac NP PCoA Axis 2
Platelets	8.54E-01	8.77E-01	9.45E-01	8.77E-01	8.77E-01	9.15E-01	9.03E-01	8.77E-01	8.77E-01	8.54E-01	9.29E-01	8.54E-01	8.54E-01	8.54E-01
Procalcitonin	8.54E-01	8.54E-01	8.54E-01	8.54E-01	8.77E-01	9.98E-01	8.94E-01	9.98E-01	8.54E-01	8.62E-01	8.54E-01	8.77E-01	8.54E-01	9.90E-01
C-reactive protein	8.77E-01	9.65E-01	8.62E-01	9.45E-01	9.56E-01	9.65E-01	8.54E-01	8.54E-01	9.65E-01	8.54E-01	8.77E-01	8.54E-01	8.54E-01	9.15E-01
Ferritin	8.54E-01	8.77E-01	8.77E-01	9.15E-01	8.54E-01	8.54E-01	8.77E-01	8.54E-01	9.15E-01	9.90E-01	9.15E-01	8.77E-01	8.77E-01	8.54E-01
D-dimer	8.77E-01	8.54E-01	8.54E-01	8.54E-01	8.54E-01	8.54E-01	8.54E-01	8.54E-01	9.98E-01	8.54E-01	8.54E-01	9.15E-01	8.77E-01	9.90E-01
Troponin-T	8.54E-01	9.45E-01	8.54E-01	8.54E-01	8.54E-01	9.15E-01	4.36E-02	9.98E-01	9.15E-01	8.54E-01	8.77E-01	9.98E-01	8.54E-01	5.36E-01

Table S4C. Comparison of microbiome data to Lymphocyte-to-Neutrophil ratios.

	Redondoviridae OP presence/absence	Anelloviridae OP presence/absence	Anelloviridae NP presence/absence	Redondoviridae + Anelloviridae OP presence/absence	Bacterial Diversity OP Simpson index	Bacterial Diversity NP Simpson index	Weighted Unifrac OP PCoA Axis 1	Weighted Unifrac NP PCoA Axis 1	Weighted Unifrac OP PCoA Axis 2	Weighted Unifrac NP PCoA Axis 2	Unweighted Unifrac OP PCoA Axis 1	Unweighted Unifrac NP PCoA Axis 1	Unweighted Unifrac OP PCoA Axis 2	Unweighted Unifrac NP PCoA Axis 2
Lymphocyte/ Neutrophil ratio	2.30E-01	3.25E-01	7.07E-01	2.22E-01	2.95E-02	3.25E-01	4.57E-01	1.93E-01	1.40E-02	2.30E-01	3.99E-01	4.57E-01	8.40E-03	3.25E-01

Table S4D. Comparison of microbiome data to immune profiling available on 34 subjects.

	Redondoviridae OP presence/absence	Anelloviridae OP presence/absence	Anelloviridae NP presence/absence	Redondoviridae + Anelloviridae OP presence/absence	Bacterial Diversity OP Simpson index	Bacterial Diversity NP Simpson index	Weighted Unifrac OP PCoA Axis 1	Weighted Unifrac NP PCoA Axis 1	Weighted Unifrac OP PCoA Axis 2	Weighted Unifrac NP PCoA Axis 2	Unweighted Unifrac OP PCoA Axis 1	Unweighted Unifrac NP PCoA Axis 1	Unweighted Unifrac OP PCoA Axis 2	Unweighted Unifrac NP PCoA Axis 2
UMAP component1	9.53E-01	7.61E-01	8.26E-01	5.90E-01	5.29E-01	9.53E-01	9.53E-01	9.53E-01	9.53E-01	9.53E-01	9.53E-01	9.53E-01	9.53E-01	9.53E-01
UMAP component2	5.64E-01	9.59E-01	9.15E-01	5.76E-01	6.36E-01	5.90E-01	5.90E-01	5.90E-01	5.90E-01	5.90E-01	5.90E-01	5.90E-01	5.90E-01	5.90E-01
immunotype3	9.95E-01	8.23E-01	9.95E-01	9.68E-01	1.30E-01	7.61E-01	7.61E-01	7.61E-01	7.61E-01	7.61E-01	7.61E-01	7.61E-01	7.61E-01	7.61E-01
percOf_Live_Bcell	7.61E-01	6.45E-01	9.15E-01	5.72E-01	2.56E-01	8.23E-01	8.23E-01	8.23E-01	8.23E-01	8.23E-01	8.23E-01	8.23E-01	8.23E-01	8.23E-01
percOf_Bcell_BcellPB	9.59E-01	9.59E-01	7.70E-01	9.59E-01	7.61E-01	9.59E-01	9.59E-01	9.59E-01	9.59E-01	9.59E-01	9.59E-01	9.59E-01	9.59E-01	9.59E-01
percOf_Live_Tcell	5.72E-01	6.60E-01	7.61E-01	1.79E-01	9.59E-01	9.59E-01	9.59E-01	9.59E-01	9.59E-01	9.59E-01	9.59E-01	9.59E-01	9.59E-01	9.59E-01
percOf_Tcell_CD4	7.97E-01	5.29E-01	1.78E-01	5.29E-01	9.59E-01	5.72E-01	5.72E-01	5.72E-01	5.72E-01	5.72E-01	5.72E-01	5.72E-01	5.72E-01	5.72E-01
percOf_CD4_CD4cTfh	9.53E-01	9.59E-01	9.42E-01	9.53E-01	9.59E-01	9.59E-01	9.59E-01	9.59E-01	9.59E-01	9.59E-01	9.59E-01	9.59E-01	9.59E-01	9.59E-01
percOf_CD8nonNaive_HLADRposCD38p	9.53E-01	9.68E-01	7.61E-01	7.61E-01	1.30E-01	7.61E-01	7.61E-01	7.61E-01	7.61E-01	7.61E-01	7.61E-01	7.61E-01	7.61E-01	7.61E-01
percOf_Tcell_CD8	7.61E-01	5.29E-01	1.78E-01	5.16E-01	9.59E-01	5.29E-01	5.29E-01	5.29E-01	5.29E-01	5.29E-01	5.29E-01	5.29E-01	5.29E-01	5.29E-01
percOf_CD8nonNaive_KI67pos	9.59E-01	7.61E-01	6.12E-01	5.90E-01	2.64E-01	7.61E-01	7.61E-01	7.61E-01	7.61E-01	7.61E-01	7.61E-01	7.61E-01	7.61E-01	7.61E-01
percOf_CD4nonNaive_KI67pos	9.92E-01	7.61E-01	8.23E-01	5.76E-01	6.33E-01	9.59E-01	9.59E-01	9.59E-01	9.59E-01	9.59E-01	9.59E-01	9.59E-01	9.59E-01	9.59E-01
percOf_CD4nonNaive_HLADRposCD38p	9.53E-01	7.11E-01	7.28E-01	5.18E-01	5.72E-01	9.59E-01	9.59E-01	9.59E-01	9.59E-01	9.59E-01	9.59E-01	9.59E-01	9.59E-01	9.59E-01
percOf_CD8_CD8EMRA	8.23E-01	7.61E-01	9.59E-01	5.29E-01	9.59E-01	1.30E-01	1.30E-01	1.30E-01	1.30E-01	1.30E-01	1.30E-01	1.30E-01	1.30E-01	1.30E-01
