# Supplementary material for: Signatures of COVID-19 Severity and Immune Response in the Respiratory Tract Microbiome
Source: mBio. 2021 Aug 17;12(4):e01777-21. doi: 10.1128/mBio.01777-21 (PMC8406335; doi:10.1128/mBio.01777-21)
Supplement: TABLE S5 [file mbio.01777-21-st005.pdf]

Table S5. Synthetic oligonucleotides used in this study.

ID	Sequence (5'-3')	Description
pan-HCRV-AA-Fwd	GCAGAGTTGTCAGCACATTT	Pan-Redondovirus qPCR forward primer
pan-HCRV-AA-Rev	ATACCAGTATAGGAAGATTTCGAG	Pan-Redondovirus qPCR reverse primer
pan-HCRV-AA-Probe	AAATGGAAGGGAGAGAGGCCTTTGG	Pan-Redondovirus qPCR TaqMan probe
TTV_3656_Fwd	AGACTCCGACDTCCYMTTGG	Pan-TTV qPCR forward primer
TTV_3656_Rev	CTYCCVCTGGCBGCYGTGAC	Pan-TTV qPCR reverse primer
TTV_3656_Probe	GAAAGTGAGTGGGGCCAGAC	Pan-TTV qPCR TaqMan probe
V1-Read1-seq	TATGGTAATTGTAGAGTTTGATCCTGGCTCAG	Sequencing Primer
V2-Read2-seq	AGTCAGTCAGCCTGCTGCCTCCCGTAGGAGT	Sequencing Primer
V1V2-Index1-seq	ACTCCTACGGGAGGCAGCAGGCTGACTGACT	Sequencing Primer
V1V2_Forward (27F)	AGAGTTTGATCCTGGCTCAG	Bacterial 16S rRNA gene V1V2 amplification primer, forward
V1V2_Reverse (338R)	TGCTGCCTCCCGTAGGAGT	Bacterial 16S rRNA gene V1V2 amplification primer, reverse
2019-nCoV_N1-F	GACCCCAAAATCAGCGAAAT	SARS-CoV-1 qPCR forward primer
2019-nCoV_N1-R	TCTGGTTACTGCCAGTTGAATCTG	SARS-CoV-1 qPCR reverse primer
2019_nCoV_N1-P	FAM-ACCCCGCATTACGTTTGGTGGACC-IBFQ	SARS-CoV-1 qPCR probe
