# Supplementary material for: Signatures of COVID-19 Severity and Immune Response in the Respiratory Tract Microbiome
Source: mBio. 2021 Aug 17;12(4):e01777-21. doi: 10.1128/mBio.01777-21 (PMC8406335; doi:10.1128/mBio.01777-21)
Supplement: TABLE S6 [file mbio.01777-21-st006.pdf]

Table S6. Accession numbers of sequence data generated in this study.

SampleID	AccessionID	Bioproject_Accession
CORE0179.V1.nonCOVID.NP.PS	SRR13219721	PRJNA683617
CORE0179.V1.nonCOVID.OP.PS	SRR13219720	PRJNA683617
CORE0179.V2.nonCOVID.NP.PS	SRR13219284	PRJNA683617
CORE0179.V2.nonCOVID.OP.PS	SRR13219610	PRJNA683617
CORE0180.V1.nonCOVID.NP.PS	SRR13219258	PRJNA683617
CORE0180.V1.nonCOVID.OP.PS	SRR13219584	PRJNA683617
CORE0180.V2.nonCOVID.ETA.PRO	SRR13219232	PRJNA683617
CORE0180.V2.nonCOVID.NP.PS	SRR13219568	PRJNA683617
CORE0180.V2.nonCOVID.OP.PS	SRR13219554	PRJNA683617
CORE0180.V3.nonCOVID.NP.PS	SRR13219320	PRJNA683617
CORE0180.V3.nonCOVID.OP.PS	SRR13219719	PRJNA683617
CORE0181.V1.nonCOVID.NP.PS	SRR13219652	PRJNA683617
CORE0181.V1.nonCOVID.OP.PS	SRR13219641	PRJNA683617
CORE0181.V2.nonCOVID.ETA.PRO	SRR13219490	PRJNA683617
CORE0181.V2.nonCOVID.NP.PS	SRR13219479	PRJNA683617
CORE0181.V2.nonCOVID.OP.PS	SRR13219468	PRJNA683617
CORE0181.V3.nonCOVID.ETA.PRO	SRR13219402	PRJNA683617
CORE0181.V3.nonCOVID.NP.PS	SRR13219391	PRJNA683617
CORE0181.V3.nonCOVID.OP.PS	SRR13219306	PRJNA683617
CORE0181.V4.nonCOVID.NP.PS	SRR13219295	PRJNA683617
CORE0181.V4.nonCOVID.OP.PS	SRR13219283	PRJNA683617
CORE0182.V1.nonCOVID.NP.PS	SRR13219216	PRJNA683617
CORE0182.V1.nonCOVID.OP.PS	SRR13219205	PRJNA683617
CORE0184.V1.nonCOVID.NP.PS	SRR13219134	PRJNA683617
CORE0184.V1.nonCOVID.OP.PS	SRR13219122	PRJNA683617
CORE0185.V1.nonCOVID.NP.PS	SRR13219814	PRJNA683617
CORE0185.V1.nonCOVID.OP.PS	SRR13219710	PRJNA683617
CORE0185.V2.nonCOVID.ETA.PRO	SRR13219699	PRJNA683617
CORE0185.V2.nonCOVID.NP.PS	SRR13219632	PRJNA683617
CORE0185.V2.nonCOVID.OP.PS	SRR13219621	PRJNA683617
CORE0185.V3.nonCOVID.NP.PS	SRR13219609	PRJNA683617
CORE0185.V3.nonCOVID.OP.PS	SRR13219542	PRJNA683617
CORE0185.V4.nonCOVID.NP.PS	SRR13219531	PRJNA683617
CORE0185.V4.nonCOVID.OP.PS	SRR13219464	PRJNA683617
CORE0187.V1.nonCOVID.NP.PS	SRR13219453	PRJNA683617
CORE0187.V1.nonCOVID.OP.PS	SRR13219442	PRJNA683617
CORE0187.V2.nonCOVID.ETA.PRO	SRR13219376	PRJNA683617
CORE0187.V2.nonCOVID.NP.PS	SRR13219365	PRJNA683617
CORE0187.V2.nonCOVID.OP.PS	SRR13219280	PRJNA683617
CORE0187.V3.nonCOVID.NP.PS	SRR13219269	PRJNA683617
CORE0187.V3.nonCOVID.OP.PS	SRR13219257	PRJNA683617
CORE0188.V1.nonCOVID.NP.PS	SRR13219190	PRJNA683617
CORE0188.V1.nonCOVID.OP.PS	SRR13219179	PRJNA683617
CORE0188.V2.nonCOVID.NP.PS	SRR13219810	PRJNA683617
CORE0188.V2.nonCOVID.OP.PS	SRR13219799	PRJNA683617
CORE0188.V3.nonCOVID.NP.PS	SRR13219788	PRJNA683617
CORE0188.V3.nonCOVID.OP.PS	SRR13219684	PRJNA683617
CORE0188.V4.nonCOVID.NP.PS	SRR13219673	PRJNA683617
CORE0188.V4.nonCOVID.OP.PS	SRR13219606	PRJNA683617
CORE0189.V1.nonCOVID.NP.PS	SRR13219595	PRJNA683617
CORE0189.V1.nonCOVID.OP.PS	SRR13219583	PRJNA683617
CORE0189.V2.nonCOVID.NP.PS	SRR13219516	PRJNA683617
CORE0189.V2.nonCOVID.OP.PS	SRR13219505	PRJNA683617
CORE0189.V3.nonCOVID.ETA.PRO	SRR13219438	PRJNA683617
CORE0189.V3.nonCOVID.NP.PS	SRR13219427	PRJNA683617
CORE0189.V3.nonCOVID.OP.PS	SRR13219417	PRJNA683617
CORE0189.V4.nonCOVID.ETA.PRO	SRR13219350	PRJNA683617
CORE0189.V4.nonCOVID.NP.PS	SRR13219339	PRJNA683617
CORE0189.V4.nonCOVID.OP.PS	SRR13219328	PRJNA683617
CORE0190.V1.nonCOVID.NP.PS	SRR13219243	PRJNA683617
CORE0190.V1.nonCOVID.OP.PS	SRR13219231	PRJNA683617
CORE0190.V2.nonCOVID.NP.PS	SRR13219164	PRJNA683617
CORE0190.V2.nonCOVID.OP.PS	SRR13219153	PRJNA683617
CORE0193.V5.COVID.NP.PRO	SRR13219142	PRJNA683617
CORE0193.V5.COVID.OP.PRO	SRR13219777	PRJNA683617
CORE0196.V2.COVID.ETA.PRO	SRR13219766	PRJNA683617
CORE0196.V3.COVID.ETA.PRO	SRR13219755	PRJNA683617
CORE0196.V3.COVID.NP.PRO	SRR13219744	PRJNA683617
CORE0196.V3.COVID.OP.PRO	SRR13219733	PRJNA683617
CORE0196.V4.COVID.NP.PRO	SRR13219722	PRJNA683617
CORE0196.V4.COVID.OP.PRO	SRR13219567	PRJNA683617
CORE0196.V5.COVID.ETA.PRO	SRR13219563	PRJNA683617
CORE0196.V5.COVID.NP.PRO	SRR13219562	PRJNA683617
CORE0196.V5.COVID.OP.PRO	SRR13219561	PRJNA683617
CORE0196.V6.COVID.NP.PRO	SRR13219560	PRJNA683617
CORE0196.V6.COVID.OP.PRO	SRR13219559	PRJNA683617
CORE0196.V7.COVID.NP.PRO	SRR13219558	PRJNA683617
CORE0196.V7.COVID.OP.PRO	SRR13219557	PRJNA683617
CORE0197.V2.COVID.ETA.PRO	SRR13219556	PRJNA683617
CORE0197.V3.COVID.NP.PRO	SRR13219555	PRJNA683617
CORE0197.V3.COVID.OP.PRO	SRR13219553	PRJNA683617
CORE0197.V4.COVID.NP.PRO	SRR13219552	PRJNA683617
CORE0197.V4.COVID.OP.PRO	SRR13219551	PRJNA683617
CORE0197.V5.COVID.NP.PRO	SRR13219327	PRJNA683617
CORE0197.V5.COVID.OP.PRO	SRR13219326	PRJNA683617
CORE0197.V6.COVID.ETA.PRO	SRR13219325	PRJNA683617
CORE0197.V6.COVID.NP.PRO	SRR13219324	PRJNA683617
CORE0197.V6.COVID.OP.PRO	SRR13219323	PRJNA683617
CORE0200.V1.COVID.NP.PRO	SRR13219322	PRJNA683617
CORE0200.V1.COVID.OP.PRO	SRR13219321	PRJNA683617
CORE0201.V1.COVID.NP.PRO	SRR13219319	PRJNA683617
CORE0201.V1.COVID.OP.PRO	SRR13219318	PRJNA683617
CORE0202.V1.COVID.NP.PRO	SRR13219317	PRJNA683617
CORE0202.V1.COVID.OP.PRO	SRR13219316	PRJNA683617
CORE0203.V1.COVID.ETA.PRO	SRR13219315	PRJNA683617
CORE0203.V1.COVID.NP.PRO	SRR13219314	PRJNA683617
CORE0203.V1.COVID.OP.PRO	SRR13219313	PRJNA683617
CORE0203.V2.COVID.ETA.PRO	SRR13219312	PRJNA683617
CORE0203.V2.COVID.NP.PRO	SRR13219311	PRJNA683617
CORE0203.V2.COVID.OP.PRO	SRR13219309	PRJNA683617
CORE0203.V3.COVID.ETA.PRO	SRR13219662	PRJNA683617
CORE0203.V3.COVID.NP.PRO	SRR13219661	PRJNA683617
CORE0203.V3.COVID.OP.PRO	SRR13219660	PRJNA683617
CORE0203.V4.COVID.ETA.PRO	SRR13219659	PRJNA683617
CORE0203.V4.COVID.NP.PRO	SRR13219658	PRJNA683617
CORE0203.V4.COVID.OP.PRO	SRR13219657	PRJNA683617
CORE0203.V5.COVID.NP.PRO	SRR13219656	PRJNA683617
CORE0203.V5.COVID.OP.PRO	SRR13219655	PRJNA683617
CORE0204.V1.COVID.NP.PRO	SRR13219654	PRJNA683617
CORE0204.V1.COVID.OP.PRO	SRR13219653	PRJNA683617
CORE0204.V2.COVID.ETA.PRO	SRR13219651	PRJNA683617
CORE0204.V2.COVID.NP.PRO	SRR13219650	PRJNA683617
CORE0204.V2.COVID.OP.PRO	SRR13219649	PRJNA683617
CORE0204.V3.COVID.ETA.PRO	SRR13219648	PRJNA683617
CORE0204.V3.COVID.NP.PRO	SRR13219647	PRJNA683617
CORE0204.V3.COVID.OP.PRO	SRR13219646	PRJNA683617
CORE0204.V4.COVID.NP.PRO	SRR13219645	PRJNA683617
CORE0204.V4.COVID.OP.PRO	SRR13219644	PRJNA683617
CORE0204.V5.COVID.NP.PRO	SRR13219643	PRJNA683617
CORE0204.V5.COVID.OP.PRO	SRR13219642	PRJNA683617
CORE0205.V1.COVID.NP.PRO	SRR13219640	PRJNA683617
CORE0205.V1.COVID.OP.PRO	SRR13219639	PRJNA683617
CORE0205.V2.COVID.NP.PRO	SRR13219638	PRJNA683617
CORE0205.V2.COVID.OP.PRO	SRR13219637	PRJNA683617
CORE0205.V3.COVID.NP.PRO	SRR13219636	PRJNA683617
CORE0205.V3.COVID.OP.PRO	SRR13219635	PRJNA683617
CORE0206.V1.COVID.ETA.PRO	SRR13219494	PRJNA683617
CORE0206.V1.COVID.NP.PRO	SRR13219493	PRJNA683617
CORE0206.V1.COVID.OP.PRO	SRR13219492	PRJNA683617
CORE0206.V2.COVID.NP.PRO	SRR13219491	PRJNA683617
CORE0206.V2.COVID.OP.PRO	SRR13219489	PRJNA683617
CORE0206.V3.COVID.ETA.PRO	SRR13219488	PRJNA683617
CORE0206.V3.COVID.NP.PRO	SRR13219487	PRJNA683617
CORE0206.V3.COVID.OP.PRO	SRR13219486	PRJNA683617
CORE0207.V1.COVID.NP.PRO	SRR13219485	PRJNA683617
CORE0207.V1.COVID.OP.PRO	SRR13219484	PRJNA683617
CORE0207.V2.COVID.NP.PRO	SRR13219483	PRJNA683617
CORE0207.V2.COVID.OP.PRO	SRR13219482	PRJNA683617
CORE0207.V3.COVID.NP.PRO	SRR13219481	PRJNA683617
CORE0207.V3.COVID.OP.PRO	SRR13219480	PRJNA683617
CORE0208.V1.COVID.NP.PRO	SRR13219478	PRJNA683617
CORE0208.V1.COVID.OP.PRO	SRR13219477	PRJNA683617
CORE0208.V2.COVID.NP.PRO	SRR13219476	PRJNA683617
CORE0208.V2.COVID.OP.PRO	SRR13219475	PRJNA683617
CORE0209.V1.COVID.NP.PRO	SRR13219474	PRJNA683617
CORE0209.V1.COVID.OP.PRO	SRR13219473	PRJNA683617
CORE0209.V2.COVID.ETA.PRO	SRR13219472	PRJNA683617
CORE0209.V2.COVID.NP.PRO	SRR13219471	PRJNA683617
CORE0209.V2.COVID.OP.PRO	SRR13219470	PRJNA683617
CORE0209.V3.COVID.NP.PRO	SRR13219469	PRJNA683617
CORE0209.V3.COVID.OP.PRO	SRR13219467	PRJNA683617
CORE0210.V1.COVID.ETA.PRO	SRR13219411	PRJNA683617
CORE0210.V1.COVID.NP.PRO	SRR13219410	PRJNA683617
CORE0210.V1.COVID.OP.PRO	SRR13219409	PRJNA683617
CORE0210.V2.COVID.NP.PRO	SRR13219408	PRJNA683617
CORE0210.V2.COVID.OP.PRO	SRR13219407	PRJNA683617
CORE0210.V3.COVID.ETA.PRO	SRR13219406	PRJNA683617
CORE0210.V3.COVID.NP.PRO	SRR13219405	PRJNA683617
CORE0210.V3.COVID.OP.PRO	SRR13219404	PRJNA683617
CORE0210.V4.COVID.NP.PRO	SRR13219403	PRJNA683617

CORE0210.V4.COVID.OP.PRO	SRR13219401	PRJNA683617
CORE0210.V5.COVID.ETA.PRO	SRR13219400	PRJNA683617
CORE0210.V5.COVID.NP.PRO	SRR13219399	PRJNA683617
CORE0210.V5.COVID.OP.PRO	SRR13219398	PRJNA683617
CORE0211.V1.COVID.NP.PRO	SRR13219397	PRJNA683617
CORE0211.V1.COVID.OP.PRO	SRR13219396	PRJNA683617
CORE0211.V2.COVID.ETA.PRO	SRR13219395	PRJNA683617
CORE0211.V2.COVID.OP.PRO	SRR13219394	PRJNA683617
CORE0212.V1.COVID.NP.PRO	SRR13219393	PRJNA683617
CORE0212.V1.COVID.OP.PRO	SRR13219392	PRJNA683617
CORE0212.V2.COVID.NP.PRO	SRR13219390	PRJNA683617
CORE0212.V2.COVID.OP.PRO	SRR13219389	PRJNA683617
CORE0212.V3.COVID.NP.PRO	SRR13219388	PRJNA683617
CORE0212.V3.COVID.OP.PRO	SRR13219387	PRJNA683617
CORE0212.V4.COVID.ETA.PRO	SRR13219386	PRJNA683617
CORE0212.V4.COVID.NP.PRO	SRR13219385	PRJNA683617
CORE0212.V4.COVID.OP.PRO	SRR13219384	PRJNA683617
CORE0212.V5.COVID.ETA.PRO	SRR13219310	PRJNA683617
CORE0212.V5.COVID.NP.PRO	SRR13219308	PRJNA683617
CORE0212.V5.COVID.OP.PRO	SRR13219307	PRJNA683617
CORE0212.V6.COVID.ETA.PRO	SRR13219305	PRJNA683617
CORE0212.V6.COVID.NP.PRO	SRR13219304	PRJNA683617
CORE0212.V6.COVID.OP.PRO	SRR13219303	PRJNA683617
CORE0213.V1.COVID.NP.PRO	SRR13219302	PRJNA683617
CORE0213.V1.COVID.OP.PRO	SRR13219301	PRJNA683617
CORE0213.V2.COVID.NP.PRO	SRR13219300	PRJNA683617
CORE0213.V2.COVID.OP.PRO	SRR13219299	PRJNA683617
CORE0213.V3.COVID.ETA.PRO	SRR13219298	PRJNA683617
CORE0213.V3.COVID.NP.PRO	SRR13219297	PRJNA683617
CORE0213.V3.COVID.OP.PRO	SRR13219296	PRJNA683617
CORE0215.V1.COVID.NP.PRO	SRR13219294	PRJNA683617
CORE0215.V1.COVID.OP.PRO	SRR13219293	PRJNA683617
CORE0216.V1.COVID.OP.PRO	SRR13219292	PRJNA683617
CORE0216.V2.COVID.OP.PRO	SRR13219291	PRJNA683617
CORE0216.V3.COVID.OP.PRO	SRR13219290	PRJNA683617
CORE0216.V4.COVID.OP.PRO	SRR13219289	PRJNA683617
CORE0218.V1.COVID.ETA.PRO	SRR13219288	PRJNA683617
CORE0218.V1.COVID.NP.PRO	SRR13219287	PRJNA683617
CORE0218.V1.COVID.OP.PRO	SRR13219286	PRJNA683617
CORE0218.V2.COVID.ETA.PRO	SRR13219285	PRJNA683617
CORE0218.V2.COVID.NP.PRO	SRR13219282	PRJNA683617
CORE0218.V2.COVID.OP.PRO	SRR13219225	PRJNA683617
CORE0218.V3.COVID.NP.PRO	SRR13219224	PRJNA683617
CORE0218.V3.COVID.OP.PRO	SRR13219223	PRJNA683617
CORE0218.V4.COVID.NP.PRO	SRR13219222	PRJNA683617
CORE0218.V4.COVID.OP.PRO	SRR13219221	PRJNA683617
CORE0218.V5.COVID.NP.PRO	SRR13219220	PRJNA683617
CORE0218.V5.COVID.OP.PRO	SRR13219219	PRJNA683617
CORE0219.V1.COVID.NP.PRO	SRR13219218	PRJNA683617
CORE0219.V1.COVID.OP.PRO	SRR13219217	PRJNA683617
CORE0219.V2.COVID.ETA.PRO	SRR13219215	PRJNA683617
CORE0219.V2.COVID.NP.PRO	SRR13219214	PRJNA683617
CORE0219.V2.COVID.OP.PRO	SRR13219213	PRJNA683617
CORE0219.V3.COVID.NP.PRO	SRR13219212	PRJNA683617
CORE0219.V3.COVID.OP.PRO	SRR13219211	PRJNA683617
CORE0220.V1.COVID.NP.PRO	SRR13219210	PRJNA683617
CORE0220.V1.COVID.OP.PRO	SRR13219209	PRJNA683617
CORE0221.V1.COVID.NP.PRO	SRR13219208	PRJNA683617
CORE0221.V1.COVID.OP.PRO	SRR13219207	PRJNA683617
CORE0221.V2.COVID.NP.PRO	SRR13219206	PRJNA683617
CORE0221.V2.COVID.OP.PRO	SRR13219204	PRJNA683617
CORE0222.V1.COVID.NP.PRO	SRR13219203	PRJNA683617
CORE0222.V1.COVID.OP.PRO	SRR13219202	PRJNA683617
CORE0222.V2.COVID.NP.PRO	SRR13219201	PRJNA683617
CORE0222.V2.COVID.OP.PRO	SRR13219200	PRJNA683617
CORE0222.V3.COVID.NP.PRO	SRR13219199	PRJNA683617
CORE0222.V3.COVID.OP.PRO	SRR13219198	PRJNA683617
CORE0222.V4.COVID.NP.PRO	SRR13219138	PRJNA683617
CORE0222.V4.COVID.OP.PRO	SRR13219136	PRJNA683617
CORE0223.V1.COVID.OP.PRO	SRR13219135	PRJNA683617
CORE0223.V2.COVID.OP.PRO	SRR13219133	PRJNA683617
CORE0224.V1.COVID.OP.PRO	SRR13219132	PRJNA683617
CORE0225.V1.COVID.ETA.PRO	SRR13219131	PRJNA683617
CORE0225.V1.COVID.NP.PRO	SRR13219130	PRJNA683617
CORE0225.V1.COVID.OP.PRO	SRR13219129	PRJNA683617
CORE0225.V2.COVID.NP.PRO	SRR13219128	PRJNA683617
CORE0225.V2.COVID.OP.PRO	SRR13219127	PRJNA683617
CORE0225.V3.COVID.NP.PRO	SRR13219125	PRJNA683617
CORE0225.V3.COVID.OP.PRO	SRR13219124	PRJNA683617
CORE0225.V4.COVID.ETA.PRO	SRR13219123	PRJNA683617
CORE0225.V4.COVID.NP.PRO	SRR13219824	PRJNA683617
CORE0225.V4.COVID.OP.PRO	SRR13219823	PRJNA683617
CORE0225.V5.COVID.NP.PRO	SRR13219822	PRJNA683617
CORE0225.V5.COVID.OP.PRO	SRR13219821	PRJNA683617
CORE0225.V6.COVID.NP.PRO	SRR13219820	PRJNA683617
CORE0225.V6.COVID.OP.PRO	SRR13219819	PRJNA683617
CORE0226.V1.COVID.NP.PRO	SRR13219818	PRJNA683617
CORE0226.V1.COVID.OP.PRO	SRR13219817	PRJNA683617
CORE0227.V1.COVID.NP.PRO	SRR13219816	PRJNA683617
CORE0227.V1.COVID.OP.PRO	SRR13219815	PRJNA683617
CORE0227.V2.COVID.NP.PRO	SRR13219813	PRJNA683617
CORE0227.V2.COVID.OP.PRO	SRR13219812	PRJNA683617
CORE0228.V1.COVID.NP.PRO	SRR13219718	PRJNA683617
CORE0228.V1.COVID.OP.PRO	SRR13219717	PRJNA683617
CORE0228.V2.COVID.NP.PRO	SRR13219716	PRJNA683617
CORE0228.V2.COVID.OP.PRO	SRR13219715	PRJNA683617
CORE0228.V3.COVID.NP.PRO	SRR13219714	PRJNA683617
CORE0228.V3.COVID.OP.PRO	SRR13219713	PRJNA683617
CORE0228.V4.COVID.NP.PRO	SRR13219712	PRJNA683617
CORE0228.V4.COVID.OP.PRO	SRR13219711	PRJNA683617
CORE0228.V5.COVID.NP.PRO	SRR13219709	PRJNA683617
CORE0228.V5.COVID.OP.PRO	SRR13219708	PRJNA683617
CORE0229.V1.COVID.NP.PRO	SRR13219707	PRJNA683617
CORE0229.V1.COVID.OP.PRO	SRR13219706	PRJNA683617
CORE0230.V1.COVID.NP.PRO	SRR13219705	PRJNA683617
CORE0230.V1.COVID.OP.PRO	SRR13219704	PRJNA683617
CORE0230.V2.COVID.NP.PRO	SRR13219703	PRJNA683617
CORE0230.V2.COVID.OP.PRO	SRR13219702	PRJNA683617
CORE0232.V1.COVID.NP.PRO	SRR13219701	PRJNA683617
CORE0232.V1.COVID.OP.PRO	SRR13219700	PRJNA683617
CORE0232.V2.COVID.NP.PRO	SRR13219698	PRJNA683617
CORE0232.V2.COVID.OP.PRO	SRR13219697	PRJNA683617
CORE0233.V1.COVID.NP.PRO	SRR13219696	PRJNA683617
CORE0233.V1.COVID.OP.PRO	SRR13219695	PRJNA683617
CORE0233.V2.COVID.NP.PRO	SRR13219694	PRJNA683617
CORE0233.V2.COVID.OP.PRO	SRR13219693	PRJNA683617
CORE0233.V3.COVID.NP.PRO	SRR13219692	PRJNA683617
CORE0233.V3.COVID.OP.PRO	SRR13219691	PRJNA683617
CORE0233.V4.COVID.NP.PRO	SRR13219634	PRJNA683617
CORE0233.V4.COVID.OP.PRO	SRR13219633	PRJNA683617
CORE0234.V1.COVID.NP.PRO	SRR13219631	PRJNA683617
CORE0234.V1.COVID.OP.PRO	SRR13219630	PRJNA683617
CORE0234.V2.COVID.NP.PRO	SRR13219629	PRJNA683617
CORE0234.V2.COVID.OP.PRO	SRR13219628	PRJNA683617
CORE0235.V1.COVID.NP.PRO	SRR13219627	PRJNA683617
CORE0235.V1.COVID.OP.PRO	SRR13219626	PRJNA683617
CORE0235.V2.COVID.NP.PRO	SRR13219625	PRJNA683617
CORE0235.V2.COVID.OP.PRO	SRR13219624	PRJNA683617
CORE0235.V3.COVID.NP.PRO	SRR13219623	PRJNA683617
CORE0235.V3.COVID.OP.PRO	SRR13219622	PRJNA683617
CORE0236.V1.COVID.ETA.PRO	SRR13219620	PRJNA683617
CORE0236.V1.COVID.NP.PRO	SRR13219619	PRJNA683617
CORE0236.V1.COVID.OP.PRO	SRR13219618	PRJNA683617
CORE0237.V1.COVID.NP.PRO	SRR13219617	PRJNA683617
CORE0237.V1.COVID.OP.PRO	SRR13219616	PRJNA683617
CORE0237.V2.COVID.NP.PRO	SRR13219615	PRJNA683617
CORE0237.V2.COVID.OP.PRO	SRR13219614	PRJNA683617
CORE0237.V3.COVID.NP.PRO	SRR13219613	PRJNA683617
CORE0237.V3.COVID.OP.PRO	SRR13219612	PRJNA683617
CORE0237.V4.COVID.NP.PRO	SRR13219611	PRJNA683617
CORE0237.V4.COVID.OP.PRO	SRR13219608	PRJNA683617
CORE0237.V5.COVID.NP.PRO	SRR13219607	PRJNA683617
CORE0237.V5.COVID.OP.PRO	SRR13219550	PRJNA683617
CORE0237.V6.COVID.NP.PRO	SRR13219549	PRJNA683617
CORE0237.V6.COVID.OP.PRO	SRR13219548	PRJNA683617
CORE0237.V7.COVID.NP.PRO	SRR13219547	PRJNA683617
CORE0237.V7.COVID.OP.PRO	SRR13219546	PRJNA683617
CORE0237.V8.COVID.NP.PRO	SRR13219545	PRJNA683617
CORE0237.V8.COVID.OP.PRO	SRR13219544	PRJNA683617
CORE0237.V9.COVID.NP.PRO	SRR13219543	PRJNA683617
CORE0237.V9.COVID.OP.PRO	SRR13219541	PRJNA683617
CORE0238.V1.COVID.NP.PRO	SRR13219540	PRJNA683617
CORE0238.V1.COVID.OP.PRO	SRR13219539	PRJNA683617
CORE0239.V1.COVID.NP.PRO	SRR13219538	PRJNA683617
CORE0239.V1.COVID.OP.PRO	SRR13219537	PRJNA683617
CORE0240.V1.COVID.NP.PRO	SRR13219536	PRJNA683617
CORE0240.V2.COVID.NP.PRO	SRR13219535	PRJNA683617
CORE0240.V2.COVID.OP.PRO	SRR13219534	PRJNA683617
CORE0242.V1.COVID.NP.PRO	SRR13219533	PRJNA683617
CORE0242.V1.COVID.OP.PRO	SRR13219532	PRJNA683617
CORE0242.V2.COVID.NP.PRO	SRR13219530	PRJNA683617
CORE0242.V2.COVID.OP.PRO	SRR13219529	PRJNA683617

CORE0242.V3.COVID.NP.PRO	SRR13219528	PRJNA683617
CORE0242.V3.COVID.OP.PRO	SRR13219527	PRJNA683617
CORE0242.V4.COVID.ETA.PRO	SRR13219526	PRJNA683617
CORE0242.V4.COVID.NP.PRO	SRR13219525	PRJNA683617
CORE0242.V4.COVID.OP.PRO	SRR13219524	PRJNA683617
CORE0242.V5.COVID.ETA.PRO	SRR13219523	PRJNA683617
CORE0242.V5.COVID.NP.PRO	SRR13219466	PRJNA683617
CORE0242.V5.COVID.OP.PRO	SRR13219465	PRJNA683617
CORE0242.V6.COVID.NP.PRO	SRR13219463	PRJNA683617
CORE0242.V6.COVID.OP.PRO	SRR13219462	PRJNA683617
CORE0244.V1.COVID.NP.PRO	SRR13219461	PRJNA683617
CORE0244.V1.COVID.OP.PRO	SRR13219460	PRJNA683617
CORE0245.V1.COVID.NP.PRO	SRR13219459	PRJNA683617
CORE0245.V1.COVID.OP.PRO	SRR13219458	PRJNA683617
CORE0245.V2.COVID.NP.PRO	SRR13219457	PRJNA683617
CORE0245.V2.COVID.OP.PRO	SRR13219456	PRJNA683617
CORE0246.V1.COVID.NP.PRO	SRR13219455	PRJNA683617
CORE0246.V1.COVID.OP.PRO	SRR13219454	PRJNA683617
CORE0247.V1.COVID.NP.PRO	SRR13219452	PRJNA683617
CORE0247.V1.COVID.OP.PRO	SRR13219451	PRJNA683617
CORE0248.V1.COVID.NP.PRO	SRR13219450	PRJNA683617
CORE0248.V1.COVID.OP.PRO	SRR13219449	PRJNA683617
CORE0248.V2.COVID.ETA.PRO	SRR13219448	PRJNA683617
CORE0248.V2.COVID.NP.PRO	SRR13219447	PRJNA683617
CORE0248.V2.COVID.OP.PRO	SRR13219446	PRJNA683617
CORE0248.V3.COVID.ETA.PRO	SRR13219445	PRJNA683617
CORE0248.V3.COVID.NP.PRO	SRR13219444	PRJNA683617
CORE0248.V3.COVID.OP.PRO	SRR13219443	PRJNA683617
CORE0248.V4.COVID.ETA.PRO	SRR13219441	PRJNA683617
CORE0248.V4.COVID.NP.PRO	SRR13219440	PRJNA683617
CORE0248.V4.COVID.OP.PRO	SRR13219439	PRJNA683617
CORE0248.V5.COVID.ETA.PRO	SRR13219383	PRJNA683617
CORE0248.V5.COVID.NP.PRO	SRR13219382	PRJNA683617
CORE0248.V5.COVID.OP.PRO	SRR13219381	PRJNA683617
CORE0248.V6.COVID.ETA.PRO	SRR13219380	PRJNA683617
CORE0248.V6.COVID.NP.PRO	SRR13219379	PRJNA683617
CORE0248.V6.COVID.OP.PRO	SRR13219378	PRJNA683617
CORE0251.V1.COVID.NP.PRO	SRR13219377	PRJNA683617
CORE0251.V1.COVID.OP.PRO	SRR13219375	PRJNA683617
CORE0251.V2.COVID.ETA.PRO	SRR13219374	PRJNA683617
CORE0251.V2.COVID.NP.PRO	SRR13219373	PRJNA683617
CORE0251.V2.COVID.OP.PRO	SRR13219372	PRJNA683617
CORE0251.V3.COVID.NP.PRO	SRR13219371	PRJNA683617
CORE0251.V3.COVID.OP.PRO	SRR13219370	PRJNA683617
CORE0251.V4.COVID.NP.PRO	SRR13219369	PRJNA683617
CORE0251.V4.COVID.OP.PRO	SRR13219368	PRJNA683617
CORE0252.V1.COVID.NP.PRO	SRR13219367	PRJNA683617
CORE0252.V1.COVID.OP.PRO	SRR13219366	PRJNA683617
CORE0252.V2.COVID.ETA.PRO	SRR13219364	PRJNA683617
CORE0252.V2.COVID.NP.PRO	SRR13219363	PRJNA683617
CORE0252.V2.COVID.OP.PRO	SRR13219362	PRJNA683617
CORE0252.V3.COVID.ETA.PRO	SRR13219361	PRJNA683617
CORE0252.V3.COVID.NP.PRO	SRR13219360	PRJNA683617
CORE0252.V3.COVID.OP.PRO	SRR13219359	PRJNA683617
CORE0252.V4.COVID.NP.PRO	SRR13219358	PRJNA683617
CORE0252.V4.COVID.OP.PRO	SRR13219357	PRJNA683617
CORE0252.V5.COVID.ETA.PRO	SRR13219356	PRJNA683617
CORE0252.V5.COVID.NP.PRO	SRR13219281	PRJNA683617
CORE0252.V5.COVID.OP.PRO	SRR13219279	PRJNA683617
CORE0252.V6.COVID.NP.PRO	SRR13219278	PRJNA683617
CORE0252.V6.COVID.OP.PRO	SRR13219277	PRJNA683617
CORE0253.V1.COVID.NP.PRO	SRR13219276	PRJNA683617
CORE0253.V1.COVID.OP.PRO	SRR13219275	PRJNA683617
CORE0254.V1.COVID.NP.PRO	SRR13219274	PRJNA683617
CORE0254.V1.COVID.OP.PRO	SRR13219273	PRJNA683617
CORE0254.V2.COVID.NP.PRO	SRR13219272	PRJNA683617
CORE0254.V2.COVID.OP.PRO	SRR13219271	PRJNA683617
CORE0255.V1.COVID.NP.PRO	SRR13219270	PRJNA683617
CORE0255.V1.COVID.OP.PRO	SRR13219268	PRJNA683617
CORE0255.V2.COVID.NP.PRO	SRR13219267	PRJNA683617
CORE0255.V2.COVID.OP.PRO	SRR13219266	PRJNA683617
CORE0255.V3.COVID.ETA.PRO	SRR13219265	PRJNA683617
CORE0255.V3.COVID.NP.PRO	SRR13219264	PRJNA683617
CORE0255.V3.COVID.OP.PRO	SRR13219263	PRJNA683617
CORE0255.V4.COVID.NP.PRO	SRR13219262	PRJNA683617
CORE0255.V4.COVID.OP.PRO	SRR13219261	PRJNA683617
CORE0255.V5.COVID.ETA.PRO	SRR13219260	PRJNA683617
CORE0255.V5.COVID.NP.PRO	SRR13219259	PRJNA683617
CORE0255.V5.COVID.OP.PRO	SRR13219256	PRJNA683617
CORE0255.V6.COVID.ETA.PRO	SRR13219255	PRJNA683617
CORE0255.V6.COVID.NP.PRO	SRR13219254	PRJNA683617
CORE0255.V6.COVID.OP.PRO	SRR13219197	PRJNA683617
CORE0255.V7.COVID.NP.PRO	SRR13219196	PRJNA683617
CORE0255.V7.COVID.OP.PRO	SRR13219195	PRJNA683617
CORE0256.V1.COVID.ETA.PRO	SRR13219194	PRJNA683617
CORE0256.V1.COVID.NP.PRO	SRR13219193	PRJNA683617
CORE0256.V1.COVID.OP.PRO	SRR13219192	PRJNA683617
CORE0256.V2.COVID.ETA.PRO	SRR13219191	PRJNA683617
CORE0256.V2.COVID.NP.PRO	SRR13219189	PRJNA683617
CORE0256.V2.COVID.OP.PRO	SRR13219188	PRJNA683617
CORE0257.V1.COVID.NP.PRO	SRR13219187	PRJNA683617
CORE0257.V1.COVID.OP.PRO	SRR13219186	PRJNA683617
CORE0257.V2.COVID.NP.PRO	SRR13219185	PRJNA683617
CORE0257.V2.COVID.OP.PRO	SRR13219184	PRJNA683617
CORE0257.V3.COVID.NP.PRO	SRR13219183	PRJNA683617
CORE0257.V3.COVID.OP.PRO	SRR13219182	PRJNA683617
CORE0258.V1.COVID.NP.PRO	SRR13219181	PRJNA683617
CORE0258.V1.COVID.OP.PRO	SRR13219180	PRJNA683617
CORE0259.V2.COVID.NP.PRO	SRR13219178	PRJNA683617
CORE0259.V2.COVID.OP.PRO	SRR13219177	PRJNA683617
CORE0260.V1.COVID.NP.PRO	SRR13219176	PRJNA683617
CORE0260.V1.COVID.OP.PRO	SRR13219175	PRJNA683617
CORE0260.V2.COVID.NP.PRO	SRR13219174	PRJNA683617
CORE0260.V2.COVID.OP.PRO	SRR13219173	PRJNA683617
CORE0260.V3.COVID.NP.PRO	SRR13219172	PRJNA683617
CORE0260.V3.COVID.OP.PRO	SRR13219171	PRJNA683617
CORE0260.V4.COVID.NP.PRO	SRR13219170	PRJNA683617
CORE0260.V4.COVID.OP.PRO	SRR13219811	PRJNA683617
CORE0261.V1.COVID.ETA.PRO	SRR13219809	PRJNA683617
CORE0261.V3.COVID.ETA.PRO	SRR13219808	PRJNA683617
CORE0261.V4.COVID.ETA.PRO	SRR13219807	PRJNA683617
CORE0262.V1.COVID.NP.PRO	SRR13219806	PRJNA683617
CORE0262.V2.COVID.NP.PRO	SRR13219805	PRJNA683617
CORE0262.V2.COVID.OP.PRO	SRR13219804	PRJNA683617
CORE0263.V1.COVID.ETA.PRO	SRR13219803	PRJNA683617
CORE0263.V1.COVID.NP.PRO	SRR13219802	PRJNA683617
CORE0263.V1.COVID.OP.PRO	SRR13219801	PRJNA683617
CORE0263.V2.COVID.NP.PRO	SRR13219800	PRJNA683617
CORE0263.V2.COVID.OP.PRO	SRR13219798	PRJNA683617
CORE0263.V3.COVID.NP.PRO	SRR13219797	PRJNA683617
CORE0263.V3.COVID.OP.PRO	SRR13219796	PRJNA683617
CORE0263.V4.COVID.NP.PRO	SRR13219795	PRJNA683617
CORE0263.V4.COVID.OP.PRO	SRR13219794	PRJNA683617
CORE0263.V5.COVID.ETA.PRO	SRR13219793	PRJNA683617
CORE0263.V5.COVID.NP.PRO	SRR13219792	PRJNA683617
CORE0263.V5.COVID.OP.PRO	SRR13219791	PRJNA683617
CORE0263.V6.COVID.ETA.PRO	SRR13219790	PRJNA683617
CORE0263.V6.COVID.NP.PRO	SRR13219789	PRJNA683617
CORE0263.V6.COVID.OP.PRO	SRR13219787	PRJNA683617
CORE0264.V2.COVID.ETA.PRO	SRR13219786	PRJNA683617
CORE0264.V2.COVID.NP.PRO	SRR13219785	PRJNA683617
CORE0264.V2.COVID.OP.PRO	SRR13219784	PRJNA683617
CORE0266.V1.COVID.NP.PRO	SRR13219690	PRJNA683617
CORE0266.V1.COVID.OP.PRO	SRR13219689	PRJNA683617
CORE0266.V2.COVID.NP.PRO	SRR13219688	PRJNA683617
CORE0266.V2.COVID.OP.PRO	SRR13219687	PRJNA683617
CORE0269.V1.COVID.NP.PRO	SRR13219683	PRJNA683617
CORE0269.V2.COVID.ETA.PRO	SRR13219682	PRJNA683617
CORE0269.V2.COVID.NP.PRO	SRR13219681	PRJNA683617
CORE0269.V2.COVID.OP.PRO	SRR13219680	PRJNA683617
CORE0269.V3.COVID.ETA.PRO	SRR13219679	PRJNA683617
CORE0269.V3.COVID.NP.PRO	SRR13219678	PRJNA683617
CORE0269.V3.COVID.OP.PRO	SRR13219677	PRJNA683617
CORE0269.V4.COVID.ETA.PRO	SRR13219676	PRJNA683617
CORE0269.V4.COVID.NP.PRO	SRR13219675	PRJNA683617
CORE0269.V4.COVID.OP.PRO	SRR13219674	PRJNA683617
CORE0269.V5.COVID.NP.PRO	SRR13219672	PRJNA683617
CORE0269.V5.COVID.OP.PRO	SRR13219671	PRJNA683617
CORE0270.V1.COVID.NP.PRO	SRR13219670	PRJNA683617
CORE0270.V1.COVID.OP.PRO	SRR13219669	PRJNA683617
CORE0271.V1.COVID.NP.PRO	SRR13219668	PRJNA683617
CORE0271.V1.COVID.OP.PRO	SRR13219667	PRJNA683617
CORE0272.V1.COVID.NP.PRO	SRR13219666	PRJNA683617
CORE0272.V1.COVID.OP.PRO	SRR13219665	PRJNA683617
CORE0272.V2.COVID.ETA.PRO	SRR13219664	PRJNA683617
CORE0272.V2.COVID.NP.PRO	SRR13219663	PRJNA683617
CORE0272.V2.COVID.OP.PRO	SRR13219605	PRJNA683617
CORE0272.V3.COVID.NP.PRO	SRR13219604	PRJNA683617
CORE0272.V3.COVID.OP.PRO	SRR13219603	PRJNA683617
CORE0272.V4.COVID.NP.PRO	SRR13219602	PRJNA683617
CORE0272.V4.COVID.OP.PRO	SRR13219601	PRJNA683617
CORE0272.V5.COVID.ETA.PRO	SRR13219600	PRJNA683617

CORE0272.V5.COVID.NP.PRO	SRR13219599	PRJNA683617
CORE0272.V5.COVID.OP.PRO	SRR13219598	PRJNA683617
CORE0272.V6.COVID.NP.PRO	SRR13219597	PRJNA683617
CORE0272.V6.COVID.OP.PRO	SRR13219596	PRJNA683617
CORE0275.V1.COVID.NP.PRO	SRR13219594	PRJNA683617
CORE0275.V1.COVID.OP.PRO	SRR13219593	PRJNA683617
CORE0275.V2.COVID.NP.PRO	SRR13219592	PRJNA683617
CORE0275.V2.COVID.OP.PRO	SRR13219591	PRJNA683617
CORE0275.V3.COVID.NP.PRO	SRR13219590	PRJNA683617
CORE0275.V3.COVID.OP.PRO	SRR13219589	PRJNA683617
CORE0275.V4.COVID.NP.PRO	SRR13219588	PRJNA683617
CORE0275.V4.COVID.OP.PRO	SRR13219587	PRJNA683617
CORE0276.V1.COVID.NP.PRO	SRR13219586	PRJNA683617
CORE0276.V1.COVID.OP.PRO	SRR13219585	PRJNA683617
CORE0277.V1.COVID.NP.PRO	SRR13219582	PRJNA683617
CORE0277.V1.COVID.OP.PRO	SRR13219581	PRJNA683617
CORE0277.V2.COVID.NP.PRO	SRR13219580	PRJNA683617
CORE0277.V2.COVID.OP.PRO	SRR13219579	PRJNA683617
CORE0279.V1.COVID.NP.PRO	SRR13219521	PRJNA683617
CORE0279.V1.COVID.OP.PRO	SRR13219520	PRJNA683617
CORE0280.V1.COVID.NP.PRO	SRR13219519	PRJNA683617
CORE0280.V1.COVID.OP.PRO	SRR13219518	PRJNA683617
CORE0282.V1.COVID.ETA.PRO	SRR13219517	PRJNA683617
CORE0282.V1.COVID.NP.PRO	SRR13219515	PRJNA683617
CORE0282.V1.COVID.OP.PRO	SRR13219514	PRJNA683617
CORE0282.V2.COVID.NP.PRO	SRR13219513	PRJNA683617
CORE0282.V2.COVID.OP.PRO	SRR13219512	PRJNA683617
CORE0282.V3.COVID.NP.PRO	SRR13219511	PRJNA683617
CORE0282.V3.COVID.OP.PRO	SRR13219510	PRJNA683617
CORE0282.V4.COVID.NP.PRO	SRR13219509	PRJNA683617
CORE0282.V4.COVID.OP.PRO	SRR13219508	PRJNA683617
CORE0282.V5.COVID.NP.PRO	SRR13219507	PRJNA683617
CORE0282.V5.COVID.OP.PRO	SRR13219506	PRJNA683617
CORE0284.V1.COVID.NP.PRO	SRR13219496	PRJNA683617
CORE0284.V1.COVID.OP.PRO	SRR13219495	PRJNA683617
CORE0285.V1.COVID.NP.PRO	SRR13219437	PRJNA683617
CORE0285.V1.COVID.OP.PRO	SRR13219436	PRJNA683617
CORE0285.V2.COVID.NP.PRO	SRR13219435	PRJNA683617
CORE0285.V2.COVID.OP.PRO	SRR13219434	PRJNA683617
CORE0285.V3.COVID.NP.PRO	SRR13219433	PRJNA683617
CORE0285.V3.COVID.OP.PRO	SRR13219432	PRJNA683617
CORE0285.V4.COVID.NP.PRO	SRR13219431	PRJNA683617
CORE0285.V4.COVID.OP.PRO	SRR13219430	PRJNA683617
CORE0286.V1.COVID.NP.PRO	SRR13219429	PRJNA683617
CORE0286.V1.COVID.OP.PRO	SRR13219428	PRJNA683617
CORE0286.V2.COVID.NP.PRO	SRR13219426	PRJNA683617
CORE0286.V2.COVID.OP.PRO	SRR13219425	PRJNA683617
CORE0288.V1.COVID.NP.PRO	SRR13219421	PRJNA683617
CORE0288.V1.COVID.OP.PRO	SRR13219420	PRJNA683617
CORE0288.V2.COVID.NP.PRO	SRR13219419	PRJNA683617
CORE0288.V2.COVID.OP.PRO	SRR13219418	PRJNA683617
CORE0288.V3.COVID.ETA.PRO	SRR13219416	PRJNA683617
CORE0288.V3.COVID.NP.PRO	SRR13219415	PRJNA683617
CORE0288.V3.COVID.OP.PRO	SRR13219414	PRJNA683617
CORE0288.V4.COVID.ETA.PRO	SRR13219413	PRJNA683617
CORE0288.V4.COVID.NP.PRO	SRR13219412	PRJNA683617
CORE0288.V4.COVID.OP.PRO	SRR13219355	PRJNA683617
CORE0288.V5.COVID.ETA.PRO	SRR13219354	PRJNA683617
CORE0288.V5.COVID.NP.PRO	SRR13219353	PRJNA683617
CORE0288.V5.COVID.OP.PRO	SRR13219352	PRJNA683617
CORE0288.V6.COVID.NP.PRO	SRR13219351	PRJNA683617
CORE0288.V6.COVID.OP.PRO	SRR13219349	PRJNA683617
CORE0288.V7.COVID.NP.PRO	SRR13219348	PRJNA683617
CORE0288.V7.COVID.OP.PRO	SRR13219347	PRJNA683617
CORE0288.V8.COVID.NP.PRO	SRR13219346	PRJNA683617
CORE0288.V8.COVID.OP.PRO	SRR13219345	PRJNA683617
CORE0288.V9.COVID.NP.PRO	SRR13219344	PRJNA683617
CORE0288.V9.COVID.OP.PRO	SRR13219343	PRJNA683617
CORE0289.V1.COVID.NP.PRO	SRR13219342	PRJNA683617
CORE0289.V1.COVID.OP.PRO	SRR13219341	PRJNA683617
CORE0290.V1.COVID.NP.PRO	SRR13219340	PRJNA683617
CORE0290.V1.COVID.OP.PRO	SRR13219338	PRJNA683617
CORE0290.V2.COVID.NP.PRO	SRR13219337	PRJNA683617
CORE0290.V2.COVID.OP.PRO	SRR13219336	PRJNA683617
CORE0291.V1.COVID.NP.PRO	SRR13219335	PRJNA683617
CORE0291.V1.COVID.OP.PRO	SRR13219334	PRJNA683617
CORE0292.V1.COVID.NP.PRO	SRR13219333	PRJNA683617
CORE0292.V1.COVID.OP.PRO	SRR13219332	PRJNA683617
CORE0293.V1.COVID.NP.PRO	SRR13219331	PRJNA683617
CORE0293.V1.COVID.OP.PRO	SRR13219330	PRJNA683617
CORE0294.V1.nonCOVID.NP.PRO	SRR13219329	PRJNA683617
CORE0294.V1.nonCOVID.NP.PS	SRR13219253	PRJNA683617
CORE0294.V1.nonCOVID.OP.PRO	SRR13219252	PRJNA683617
CORE0294.V1.nonCOVID.OP.PS	SRR13219251	PRJNA683617
CORE0295.V1.nonCOVID.NP.PRO	SRR13219250	PRJNA683617
CORE0295.V1.nonCOVID.NP.PS	SRR13219249	PRJNA683617
CORE0295.V1.nonCOVID.OP.PRO	SRR13219248	PRJNA683617
CORE0295.V1.nonCOVID.OP.PS	SRR13219247	PRJNA683617
CORE0296.V1.nonCOVID.NP.PRO	SRR13219246	PRJNA683617
CORE0296.V1.nonCOVID.NP.PS	SRR13219245	PRJNA683617
CORE0296.V1.nonCOVID.OP.PRO	SRR13219244	PRJNA683617
CORE0296.V1.nonCOVID.OP.PS	SRR13219242	PRJNA683617
CORE0297.V1.COVID.NP.PRO	SRR13219241	PRJNA683617
CORE0297.V1.COVID.OP.PRO	SRR13219240	PRJNA683617
CORE0298.V1.COVID.NP.PRO	SRR13219239	PRJNA683617
CORE0298.V1.COVID.OP.PRO	SRR13219238	PRJNA683617
CORE0298.V2.COVID.NP.PRO	SRR13219237	PRJNA683617
CORE0298.V2.COVID.OP.PRO	SRR13219236	PRJNA683617
DNAfreewater1	SRR13219165	PRJNA683617
DNAfreewater10	SRR13219163	PRJNA683617
DNAfreewater11	SRR13219162	PRJNA683617
DNAfreewater12	SRR13219161	PRJNA683617
DNAfreewater13	SRR13219160	PRJNA683617
DNAfreewater14	SRR13219159	PRJNA683617
DNAfreewater15	SRR13219158	PRJNA683617
DNAfreewater16	SRR13219157	PRJNA683617
DNAfreewater17	SRR13219156	PRJNA683617
DNAfreewater18	SRR13219155	PRJNA683617
DNAfreewater19	SRR13219154	PRJNA683617
DNAfreewater2	SRR13219152	PRJNA683617
DNAfreewater20	SRR13219151	PRJNA683617
DNAfreewater21	SRR13219150	PRJNA683617
DNAfreewater22	SRR13219149	PRJNA683617
DNAfreewater3	SRR13219148	PRJNA683617
DNAfreewater4	SRR13219147	PRJNA683617
DNAfreewater5	SRR13219146	PRJNA683617
DNAfreewater6	SRR13219145	PRJNA683617
DNAfreewater7	SRR13219144	PRJNA683617
DNAfreewater8	SRR13219143	PRJNA683617
DNAfreewater9	SRR13219141	PRJNA683617
Empty1	SRR13219140	PRJNA683617
Empty10	SRR13219139	PRJNA683617
Empty11	SRR13219137	PRJNA683617
Empty12	SRR13219783	PRJNA683617
Empty13	SRR13219782	PRJNA683617
Empty14	SRR13219781	PRJNA683617
Empty15	SRR13219780	PRJNA683617
Empty16	SRR13219779	PRJNA683617
Empty2	SRR13219778	PRJNA683617
Empty3	SRR13219776	PRJNA683617
Empty4	SRR13219775	PRJNA683617
Empty5	SRR13219774	PRJNA683617
Empty6	SRR13219773	PRJNA683617
Empty7	SRR13219772	PRJNA683617
Empty8	SRR13219771	PRJNA683617
Empty9	SRR13219770	PRJNA683617
Extractemptywell1	SRR13219769	PRJNA683617
Extractemptywell10	SRR13219768	PRJNA683617
Extractemptywell11	SRR13219767	PRJNA683617
Extractemptywell2	SRR13219765	PRJNA683617
Extractemptywell3	SRR13219764	PRJNA683617
Extractemptywell4	SRR13219763	PRJNA683617
Extractemptywell5	SRR13219762	PRJNA683617
Extractemptywell6	SRR13219761	PRJNA683617
Extractemptywell7	SRR13219760	PRJNA683617
Extractemptywell8	SRR13219759	PRJNA683617
Extractemptywell9	SRR13219758	PRJNA683617
ExtrBlk.0702.Control.Blank.PRO	SRR13219757	PRJNA683617
ExtrBlk.0706.Control.Blank.PRO	SRR13219756	PRJNA683617
ExtrBlk1.0709.Control.Blank.PRO	SRR13219754	PRJNA683617
ExtrBlk2.0709.Control.Blank.PRO	SRR13219753	PRJNA683617
HEALTHY.V1.Control.NP.PRO	SRR13219752	PRJNA683617
HEALTHY.V1.Control.NP.PS	SRR13219751	PRJNA683617
HEALTHY.V1.Control.OP.PRO	SRR13219750	PRJNA683617
HEALTHY.V1.Control.OP.PS	SRR13219749	PRJNA683617
mockdna1	SRR13219748	PRJNA683617
mockdna10	SRR13219747	PRJNA683617
mockdna11	SRR13219746	PRJNA683617
mockdna12	SRR13219745	PRJNA683617
mockdna13	SRR13219743	PRJNA683617
mockdna14	SRR13219742	PRJNA683617
mockdna15	SRR13219741	PRJNA683617

mockdna16	SRR13219740	PRJNA683617
mockdna17	SRR13219739	PRJNA683617
mockdna18	SRR13219738	PRJNA683617
mockdna19	SRR13219737	PRJNA683617
mockdna2	SRR13219736	PRJNA683617
mockdna20	SRR13219735	PRJNA683617
mockdna21	SRR13219734	PRJNA683617
mockdna22	SRR13219732	PRJNA683617
mockdna3	SRR13219731	PRJNA683617
mockdna4	SRR13219730	PRJNA683617
mockdna5	SRR13219729	PRJNA683617
mockdna6	SRR13219728	PRJNA683617
mockdna7	SRR13219727	PRJNA683617
mockdna8	SRR13219726	PRJNA683617
mockdna9	SRR13219725	PRJNA683617
NPswab.0702.Control.Swab.PRO	SRR13219724	PRJNA683617
NPswab.0706.Control.Swab.PRO	SRR13219723	PRJNA683617
NPswab.0707.Control.Swab.PRO	SRR13219578	PRJNA683617
NPswab.0708.Control.Swab.PRO	SRR13219577	PRJNA683617
NPswab.0709.Control.Swab.PRO	SRR13219576	PRJNA683617
NPswab1.0708.Control.Swab.PS	SRR13219575	PRJNA683617
NPswab2.0708.Control.Swab.PS	SRR13219574	PRJNA683617
OPswab.0630.Control.Swab.PRO	SRR13219573	PRJNA683617
OPswab.0701.Control.Swab.PRO	SRR13219572	PRJNA683617
OPswab.0709.Control.Swab.PRO	SRR13219571	PRJNA683617
Saline.0622.Control.Saline.DTT.PRO	SRR13219570	PRJNA683617
Saline.0622.Control.Saline.DTT.PS	SRR13219569	PRJNA683617
Saline.0708.Control.Saline.PRO	SRR13219566	PRJNA683617
Saline1.0709.Control.Saline.PRO	SRR13219565	PRJNA683617
Saline2.0709.Control.Saline.PRO	SRR13219564	PRJNA683617
ESC.1.1.NPL	SRR13052326	PRJNA678105
ESC.1.1.NPR	SRR13052325	PRJNA678105
ESC.1.1.OPL	SRR13052278	PRJNA678105
ESC.1.1.OPR	SRR13052302	PRJNA678105
ESC.1.10.NPR	SRR13052291	PRJNA678105
ESC.1.10.OPL	SRR13052216	PRJNA678105
ESC.1.10.OPR	SRR13052205	PRJNA678105
ESC.1.11.NPL	SRR13052130	PRJNA678105
ESC.1.11.NPR	SRR13052119	PRJNA678105
ESC.1.11.OPL	SRR13052108	PRJNA678105
ESC.1.11.OPR	SRR13052324	PRJNA678105
ESC.1.12.NPL	SRR13052249	PRJNA678105
ESC.1.12.NPR	SRR13052238	PRJNA678105
ESC.1.12.OPL	SRR13052227	PRJNA678105
ESC.1.12.OPR	SRR13052184	PRJNA678105
ESC.1.13.NPL	SRR13052173	PRJNA678105
ESC.1.13.NPR	SRR13052066	PRJNA678105
ESC.1.13.OPL	SRR13052055	PRJNA678105
ESC.1.13.OPR	SRR13052044	PRJNA678105
ESC.1.14.NPL	SRR13052289	PRJNA678105
ESC.1.14.NPR	SRR13052277	PRJNA678105
ESC.1.14.OPL	SRR13052266	PRJNA678105
ESC.1.14.OPR	SRR13052159	PRJNA678105
ESC.1.15.NPL	SRR13052148	PRJNA678105
ESC.1.15.NPR	SRR13052137	PRJNA678105
ESC.1.15.OPL	SRR13052094	PRJNA678105
ESC.1.15.OPR	SRR13052083	PRJNA678105
ESC.1.16.NPL	SRR13052072	PRJNA678105
ESC.1.16.NPR	SRR13052317	PRJNA678105
ESC.1.16.OPL	SRR13052306	PRJNA678105
ESC.1.16.OPR	SRR13052301	PRJNA678105
ESC.1.17.NPL	SRR13052300	PRJNA678105
ESC.1.17.NPR	SRR13052299	PRJNA678105
ESC.1.17.OPL	SRR13052298	PRJNA678105
ESC.1.17.OPR	SRR13052297	PRJNA678105
ESC.1.18.NPL	SRR13052296	PRJNA678105
ESC.1.18.NPR	SRR13052295	PRJNA678105
ESC.1.18.OPL	SRR13052294	PRJNA678105
ESC.1.18.OPR	SRR13052293	PRJNA678105
ESC.1.20.NPL	SRR13052223	PRJNA678105
ESC.1.20.NPR	SRR13052222	PRJNA678105
ESC.1.20.OPL	SRR13052221	PRJNA678105
ESC.1.20.OPR	SRR13052220	PRJNA678105
ESC.1.20a.NPL	SRR13052219	PRJNA678105
ESC.1.20a.NPR	SRR13052218	PRJNA678105
ESC.1.20a.OPL	SRR13052217	PRJNA678105
ESC.1.20a.OPR	SRR13052215	PRJNA678105
ESC.1.21.NPL	SRR13052214	PRJNA678105
ESC.1.21.NPR	SRR13052213	PRJNA678105
ESC.1.21.OPL	SRR13052212	PRJNA678105
ESC.1.21.OPR	SRR13052211	PRJNA678105
ESC.1.23a.NPL	SRR13052201	PRJNA678105
ESC.1.23a.NPR	SRR13052200	PRJNA678105
ESC.1.23a.OPL	SRR13052199	PRJNA678105
ESC.1.23a.OPR	SRR13052198	PRJNA678105
ESC.1.24.NPR	SRR13052197	PRJNA678105
ESC.1.24.OPL	SRR13052196	PRJNA678105
ESC.1.24.OPR	SRR13052195	PRJNA678105
ESC.1.25.NPL	SRR13052129	PRJNA678105
ESC.1.25.NPR	SRR13052128	PRJNA678105
ESC.1.25.OPL	SRR13052127	PRJNA678105
ESC.1.25.OPR	SRR13052126	PRJNA678105
ESC.1.26.NPL	SRR13052125	PRJNA678105
ESC.1.26.NPR	SRR13052124	PRJNA678105
ESC.1.26.OPL	SRR13052123	PRJNA678105
ESC.1.26.OPR	SRR13052122	PRJNA678105
ESC.1.27.NPL	SRR13052121	PRJNA678105
ESC.1.27.NPR	SRR13052120	PRJNA678105
ESC.1.27.OPL	SRR13052118	PRJNA678105
ESC.1.27.OPR	SRR13052117	PRJNA678105
ESC.1.28.NPL	SRR13052116	PRJNA678105
ESC.1.28.NPR	SRR13052115	PRJNA678105
ESC.1.28.OPL	SRR13052114	PRJNA678105
ESC.1.28.OPR	SRR13052113	PRJNA678105
ESC.1.29.NPL	SRR13052112	PRJNA678105
ESC.1.29.NPR	SRR13052111	PRJNA678105
ESC.1.29.OPL	SRR13052110	PRJNA678105
ESC.1.29.OPR	SRR13052109	PRJNA678105
ESC.1.3.NPL	SRR13052107	PRJNA678105
ESC.1.3.NPR	SRR13052106	PRJNA678105
ESC.1.3.OPL	SRR13052105	PRJNA678105
ESC.1.3.OPR	SRR13052104	PRJNA678105
ESC.1.30.NPL	SRR13052103	PRJNA678105
ESC.1.30.NPR	SRR13052102	PRJNA678105
ESC.1.30.OPL	SRR13052101	PRJNA678105
ESC.1.30.OPR	SRR13052100	PRJNA678105
ESC.1.31.NPL	SRR13052099	PRJNA678105
ESC.1.31.NPR	SRR13052034	PRJNA678105
ESC.1.31.OPL	SRR13052323	PRJNA678105
ESC.1.31.OPR	SRR13052258	PRJNA678105
ESC.1.32.NPL	SRR13052257	PRJNA678105
ESC.1.32.NPR	SRR13052256	PRJNA678105
ESC.1.32.OPL	SRR13052255	PRJNA678105
ESC.1.32.OPR	SRR13052254	PRJNA678105
ESC.1.33.NPL	SRR13052253	PRJNA678105
ESC.1.33.NPR	SRR13052252	PRJNA678105
ESC.1.33.OPL	SRR13052251	PRJNA678105
ESC.1.33.OPR	SRR13052250	PRJNA678105
ESC.1.34.NPL	SRR13052248	PRJNA678105
ESC.1.34.NPR	SRR13052247	PRJNA678105
ESC.1.34.OPL	SRR13052246	PRJNA678105
ESC.1.34.OPR	SRR13052245	PRJNA678105
ESC.1.35.NPL	SRR13052244	PRJNA678105
ESC.1.35.NPR	SRR13052243	PRJNA678105
ESC.1.35.OPL	SRR13052242	PRJNA678105
ESC.1.35.OPR	SRR13052241	PRJNA678105
ESC.1.36.NPL	SRR13052240	PRJNA678105
ESC.1.36.NPR	SRR13052239	PRJNA678105
ESC.1.36.OPL	SRR13052237	PRJNA678105
ESC.1.36.OPR	SRR13052236	PRJNA678105
ESC.1.37.NPL	SRR13052235	PRJNA678105
ESC.1.37.NPR	SRR13052234	PRJNA678105
ESC.1.37.OPL	SRR13052233	PRJNA678105
ESC.1.37.OPR	SRR13052232	PRJNA678105
ESC.1.38.NPL	SRR13052231	PRJNA678105
ESC.1.38.NPR	SRR13052230	PRJNA678105
ESC.1.38.OPL	SRR13052229	PRJNA678105
ESC.1.38.OPR	SRR13052228	PRJNA678105
ESC.1.39.NPL	SRR13052194	PRJNA678105
ESC.1.39.NPR	SRR13052193	PRJNA678105
ESC.1.39.OPL	SRR13052192	PRJNA678105
ESC.1.39.OPR	SRR13052191	PRJNA678105
ESC.1.4.NPL	SRR13052190	PRJNA678105
ESC.1.4.NPR	SRR13052189	PRJNA678105
ESC.1.4.OPL	SRR13052188	PRJNA678105
ESC.1.4.OPR	SRR13052187	PRJNA678105
ESC.1.40.NPL	SRR13052186	PRJNA678105
ESC.1.40.NPR	SRR13052185	PRJNA678105
ESC.1.40.OPL	SRR13052183	PRJNA678105
ESC.1.40.OPR	SRR13052182	PRJNA678105
ESC.1.41.NPL	SRR13052181	PRJNA678105
ESC.1.41.NPR	SRR13052180	PRJNA678105

ESC.1.41.OPL	SRR13052179	PRJNA678105
ESC.1.41.OPR	SRR13052178	PRJNA678105
ESC.1.42.NPL	SRR13052177	PRJNA678105
ESC.1.42.NPR	SRR13052176	PRJNA678105
ESC.1.42.OPL	SRR13052175	PRJNA678105
ESC.1.42.OPR	SRR13052174	PRJNA678105
ESC.1.43.NPL	SRR13052172	PRJNA678105
ESC.1.43.NPR	SRR13052171	PRJNA678105
ESC.1.43.OPL	SRR13052170	PRJNA678105
ESC.1.43.OPR	SRR13052169	PRJNA678105
ESC.1.44.NPL	SRR13052168	PRJNA678105
ESC.1.44.NPR	SRR13052167	PRJNA678105
ESC.1.44.OPL	SRR13052166	PRJNA678105
ESC.1.44.OPR	SRR13052165	PRJNA678105
ESC.1.45.NPL	SRR13052164	PRJNA678105
ESC.1.45.NPR	SRR13052163	PRJNA678105
ESC.1.45.OPL	SRR13052065	PRJNA678105
ESC.1.45.OPR	SRR13052064	PRJNA678105
ESC.1.46.NPL	SRR13052063	PRJNA678105
ESC.1.46.NPR	SRR13052062	PRJNA678105
ESC.1.46.OPL	SRR13052061	PRJNA678105
ESC.1.46.OPR	SRR13052060	PRJNA678105
ESC.1.47.NPL	SRR13052059	PRJNA678105
ESC.1.47.NPR	SRR13052058	PRJNA678105
ESC.1.47.OPL	SRR13052057	PRJNA678105
ESC.1.47.OPR	SRR13052056	PRJNA678105
ESC.1.49.NPL	SRR13052050	PRJNA678105
ESC.1.49.NPR	SRR13052049	PRJNA678105
ESC.1.49.OPL	SRR13052048	PRJNA678105
ESC.1.49.OPR	SRR13052047	PRJNA678105
ESC.1.5.NPL	SRR13052046	PRJNA678105
ESC.1.5.NPR	SRR13052045	PRJNA678105
ESC.1.5.OPL	SRR13052043	PRJNA678105
ESC.1.5.OPR	SRR13052042	PRJNA678105
ESC.1.50.NPL	SRR13052041	PRJNA678105
ESC.1.50.NPR	SRR13052040	PRJNA678105
ESC.1.50.OPL	SRR13052039	PRJNA678105
ESC.1.50.OPR	SRR13052038	PRJNA678105
ESC.1.51.NPL	SRR13052037	PRJNA678105
ESC.1.51.NPR	SRR13052036	PRJNA678105
ESC.1.51.OPL	SRR13052035	PRJNA678105
ESC.1.51.OPR	SRR13052290	PRJNA678105
ESC.1.52.NPL	SRR13052288	PRJNA678105
ESC.1.52.NPR	SRR13052287	PRJNA678105
ESC.1.52.OPL	SRR13052286	PRJNA678105
ESC.1.52.OPR	SRR13052285	PRJNA678105
ESC.1.53.NPL	SRR13052284	PRJNA678105
ESC.1.53.NPR	SRR13052283	PRJNA678105
ESC.1.53.OPL	SRR13052282	PRJNA678105
ESC.1.53.OPR	SRR13052281	PRJNA678105
ESC.1.54.NPL	SRR13052280	PRJNA678105
ESC.1.54.NPR	SRR13052279	PRJNA678105
ESC.1.54.OPL	SRR13052276	PRJNA678105
ESC.1.55.NPL	SRR13052275	PRJNA678105
ESC.1.55.NPR	SRR13052274	PRJNA678105
ESC.1.55.OPL	SRR13052273	PRJNA678105
ESC.1.55.OPR	SRR13052272	PRJNA678105
ESC.1.55a.NPL	SRR13052271	PRJNA678105
ESC.1.55a.NPR	SRR13052270	PRJNA678105
ESC.1.55a.OPL	SRR13052269	PRJNA678105
ESC.1.55a.OPR	SRR13052268	PRJNA678105
ESC.1.56.NPL	SRR13052267	PRJNA678105
ESC.1.56.NPR	SRR13052265	PRJNA678105
ESC.1.56.OPL	SRR13052264	PRJNA678105
ESC.1.56.OPR	SRR13052263	PRJNA678105
ESC.1.57.NPL	SRR13052262	PRJNA678105
ESC.1.57.NPR	SRR13052261	PRJNA678105
ESC.1.57.OPL	SRR13052260	PRJNA678105
ESC.1.57.OPR	SRR13052259	PRJNA678105
ESC.1.58.NPL	SRR13052162	PRJNA678105
ESC.1.58.NPR	SRR13052161	PRJNA678105
ESC.1.58.OPL	SRR13052160	PRJNA678105
ESC.1.58.OPR	SRR13052158	PRJNA678105
ESC.1.59.NPL	SRR13052157	PRJNA678105
ESC.1.59.NPR	SRR13052156	PRJNA678105
ESC.1.59.OPL	SRR13052155	PRJNA678105
ESC.1.59.OPR	SRR13052154	PRJNA678105
ESC.1.6.NPL	SRR13052153	PRJNA678105
ESC.1.6.NPR	SRR13052152	PRJNA678105
ESC.1.6.OPL	SRR13052151	PRJNA678105
ESC.1.6.OPR	SRR13052150	PRJNA678105
ESC.1.60.NPL	SRR13052149	PRJNA678105
ESC.1.60.NPR	SRR13052147	PRJNA678105
ESC.1.60.OPL	SRR13052146	PRJNA678105
ESC.1.60.OPR	SRR13052145	PRJNA678105
ESC.1.61.NPL	SRR13052144	PRJNA678105
ESC.1.61.NPR	SRR13052143	PRJNA678105
ESC.1.61.OPL	SRR13052142	PRJNA678105
ESC.1.61.OPR	SRR13052141	PRJNA678105
ESC.1.62.NPL	SRR13052140	PRJNA678105
ESC.1.62.NPR	SRR13052139	PRJNA678105
ESC.1.62.OPL	SRR13052138	PRJNA678105
ESC.1.62.OPR	SRR13052136	PRJNA678105
ESC.1.63.NPL	SRR13052135	PRJNA678105
ESC.1.63.NPR	SRR13052134	PRJNA678105
ESC.1.63.OPL	SRR13052133	PRJNA678105
ESC.1.63.OPR	SRR13052132	PRJNA678105
ESC.1.64.NPL	SRR13052131	PRJNA678105
ESC.1.64.NPR	SRR13052098	PRJNA678105
ESC.1.64.OPL	SRR13052097	PRJNA678105
ESC.1.64.OPR	SRR13052096	PRJNA678105
ESC.1.65.NPL	SRR13052095	PRJNA678105
ESC.1.65.NPR	SRR13052093	PRJNA678105
ESC.1.65.OPL	SRR13052092	PRJNA678105
ESC.1.65.OPR	SRR13052091	PRJNA678105
ESC.1.66.NPL	SRR13052090	PRJNA678105
ESC.1.66.NPR	SRR13052089	PRJNA678105
ESC.1.66.OPL	SRR13052088	PRJNA678105
ESC.1.66.OPR	SRR13052087	PRJNA678105
ESC.1.67.NPL	SRR13052086	PRJNA678105
ESC.1.67.NPR	SRR13052085	PRJNA678105
ESC.1.67.OPL	SRR13052084	PRJNA678105
ESC.1.67.OPR	SRR13052082	PRJNA678105
ESC.1.68.NPL	SRR13052081	PRJNA678105
ESC.1.68.NPR	SRR13052080	PRJNA678105
ESC.1.68.OPL	SRR13052079	PRJNA678105
ESC.1.68.OPR	SRR13052078	PRJNA678105
ESC.1.69.NPL	SRR13052077	PRJNA678105
ESC.1.69.NPR	SRR13052076	PRJNA678105
ESC.1.69.OPL	SRR13052075	PRJNA678105
ESC.1.69.OPR	SRR13052074	PRJNA678105
ESC.1.70.NPL	SRR13052073	PRJNA678105
ESC.1.70.NPR	SRR13052071	PRJNA678105
ESC.1.70.OPL	SRR13052070	PRJNA678105
ESC.1.70.OPR	SRR13052069	PRJNA678105
ESC.1.71.NPL	SRR13052068	PRJNA678105
ESC.1.71.NPR	SRR13052067	PRJNA678105
ESC.1.71.OPL	SRR13052322	PRJNA678105
ESC.1.71.OPR	SRR13052321	PRJNA678105
ESC.1.72.NPL	SRR13052320	PRJNA678105
ESC.1.72.NPR	SRR13052319	PRJNA678105
ESC.1.72.OPL	SRR13052318	PRJNA678105
ESC.1.72.OPR	SRR13052316	PRJNA678105
