# Supplementary material for: Signatures of COVID-19 Severity and Immune Response in the Respiratory Tract Microbiome
Source: mBio. 2021 Aug 17;12(4):e01777-21. doi: 10.1128/mBio.01777-21 (PMC8406335; doi:10.1128/mBio.01777-21)
Supplement: TEXT S1 [file mbio.01777-21-s0001.pdf]

Supplementary Methods

Signatures of COVID-19 severity and immune response in the respiratory tract microbiome

Carter Merenstein[1]*, Guanxiang Liang[1]*, Samantha A. Whiteside[2]*, Ana G. Cobián-Güemes [1], Madeline S. Merlino[1], Louis J. Taylor[1], Abigail Glascock[1], Kyle Bittinger[3], Ceylan Tanes[3], Jevon Graham-Wooten[2], Layla A. Khatib[2], Ayannah S. Fitzgerald[2], Shantan Reddy[1], Amy E. Baxter[4,5], Josephine R. Giles[4,5], Derek A. Oldridge[5,6], Nuala J. Meyer[2], E. John Wherry[4,5], John E. McGinniss[2], Frederic D. Bushman[1#], and Ronald G. Collman[2#]

Human subjects

Following informed consent obtained under protocol #823392 approved by the University of Pennsylvania IRB, samples were collected at the Hospital of the University of Pennsylvania beginning on March 23, 2020 (two weeks after the first case was reported in Philadelphia), continuing through the first wave of the epidemic, and ending on July 10, 2020. Most patients were hospitalized for COVID-19 but a few were admitted for other reasons and found to have SARS-CoV-2 infection after hospitalization. Subjects were enrolled from both ward and ICU populations and included unselected sequential consenting subjects up to the maximum daily consenting/collection capacity. Collections began a median of 4 days following hospitalization (generally within one week of hospitalization or identification of COVID+ status if post-admission) and continued 2-3 times weekly until discharge, death, or change in status precluding respiratory tract collections (e.g., noninvasive ventilation modalities) or 30 days from

enrollment. Oropharyngeal (OP) and nasopharyngeal (NP) samples were obtained using flocked swabs (Copan Diagnostics) and endotracheal aspirate (ETA) samples were obtained from intubated patients by suction as previously described (1). Swabs and ETA were frozen (-80°C) within 1 hour of collection and stored until extraction. For some collection days (typically during the first 1-2 weeks of enrollment), additional OP and NP swabs were obtained and eluted in viral transport media (VTM) for SARS-CoV-2 analysis as previously described (2). COVID-19 patients were classified clinically based on the maximum score reached during hospitalization using the 11-point ordinal WHO COVID-19 progression scale (3). Non-COVID control subjects, who were all hospitalized in the intensive care unit (ICU) with a variety of underlying disorders, were consecutive consenting patients admitted to the ICU in two periods (September/October 2019 and July 2020), with individuals from the latter time period all confirmed COVID-negative (Table S1).

Total patient and sample numbers subjected to 16S rRNA gene sequencing were: COVID-19 patients: 83; samples: 506 (OP: 226; NP: 221; ETA: 59); Non-COVID patients: 13; samples: 73 (OP: 34; NP: 34; ETA: 5); (Table S2). Sequencing controls included negative control blanks and synthetic positive controls (4).

<u>16S rRNA marker gene sequencing and analysis</u>

DNA extraction, PCR amplification and sequencing were carried out as previously described (5, 6), with some modifications. DNA was extracted from swabs or 200 µL ETA using DNeasy PowerSoil or PowerSoil Pro kits (Qiagen), incorporating a 95°C x 10 minutes incubation to inactivate SARS-CoV-2. Sequencing libraries were prepared using Q5 Polymerase (New England Biolabs) and primers targeting the V1V2 regions of the 16S rRNA gene (27F and 338R; Table S5). The resulting libraries were quantified using the Quant-iT PicoGreen Assay Kit (Invitrogen), pooled in equimolar quantities, and then sequenced with 250-bp paired-end reads

using the 500 cycle Rapid v2 SBS and PE Cluster HiSeq kits (Illumina) on a HiSeq 2500 in rapid run mode.

16S rRNA gene V1V2 region sequencing data were analyzed using the QIIME2 pipeline as described (7). Demultiplexed sequencing reads were imported into QIIME2 pipeline, and DADA2 was used for sequence quality filtering and denoising to generate a feature table (8). Samples with less than 1000 assigned reads were excluded from further analysis. Taxonomy was classified using the naïve Bayes classifier from QIIME2 and the SILVA database (v138.1) (9). To control for environmental contamination, we removed taxa with a strong negative correlation between log transformed relative abundance and total DNA concentration (10). The following taxa with a significant negative correlation (FDR<0.05) were eliminated: "Ralstonia", "Sphingomonas", "Chloroplast", "Massilia", "Methylobacterium.Methylorubrum", "Aenigmarchaeales", "Acinetobacter", "uncultured [family] ncultured[genus]", "Sphingomonadaceae [family] unassigned [genus]", "Comamonadaceae [family] unassigned [genus]."

Alpha diversity and UniFrac distances were calculated using the vegan (v2.5-6) package in R (v4.0.2). The principal coordinate analysis (PCoA) was performed using the ape package (v5.3) in R with UniFrac distances. The vegan package was used for PERMANOVA tests. Because patients with more severe COVID-19 had a larger average number of samples than those with more moderate disease, patients were randomly subsampled to one sample per patient 1000 times when calculating PERMANOVA tests, and mean p values were reported.

To determine the rate at which sample composition diverged within a patient, we compared weighted UniFrac distance to the first sample from each patient in each sample type. To account for different sampling duration, only samples from the first 7 days were included. A linear slope was calculated for each patient, and these slopes were compared using a Kruskal-Wallis non-parametric test, so that patients with a larger number of samples were not more heavily weighted.

16S rRNA gene V1V2 region sequencing data from NP, OP and lungs (bronchoalveolar lavage; BAL) of healthy controls were previously reported (1, 11, 12). These data were acquired using the Roche 454 GS-FLX platform; our group previously showed that differences in sequencing results between the Illumina and Roche systems are minimal for these samples (1). Healthy NP, OP and BAL 16S rRNA gene reads were assigned taxonomy directly, without preprocessing, using the naïve Bayes classifier from QIIME2. When calculating unweighted UniFrac distances between these samples and samples from our current study, a threshold of 1% abundance was used to remove rare taxa from our data, to account for differences in sequencing depth between the two datasets.

<u>qPCR to detect small circular DNA viruses</u>

Total microbial DNA was amplified using Phi29 DNA polymerase (New England BioLabs) and random hexamers with the following program: 35ºC for 5 minutes, 34ºC for 10 minutes, 33ºC for 15 minutes, 32ºC for 20 minutes, 31ºC for 30 minutes, 30ºC for 16 hours and 65 ºC for 15 minutes. Each 20 µL reaction contained 10 units Phi29 DNA polymerase, 0.1 mg/mL BSA, 1X Phi29 buffer, 2 uM random hexamers, 1 mM dNTP, and 1 µL of DNA. QPCR was performed using TaqMan Fast Universal PCR (Thermo Fisher Scientific) on a QuantStudio 3 Real Time PCR System (Applied Biosystems) with the following program: 20 sec at 95°C for 1 cycle, and 40 cycles of 95°C for 3 sec and 60°C for 30 sec. Each reaction contained 900 nM of each primer and 250 nM probe. Primers and probes that target *Anelloviridae* type species Torque Teno Virus (TTV) and *Redondoviridae* (RV) have been described previously (13, 14). Sequences are listed in Supplementary Table S5. qPCR replicates were performed in triplicate and the average genome copy number was used. Mean values are in Table S3.

<u>qPCR to quantify levels of SARS-CoV-2 RNA</u>

Levels of SARS-CoV-2 RNA were quantified as described (2), RNA was extracted from 140 μL of swab eluate, neat ETA or saliva using the Qiagen QIAamp Viral RNA Mini Kit. The RT-qPCR assay used the CDC 2019-nCoV_N1 primer-probe set (2) and sequences are listed in Table S5. RT-qPCR reactions were prepared as follows: 8.5 μl dH_2O, 0.5 μl N1-F (20 μM), 0.5 μl N1-R (20 μM), 0.5 μl N1-P (5 μM), 5.0 μl TaqMan™ Fast Virus 1-Step Master Mix were combined per reaction. 5 μl of extracted RNA was added to 15 μl of prepared master mix for a final volume of 20 μl per reaction. Final concentrations of 2019-nCoV_N1-F and 2019-nCoV_N1-R primers were 500nM and the final concentration of the 2019-nCoV_N1-P probe was 125nM. The assay was carried out using an Applied Biosystems™ QuantStudio™ 5 Real-Time PCR System. The thermocycler conditions were: 5 minutes at 50°C, 20 seconds at 95°C, and 40 cycles of 3 seconds at 95°C and 30 seconds at 60°C.

Clinical and immune data

Results from clinical laboratory tests performed during the patients' hospitalization were extracted from the electronic medical record. For lymphocyte and neutrophil values, which are measured frequently, the average value of the three days surrounding the date of microbiome sampling was used. Cellular immune profiling data was acquired on peripheral blood mononuclear cells using flow cytometry as described (15). Cell subsets queried for associations with microbiome variables are listed in Table S4D. The unbiased Uniform Manifold Approximation and Projection (UMAP) approach was used to distill 193 individual immune components into two principal components (15). The microbiome unweighted UniFrac PCoA was compared with blood cellular UMAP analysis using Mantel's test. Procrustes analysis was performed using the vegan package (v2.5-5) in R.

Statistical analysis

Statistical tests were conducted using R (v4.0.2). Nonparametric tests were used to compare two independent groups (Wilcoxon rank-sum test), two related groups (Wilcoxon signed-rank test) and multiple groups (Kruskal–Wallis test). A Spearman's rank-order correlation was used to carry out non-parametric correlation analysis. Fisher's exact tests were used to test the difference between two categorical variables. P values are from two-sided comparisons. P values for multiple comparisons were corrected using the Benjamini–Hochberg FDR method. $P<0.05$ or FDR-corrected $P<0.05$ was considered significant. All acquired data were included in analyses. Figures were generated using the R packages ggplot2 (v3.3.2).

Random forests

Random forest classification was implemented using the randomForest package (v4.6-14) in R. The decision trees were trained on the data consisting of bacterial relative abundance at the genus level (genera with abundances greater than 10% in at least one sample were selected) and small circular DNA viruses copy numbers (*Redondoviridae* or *Anelloviridae*). The samples from the first two time points were interrogated to control for greater sampling duration of sicker subjects; the sample with the highest commensal DNA virus level was selected from each pair. Binary variables, such as intubated or not intubated, were analyzed using classificatory random forest classification. Discriminating predictors were identified by random forest using importance values, which were calculated as mean decrease in Gini index for classification random forests. Bootstrapped iterations were performed to obtain an estimate of the misclassification rate. Receiver operating characteristic (ROC) curves, which plot the true positive rate versus false positive rate for all possible threshold probabilities, were generated by pROC (v1.16.2) in R.

Data availability

Sample information and raw sequences analyzed in this study are available in the National Center for Biotechnology Information Sequence Read Archive under accession IDs

PRJNA678105, and PRJNA683617 (Table S6). Computer code used in this study is available at https://github.com/BushmanLab/covid_microbiome_2021.

References for supplemental methods

1. Kelly BJ, Imai I, Bittinger K, Laughlin A, Fuchs BD, Bushman FD, Collman RG. Composition and dynamics of the respiratory tract microbiome in intubated patients. *Microbiome* 2016; 4: 7.
2. Everett J, Hokama P, Roche AM, Reddy S, Hwang Y, Kessler L, Glascock A, Li Y, Whelan JN, Weiss SR, Sherrill-Mix S, McCormick K, Whiteside SA, Graham-Wooten J, Khatib LA, Fitzgerald AS, Collman RG, Bushman FD. SARS-CoV-2 genomic variation in space and time in hospitalized patients in Philadelphia. *MBio* 2021; 12: e03456-03420.
3. WHO Working Group on the Clinical Characterisation and Management of COVID-19 infection. A minimal common outcome measure set for COVID-19 clinical research. *Lancet Infect Dis* 2020; 20: e192-e197.
4. Kim D, Hofstaedter CE, Zhao C, Mattei L, Tanes C, Clarke E, Lauder A, Sherrill-Mix S, Chehoud C, Kelsen J, Conrad M, Collman RG, Baldassano R, Bushman FD, Bittinger K. Optimizing methods and dodging pitfalls in microbiome research. *Microbiome* 2017; 5: 52.
5. Clarke EL, Lauder AP, Hofstaedter CE, Hwang Y, Fitzgerald AS, Imai I, Biernat W, Rekawiecki B, Majewska H, Dubaniewicz A, Litzky LA, Feldman MD, Bittinger K, Rossman MD, Patterson KC, Bushman FD, Collman RG. Microbial Lineages in Sarcoidosis. A Metagenomic Analysis Tailored for Low-Microbial Content Samples. *Am J Respir Crit Care Med* 2018; 197: 225-234.
6. Simon-Soro A, Sohn MB, McGinniss JE, Imai I, Brown MC, Knecht VR, Bailey A, Clarke EL, Cantu E, Li H, Bittinger K, Diamond JM, Christie JD, Bushman FD, Collman RG. Upper Respiratory Dysbiosis with a Facultative-dominated Ecotype in Advanced Lung Disease and Dynamic Change after Lung Transplant. *Annals of the American Thoracic Society* 2019; 16: 1383-1391.
7. Bolyen E, Rideout JR, Dillon MR, Bokulich NA, Abnet CC, Al-Ghalith GA, Alexander H, Alm EJ, Arumugam M, Asnicar F, Bai Y, Bisanz JE, Bittinger K, Brejnrod A, Brislawn CJ, Brown CT, Callahan BJ, Caraballo-Rodríguez AM, Chase J, Cope EK, Da Silva R, Diener C, Dorrestein PC, Douglas GM, Durall DM, Duvallet C, Edwardson CF, Ernst M, Estaki M, Fouquier J, Gauglitz JM, Gibbons SM, Gibson DL, Gonzalez A, Gorlick K, Guo J, Hillmann B, Holmes S, Holste H, Huttenhower C, Huttley GA, Janssen S, Jarmusch AK, Jiang L, Kaehler BD, Kang KB, Keefe CR, Keim P, Kelley ST, Knights D, Koester I, Kosciolek T, Kreps J, Langille MGI, Lee J, Ley R, Liu YX, Loftfield E, Lozupone C, Maher M, Marotz C, Martin BD, McDonald D, McIver LJ, Melnik AV, Metcalf JL, Morgan SC, Morton JT, Naimey AT, Navas-Molina JA, Nothias LF, Orchanian SB, Pearson T, Peoples SL, Petras D, Preuss ML, Pruesse E, Rasmussen LB, Rivers A, Robeson MS, 2nd, Rosenthal P, Segata N, Shaffer M, Shiffer A, Sinha R, Song SJ, Spear JR, Swafford AD, Thompson LR, Torres PJ, Trinh P, Tripathi A, Turnbaugh PJ, Ul-Hasan S, van der Hooft JJJ, Vargas F, Vázquez-Baeza Y, Vogtmann E, von Hippel M, Walters W, Wan Y, Wang M, Warren J, Weber KC, Williamson CHD, Willis AD, Xu ZZ, Zaneveld JR, Zhang Y, Zhu Q, Knight R, Caporaso JG. Reproducible, interactive, scalable and extensible microbiome data science using QIIME 2. *Nat Biotechnol* 2019; 37: 852-857.
8. Callahan BJ, McMurdie PJ, Rosen MJ, Han AW, Johnson AJ, Holmes SP. DADA2: High-resolution sample inference from Illumina amplicon data. *Nat Methods* 2016; 13: 581-583.
9. Quast C, Pruesse E, Yilmaz P, Gerken J, Schweer T, Yarza P, Peplies J, Glöckner FO. The SILVA ribosomal RNA gene database project: improved data processing and web-based tools. *Nucleic Acids Res* 2013; 41: D590-596.

10. Davis NM, Proctor DM, Holmes SP, Relman DA, Callahan BJ. Simple statistical identification and removal of contaminant sequences in marker-gene and metagenomics data. *Microbiome* 2018; 6: 226.
11. Charlson ES, Chen J, Custers-Allen R, Bittinger K, Li H, Sinha R, Hwang J, Bushman FD, Collman RG. Disordered microbial communities in the upper respiratory tract of cigarette smokers. *PLoS One* 2010; 5: e15216.
12. Charlson ES, Bittinger K, Haas AR, Fitzgerald AS, Frank I, Yadav A, Bushman FD, Collman RG. Topographical continuity of bacterial populations in the healthy human respiratory tract. *Am J Respir Crit Care Med* 2011; 184: 957-963.
13. Abbas AA, Taylor LJ, Dothard MI, Leiby JS, Fitzgerald AS, Khatib LA, Collman RG, Bushman FD. Redondoviridae, a Family of Small, Circular DNA Viruses of the Human Oro-Respiratory Tract Associated with Periodontitis and Critical Illness. *Cell Host Microbe* 2019; 25: 719-729.e714.
14. Liang G, Zhao C, Zhang H, Mattei L, Sherrill-Mix S, Bittinger K, Kessler LR, Wu GD, Baldassano RN, DeRusso P, Ford E, Elovitz MA, Kelly MS, Patel MZ, Mazhani T, Gerber JS, Kelly A, Zemel BS, Bushman FD. The stepwise assembly of the neonatal virome is modulated by breastfeeding. *Nature* 2020; 581: 470-474.
15. Mathew D, Giles JR, Baxter AE, Oldridge DA, Greenplate AR, Wu JE, Alanio C, Kuri-Cervantes L, Pampena MB, D’Andrea K, Manne S, Chen Z, Huang YJ, Reilly JP, Weisman AR, Ittner CAG, Kuthuru O, Dougherty J, Nzingha K, Han N, Kim J, Pattekar A, Goodwin EC, Anderson EM, Weirick ME, Gouma S, Arevalo CP, Bolton MJ, Chen F, Lacey SF, Ramage H, Cherry S, Hensley SE, Apostolidis SA, Huang AC, Vella LA, Betts MR, Meyer NJ, Wherry EJ, Alam Z, Addison MM, Byrne KT, Chandra A, Descamps HC, Kaminskiy Y, Hamilton JT, Noll JH, Omran DK, Perkey E, Prager EM, Pueschl D, Shah JB, Shilan JS, Vanderbeck AN. Deep immune profiling of COVID-19 patients reveals distinct immunotypes with therapeutic implications. *Science* 2020; 369.
